# Beyond the classroom walls: Stakeholder experiences with remote instruction in Post RN baccalaureate nursing program during the COVID-19 pandemic: A qualitative inquiry

**DOI:** 10.1371/journal.pone.0300007

**Published:** 2024-04-04

**Authors:** Laila Akber Cassum, Arusa Lakhani, Saima Sachwani, Zeenar Salim, Ridah Feroz, Shanaz Cassum

**Affiliations:** 1 Aga Khan University, School of Nursing and Midwifery, Karachi, Pakistan; 2 Syracuse University, Syracuse, New York, United States of America; 3 Humber College, Toronto, Canada; Tecnologico de Monterrey, MEXICO

## Abstract

The COVID-19 pandemic led to the closure of educational campuses and the suspension of conventional classroom teaching globally and locally, and many switched overnight to an online modality. The change was experienced differently by varied audiences, given the availability of resources. The study aimed to examine stakeholders’ experiences of emergency remote instructions in the Post-RN Baccalaureate Nursing Program during the COVID-19 pandemic. A qualitative descriptive exploratory design with a purposive sampling technique was used at a private nursing university in Karachi, Pakistan. Focus group discussions with students and faculty were conducted separately, while in-depth interviews with key informants were held using semi-structured interview guides. The focus group discussions and in-depth interviews were recorded electronically and transcribed and translated, coded, and analysed manually. Findings uncovered two major themes. (a) Remote teaching and learning—a paradigm shift; and (b) Remote learning ecosystem—a challenging team sport. The first theme denotes a major shift in pedagogical approach migrating from blended learning model to a complete online modality. Theme two uncovers the efforts and teamwork of the various stakeholders who assisted in mitigating the challenges collaboratively when migrating to virtual learning environment. Findings suggest that to continue to thrive in the post-COVID world, faculty, students, and key informants must collegially enhance the teaching, learning, and assessment strategies and student-teacher interaction, capitalising on evidence-based practices, trial and error, multi-level support mechanisms, and partnerships. The study recommends building resilience in instructional and administrative infrastructure to prepare for future events like pandemics and suggests development of evidence-informed blended and online nursing programmes in the region.

## Introduction

The COVID-19 pandemic and subsequent surge worldwide led to the complete suspension of conventional face-to-face (F2F) teaching. To continue educational activities during these unprecedented times, many higher education institutions globally switched overnight to online delivery of education, while others suspended F2F instruction temporarily until the availability of technological infrastructure. In lower middle-income countries (LMICs), including Pakistan, higher education is predominantly offered face-to-face. Besides, due to social distancing regulations during the peak COVID period, the faculty and students who worked mainly from home found online tools completely new, which was a challenging experience for them to handle. Initially, instructors began lecturing online, and the reduced student-student interaction made it difficult for the students to clarify their queries. This contrasted with face-to-face teaching, in later phases of pandemic, where students found relatively more opportunities to talk with their peers. Although evidence-informed online learning (OL) is reported to play a promising role in enhancing students’ interaction, motivation, and retention of content [1,2], inaccessibility of devices, limited or no connectivity, a lack of faculty and student technological preparedness, and a lack of expertise in instructional design in academia raise questions about the quality of the online educational experience in resource-limited countries [3–5]. and instructors during pre-, during, and post-class activities to seek support from each other.

## Literature review

Online learning has gained significant acceptance owing to its encouraging role in transforming the educational environment. Cost effectiveness, accessibility, flexibility, convenience, diverse digital applications, etc. are just a few of the countless benefits it has bagged in the past five decades [6–8]. Literature validates that the online modality when designed adequately is a useful and efficient approach to teaching students of nursing and medical disciplines. On the contrary, higher education in developing countries rely mostly on conventional classroom teaching, which is the predominant and most feasible mode of transferring knowledge, skills demonstration, and clinical practise [9,10]. In contrast, adequately designed OL is a considerable challenge for resource-limited countries due to a lack of infrastructure and advanced digital resources [11]. Moreover, technological glitches, inadequate technological training, and limited knowledge of online infrastructure result in low academic outcomes and dissatisfaction, further hampering teaching and learning connectivity between teachers and learners [12].

During the pandemic, there was a worldwide transition from face-to-face to online education that was mainly executed without sufficient teacher and student preparation [13]. In Pakistan, the exponential rise of COVID cases also resulted in a lockdown and work-from-home situation [14]. Universities and higher education institutions were directed to close their physical campuses and rapidly migrate to distance learning modes to lessen the spread of COVID [15,16]. Although this sudden shift allowed faculty to embrace the benefits of technology [17], it also surfaced issues of access to devices, a steady broadband connection, and a limited technological and pedagogical repertoire [18]. Various research studies conducted in India [19], the United Arab Emirates [20], Saudi Arabia [21,22], Nepal [23], and Midwestern Universities [24], report that rapid migration to OL was profoundly challenging for educators and learners in nursing and medical fields in developing countries. In Pakistan, more than 75% of the students in "high-ranked" universities critiqued COVID-imposed remote education because it lacked student-student and student-teacher interaction, and almost half of the students were not satisfied with their online experience [25,26]. Furthermore, findings from other research studies favoured face to face conventional teaching than online. Unavailability of electricity, inaccessibility of high-speed internet, lack of motivation, and teachers—students—institution preparedness were the common concerns that surfaced during the transition to online instruction [27–30].

The challenges identified by faculty, students, and management in higher education institutions in Pakistan included faculty’s inadequate knowledge and skills to teach and assess online, low or no internet bandwidth, a lack of study space at home, limited student engagement, and a lack of understanding of the pre-requisites of the overall online educational eco-system [12,13]. Students interacted more in synchronous sessions than asynchronous sessions and used synchronous teaching to clarify their misconceptions, where possible. However, compared to F2F instruction, synchronous sessions had issues such as limited exposure to students’ and teachers’ gestures, minimal eye contact, and difficulty reading the cues from the classroom atmosphere [13]. It is important to note that access to adequate study spaces, specialised hardware and software, and a steady internet connection varied due to the geographic and socio-economic status of the students [31,32].

Student and faculty discomfort with using a video camera and their mental and physical health were recurring challenges that the institutions faced with a change in public health emergencies [31]. In such a scenario, hybrid mode of instruction was practiced across institutions, where theoretical components are taught online and lab-based practical skills are conducted in labs, following social distancing protocols until the pandemic continued [9,31]. Despite the process being challenging, students reaped several benefits during the online transition: the ability to view the recorded lectures multiple times; students’ beginning to take ownership of their learning; and the flexibility of time and space to complete online modules [13]. A study conducted at a Middle Eastern university reported that part-time students attended more classes during this transition period than full-time students, possibly because of the increased flexibility and self-regulation that part-time non-traditional students could avail themselves of [13]. Nonetheless, faculty development around technological and pedagogical knowledge to ensure quality teaching and learning is suggested to mitigate the challenges of online teaching [12,25,33].

The Aga Khan University School of Nursing and Midwifery (AKUSONAM) is a non-profit private university operating in Pakistan and East Africa. The institution is the first-ever nursing department established at Aga Khan University in Karachi, Pakistan, in 1980. It offers undergraduate, graduate, and postgraduate programmes in nursing and midwifery. Among the three undergraduate programs, blended learning (BL) was first introduced in the Post-Registered Nurse Baccalaureate of Science in Nursing (Post-RN BScN) in 2010. In 2020, with the closure of on-campus academic activities, a sudden and rapid shift to online instruction was initially implemented in this program. There is adequate technological infrastructure at AKUSONAM, Karachi, Pakistan, and the faculty (teaching staff) are provided with technical support to undertake blended instruction, combining synchronous and asynchronous teaching modes on campus.

Network of Quality Teaching and Learning (QTL-net) provides university-wide pedagogical support provided through faculty development and quality assurance activities, and support faculty and other stakeholders in promoting excellence and ensuring a robust student learning experience. Nevertheless, in this rapidly evolving and changing pandemic, it was an immense challenge for SONAM technological support and QTL_net teams to meet the increased demands for seamless delivery of online programmes and facilitate faculty and students’ ability to teach and learn remotely while working from home and using home-based devices with inadequate broadband connections. In addition, access to devices, internet connectivity, and a steady power supply for all students and faculty, particularly those (60%) who were residing in the northern areas of Pakistan, posed problems. Despite having experience with blended learning, remote learning was a completely new phenomenon in the transition from BL to OL for nursing students and faculty. Although online teaching during covid 19 pandemic is associated with positive and negative experiences for all stakeholders of education process, little is known about these experiences in nursing education programs in LMIC. Therefore, it became important to understand the experiences of students, faculty, and key informants regarding remote instructions and assessments during COVID-19, to fill the gap and provide essential perspectives to the literature.

## Study purpose

To guide the development of future online programmes at AKUSONAM and in the country, it was essential to explore students’, faculty’s, and key informants’ experiences regarding remote instruction and assessments during COVID-19. The study questions were (1) What are the perceptions and experiences of students, faculty, and key informants regarding the rapid shift to remote instruction during COVID-19 in the Post-RN BScN program? (2). What are the benefits, challenges, and recommendations of remote instruction and assessments experienced by the students, faculty, and key informants in the Post-RN BScN programme during the COVID-19 pandemic?

## Methods

### Study design

Exploring the perceptions and experiences of remote course instruction among nursing students, programme faculty, and key informants involved in the Post-RN BScN programme requires a descriptive qualitative exploratory study design. Qualitative inquiry is a naturalistic method that enables researchers to get acquainted with diverse perspectives on a phenomenon of interest and to communicate the experiences of those who have experienced it. In addition, a qualitative methodology investigates the various facets of individuals’ life experiences and interprets them from the individuals’ perspectives [34–38]. The selection of qualitative research is based on the fact that the participants must have firsthand knowledge of the phenomenon of interest to fully comprehend the concept [39]. This study is a qualitative investigation that is anticipated to yield significant knowledge and insight. Aga Khan University served as the study setting for this research. Ethical approval was obtained from the University Research Ethics Committee, which was in line with the principles of the Declaration of Helsinki and assigned the reference number 5453. Administrative approval for data collection was sought from the school. Following ethical approval, the research team sent an electronic invitation to study participants along with a consent form and a demographic form. The return of the signed electronic consent form via email was considered proof of their willingness to participate in the study. The calendar invites and zoom links for interviews on mutually agreed dates and times were agreed upon after receiving the participants’ informed consent and demographic details.

### Sample

This investigation employed maximum variation purposive sampling technique. Purposive sampling helps in the selection of information-rich cases for the study [40], as examining such cases yields insight and a comprehensive understanding of the research phenomenon [41]. While maximum variation method aimed to obtain a comprehensive understanding of the complete online teaching and learning modality during the pandemic by selecting representative and diverse participants including students, faculty members, teaching and learning key informants, and IT specialists [42]. In qualitative research, there is no rule for determining sample size [40]. Nevertheless, the sufficiency of the data to validate the themes is more crucial than the predetermined number of participants [36]. The study population included post-RN BScN nursing students (n = 50), faculty teaching in the programme (n = 17), and key informants (n = 4). The sample comprised post-RN BScN students (n = 23), faculty members (n = 8), and key informants (n = 4) that includes curriculum committee member, academic leader, QTL_net staff and e-learning support staff. Two of the 23 students dropped out due to their professional responsibilities at the hospital. The inclusion criteria for this study were participants’ eligibility, consent, and willingness to participate in the study. Eligibility was determined by two conditions participants are1) Participants are associated with Post-RN BScN Program as students, faculty, or key informants 2) Participants have experienced online instruction and learning during covid. The exclusion criteria were individuals who were unable to provide consent.

### Data collection

Data were collected from December 2020 to March 2021 through a demographic survey, focus group discussions (FGD), and in-depth interviews. The demographic survey included information on age, gender, marital status, clinical experience, current residence, type and access to the internet device, its quality, and level of satisfaction. Three online FGDs were conducted, two with students and one with the faculty, ranging from 60 to 90 minutes, whereas four indepth interviews were carried out with the key informants, who were experts in teaching and learning, curriculum development, e-learning, including QTL-net faculty developers and SONAM administrative staff for around 45 minutes. Data collection was stopped once saturation was achieved.

All interviews were conducted by two research team members who were not known to the participants. In FGDs, one was the moderator and the other was a note-taker, while the interview and note-taking were done by a team member. The interview guide included open-ended and probing questions. The guide consists of 7–8 questions exploring participants’ experiences and perceptions of remote learning. It delves into the transition from blended modality to remote curriculum teaching and learning, examining facilitating and hindering factors associated with remote learning, The questions also address challenges related to the remote teaching-learning process and assessments, concluding with participants’ recommendations to enhance online teaching and learning. The guide was pilot tested on 10% of the population to identify potential practical issues and voids. Data from the pilot test was not included in the actual data. The interview guides were found to be appropriate, and no modifications were made.

The interview began with greetings, an interviewer introduction, and sharing of the study purpose. In addition to receiving signature from participants prior to the interview, study details were again shared to ensure comfort and ease. The confidentiality and anonymity of the participants were ensured by assigning pseudonyms to de-identify them. Transcripts were developed from the FGDs and in-depth interviews.

### Study Rigor

Rigor was ensured by using Lincoln and Guba’s [43] four criteria for preserving the trustworthiness of research. The credibility of the findings was established through the process of data triangulation, which involved the integration of multiple sources of data and the inclusion and the inclusion of feedback from variety of stakeholders. This enabled us to cross- verification and gain a full understanding of the phenomenon under investigation. Dependability was established by the utilization of investigator triangulation, wherein the viewpoint of an expert was considered. On the other hand, confirmability was produced by arriving at a reasonable conclusion through rationalised discussions conducted within the research team. Furthermore, it is noteworthy to mention that out of the six researchers involved in the study, four of them were nurse educators. Consequently, the potential biases stemming from their professional backgrounds were thoroughly acknowledged and carefully considered during the analysis process. The team convened regularly to engage in constructive deliberations on divergent viewpoints.

### Data analysis

Data was collected and analysed simultaneously following the criteria established by Miles & Huberman [44]. Extensive content analysis was conducted by reading and re-reading transcripts, word by word and phrase by sentence to fully understand the material before dividing it into portions. The texts for each question were organized in a three-column format: the left column was titled “codes”, middle one as “informants verbatim” and the right one as “thoughts and comments while reading text”. Codes were generated from the data related to each question. In-vivo codes were identified by color-coding them and assigning them a section label, term or phrase. These codes were written on transcript margins to detect any duplication. Coding all transcribed interviews resulted in the clustering of relevant codes into groups and subcategories. Finally, themes that matched the subject of the text were extracted. The team verified the generated themes and categories until a mutual agreement was achieved.

## Study findings

### Demographic survey findings

35 participants contributed to the study. There were a higher number of females (n = 28) in the sample compared to males (n = 7), which is representative of the overall population in the school. Faculty, students, and key informants regarded the transition to remote instruction as a complete paradigm shift because they learned how to engage in fully online instruction and assessment through experimentation. Faculty and students used the Virtual Learning Environment (VLE), video-conferencing software (MS Teams and Zoom), and interactive software (Kahoot, Mentimeter, Google Documents, and Padlet) to continue teaching. WhatsApp and Zoom breakout groups facilitated asynchronous and synchronous peer-learning experiences. Faculty highlighted interruptions in internet service and the resulting discomfort while teaching from home. The student faced multiple issues, including internet disruption, non-preparedness to learn online, unavailability of study space at home, and extensive time and cost for travelling from the internet hub to home.

Most of the students (96%) and faculty (86%) were satisfied and showed their contentment with the technical support provided by the institution. Also, key informants (75%) expressed their satisfaction with the support they provided during the process. However, research participants had varied views about receiving training to continue online learning during the disruption caused by the pandemic. 78% of students shared that they lacked pedagogical training when they moved online; however, faculty (88%) and key informants (75%) acknowledged professional development in online training, and the institution provided support for online teaching. Most faculty (88%) and key informants (100%) were supportive of a fully online programme post-covid as compared to students (39%).

### Themes and categories

Two major themes emerged from the qualitative analysis of the data (a) Online teaching and learning—a paradigm shift; and (b) Online learning ecosystem—a team sport. The detailed explanations of both the themes and the categories are provided below, along with the excerpts from the study participants, labelled as SFGD (Focus Group Discussion with students), FFGD (Focus Group Discussions with faculty), and IIKI (In-depth interviews with key informants).

#### Theme 1. Remote teaching and learning—A paradigm shift

The theme one which is also referred as a “Paradigm shift” indicates a major transformation in pedagogical approach from blended to remote modality. The pandemic was an unprecedented event that required an immediate conversion of curriculum delivery (teaching, learning and assessment) to a complete online mode. Though, the blended mode already existed pre -covid, only 30% of the delivery was conducted online while 70% functioned face to face. In that 30%, learning resources such as syllabi, readings, instructional videos, and assignment guidelines and submission were required through the learning management system (Moodle). Delivery of Instruction and Quizzes were taken in face-to-face manner. During covid, advanced technological tools such as video conferencing, virtual tele simulations and gaming applications were used. Additionally, this flexible methodology facilitated the participants from diverse backgrounds and places to access instruction. The reasons for this shift are delineated in detail in the categories that arose during the analysis.

Participants expressed that this remote instruction was a complete paradigm shift in many ways: (a) multifarious perceptions regarding readiness to use remote instruction; (b) teaching learning strategies; (c) formative and summative assessments; and (d) student-teacher interaction and engagement.

***Category 1*.*1*: *Multifarious perceptions of remote instruction -Are we ready for it*?** The participants described their diverse perceptions of remote teaching during the pandemic. Some students felt it to be a blessing in disguise and an opportunity in difficult times, whereas other students felt it distressing, demanding, and distorting their work-life balance. Most participants considered online teaching and learning effective, creative, contemporary, and timely during a pandemic.

Students’ perspectives: Students had varied experiences with remote instruction, for example, one of the students expressed her optimism as:

"*It was an excellent thing*…*the studies continued in our university*…*and we were encouraged by the faculty… we had experienced the blended approach*…*and now it is 100% online…accessible from anywhere*."

(SFGD)

However, some students found remote instruction during covid pandemic as disturbing, due to challenges in work—life balance. One of the students exclaimed, *“I feel it was challenging to follow the schedule… constant emails*, *messages*, *and materials were sent … and it was very disturbing*.*”* (SFGD)

Faculty perspectives: Faculty considered the adaptation to remote instruction overwhelming. A faculty member expressed:

"*Change is always difficult*… *this will take time*, *so we must follow the trend and choose whatever is the most viable and feasible option for teaching during the pandemic*. *It has become a necessity and a must to do*.”

(FFGD)

Key informants Perspectives: remarked on the flexible and cost-effective nature of remote teaching and how it removed the barriers of time and space. One key—informant shared:

“*Online teaching*-*learning is one of the recent contemporary modes where distance and physical presence are not barriers to higher education*. *It is a flexible*, *uninterrupted approach that enables anyone to pursue education*.”

(IIKI)

The varied levels of comfort amongst the faculty and students with adapting to remote learning and key informants’ hope for continuing online education, imply the need for personalized support to transition to online learning when planned in the future.

***Category 1*.*2***: ***Teaching and Learning Strategies***–***A supported journey of trial and error***.

**Students Perspectives**: Many students reported that they had gained technological and study skills as they switched from a blended to an online medium. A student stated:

“*Much of my online computer skills had been polished*. *My habit of learning has also improved; during COVID*, *I did more self-study and felt connected with my studies*."

(SFGD)

A few students shared their concerns about faculty needing to be more proficient in their technical skills, to adequately support student learning. They also suggested appropriate digital tools to the faculty for more engaged learning. A student narrated her experience and stated:

“*It is sometimes very challenging for the faculty to learn the digital application and implement it in the classroom because the digital apps need technicality and good hands-on*…*we suggested some game-based app to the faculty for our classes*….”

(SFGD)

**Faculty Perspectives**: The faculty reported using multiple teaching-learning strategies with students during COVID. Most of the faculty conducted synchronous sessions via Zoom-based video conferencing to replace face-to-face lectures and showed continued concerns about student engagement. These strategies included polling, cooperative learning exercises, online post-it boards, and pop quizzes. Software such as Zoom, Microsoft Teams, Mentimeter, Kahoot, and Padlet were used. Faculty became more confident in using these digital tools through trial and error and support from their peers and e-learning staff. A faculty member narrated her experience of using quizzes through a game-based application in the following words:

“*Kahoot* …*was also very much appreciated by the students because they could use their mobile phones to engage in the game activity*.”

(FFGD)

Interestingly, the pandemic surfaced the need to support each other in active experimentation with online tools and pedagogies which strengthened relationships between students and faculty. For example, students assisted faculty by resolving technical issues during instruction. A faculty reflected and exclaimed:

“*Online teaching requires much preparation to design a course for which most of us are not trained*. *Whatever we are doing so far in online teaching is by hit and trial*.”

(FFGD)

Some faculty members struggled with technology; however, others were exploring technologically advanced pedagogies to support student learning. For example, a faculty team used virtual tele-simulations using software applications such as Cyber Patient and Body-Interact for clinical courses. A faculty member opined:

“*We used tele-simulations and other software to interview a virtual client and hypothesize the patient’s problem*. *Students verbalized this simulation as highly effective for learning interviewing*…*despite being unable to attend a real client*."

(FFGD)

Most faculty expressed that simulation-based remote clinical teaching was far more challenging than on-campus clinical teaching. Especially the emotional aspect of patient care was difficult to teach and learn. A faculty member explained:

“*In face-to-face clinical*, *we used to have a more emotional bond with the students*…*and we could see their emotions*, *anxiety*, *and fears regarding patient care*. *However*, *connecting with the students was difficult in the remote clinical*."

(FFGD)

Key informants’ perspectives: Department leadership were supportive and encouraging for faculty and students to continue their instruction. For example, leadership provided resources such as USBs with content and negotiated with partner institutions for an internet connection at central hubs. QTL_net and e-learning team continued to provide professional development such as trainings and workshops for faculty and students to adapt to this change. The nature of support is further discussed in theme 2.

Faculty and student experience of online teaching and learning were varied, and those who had an edge in learning technology faster through trial and error, were leveraging technology to enhance student engagement in learning, however, other faculty struggled with basic technological tools. This necessitates the need for a personalized support system for all faculty to be willing to experiment and learn to teach with new technological applications and engage students as partners in their learning process. Key informants such as departmental leadership, department-based e learning unit, and QTL_net ensured this support and professional development to faculty and students.

***Category 1*.*3***: ***Online Assessments***–***An Uncharted Journey***.

Assessing students’ learning online was an uncharted journey for faculty and students. Students and faculty highlighted challenges related to academic integrity, grading in group assignments, and skills required to create online assessments.

Students’ perspectives: Students indicated challenges with timed tests and group assignments. Students shared their apprehension towards subjective questions due to timed exams and stated being more comfortable with objective questions. In contrast, few shared their preparedness to take online subjective assessments. A student disclosed:

“*It was very difficult for me to type so fast in a given time and to complete the paper and submit it on time*…*I think MCQs is a better online strategy as compared to the subjective paper*.”

(SFGD)

Grading students on an individual or group assessment is essential to teaching. Almost all students expressed their dissatisfaction with working on group assignments during their online studies. Students found it very challenging to meet and discuss group work, especially those who were working part-time or living in remote areas, resulting in some students contributing more and others taking a free ride. A student expressed:

“*If group work needs to be done*, *then we should be allowed to make our groups more effective*. *One or two do the whole work in group assignments*, *taking the burden*. *So*, *the grading should be justified based on the efforts made by each individual student*….”

(SFGD)

Faculty Perspectives: Timely completion of the course requirements became more challenging with the periodic resurges in COVID cases. The faculty had prior experience conducting proctored VLE-based exams on campus. However, by switching to remote assessments, faculty learned new skills. A faculty member conceded:

“*I knew how to prepare online quizzes and assignments and upload them on the VLE course* site, but *when it comes to online tests that are time-bound*, *which have a certain number of questions*, *and then they have to be offered remotely*,…*this required a whole lot of skills in which I needed training in*.”

(FFGD)

Initially, faculty were apprehensive about creating flexible assessments such as open-book exams. Their apprehensions were rooted in the concern that students would ‘Google the answers’, thus questioning academic integrity. A faculty member voiced her concerns:

“*This is the first-time students are writing open book exams*… *I fear giving non proctored assessments and grading them*. *There may be inflated grades*.”

(FFGD)

Key Informants Perspectives QTL Net and in-house professional development provided faculty with support and professional development, because proctored exams were not feasible. Subsequently, faculty redefined the purpose of assessments, and the department implemented the directives given by the Higher Education Commission (HEC) and permitted the use of open-book exams, scenario-based case studies with critical thinking questions, and reflective papers, which were more feasible for students and the institutions with low bandwidth internet connections in the country. One participant explained:

“*We shared HEC guidelines and international best practices for conducting assessments in limited internet speed and poor connectivity settings*. *The revised assessment plans were approved by the curriculum committee*, *considering our context*, *student needs*, *and accessibility*.”

(IIKI)

Students and faculty shared their challenges with designing and conducting online assessments. Key informants supported by providing professional development opportunities and resources to help them gain technical skills in these challenging times.

The quest for developing authentic assessments was revisited, and stakeholders suggested using more formative assessments (for learning) compared to summative assessments (for learning).

***Category 1*.*4*: *Student-Teacher Engagement***. The participants reported that remote instruction was difficult due to the limited physical presence of faculty and students in a F2F instructional setting.

Students Perspectives: Students shared varied perspectives on learning virtually via zoom. A few students overcame their fear of participation and were more interactive in online class. One student stated,

“*Some of the students were quite shy and never participated in the class*. *During Zoom classes*, *they showed improvement*. *Even though we were unable to see their body gestures*, *their engagement has noticeably increased*.”

(*SFGD*)

In contrast, some students highlighted that they got distracted in online classes, because they could do multiple tasks at a time, while turning their cameras off. One student stated,

“*Face-to-face instruction allows us to communicate with our teachers directly*, *which helps us stay focused*. *We frequently got distracted in our online classes*. *Skipping classes and turning off our mics caused us to become distracted*”

(*SFGD*).

Faculty Perspectives: Faculty also expressed their discomfort with the inability to watch students’ body language, gestures, and reactions to the content and discussion. Especially in high-enrolled classes, faculty felt disconnected from students. In cases where students did not turn on the video cameras, a feeling of doubt about their presence on-screen emerged, especially when they did not respond when teachers called their names. In the words of a faculty member, *“Somehow*, *I was able to transfer the content*, *but I feel that in online teaching*, *we lose out on other things like the student’s emotions*, *the physical gestures*. *We cannot understand certain temperaments*, *which are essential for teaching*.*"* (FFGD)

A faculty member shared her concern about the holistic engagement with learners in the remote classroom:

"*Sometimes they are joining in*, *appearing in the participant list*, *but they are not there*…*they log into the class*, *and then they are not physically there and also not paying attention*…. *We can hardly manage these things in the online modality*.”

(FFGD)

Faculty ensured engaging students use multiple communication channels besides Zoom calls to strengthen the relationship and respond to students’ queries. For example, some faculty reported using ’*WhatsApp groups* to “*keep the connection alive*" and making phone calls to the students to know more about their learning trajectories, especially when the students were not active on WhatsApp groups and had no internet connection.

Key Informants Perspectives: Key informants had similar views about student-teacher relationship and highlighted the difficulty in establishing deeper student teacher relationships in fully online environments. One of the key informants, stated,

“Three interactions are crucial to teaching: students interacting with peers, students *interacting with content*, *and teacher-student relationship*. *Even in a large class*, *every student needs to have a personal relationship with the instructor*. *These relationships are compromised in the online setting*, *particularly those between students and the individualized relationship with the teacher*"

(*SFGD*).

Students, faculty, and key informants had a similar view about challenges in establishing meaningful student-teacher interaction and relationships, because in most instances, students’ body gestures and reactions were not observable. Some students indicated that they were distracted because of teachers were not observing them. Faculty noted making efforts to continue the interaction beyond the classroom sessions through WhatsApp groups and phone calls.

#### Theme 2: Remote learning ecosystem—A challenging team sport

Theme two referred to as “a challenging team sport” uncovered that all the transition to an online environment posed significant difficulties and challenges for all the stakeholders involved. For certain individuals it was perceived as valuable and presented a potential for growth. While for others it disrupted the equilibrium between work, school, and personal life. The participants identified multiple layers (such as leadership, faculty, e—learning unit in the entity and QTL_net) that substantially supported in rapid transition to virtual mode and led to continued education, despite varied levels of access to steady internet and technological devices. This theme metaphorically described the multitude of support that was central to transitioning online in a resource-limited and pandemic-challenged environment. The participants rich experiences have been further divided into categories which is explained below in details.

***Category 2*.*1*: *Family and Community based support and home infrastructure***. Although participants considered remote learning overwhelming, their experiences were mediated by their geographical locations, roles and support structures in the family, the availability of devices, and steady internet connections. Students living in Karachi and mountainous regions had diverse expressions.

Perspectives from Urban areas:

The participants from Karachi indicated the absence of appropriate study spaces and time, which resulted in struggles to manage multiple home responsibilities. However, some verbalized immense family support, due to which they could multitask at home as a parent, homemaker, and student. Time and space flexibility were possible due to the online mode and were appreciated by some participants. One student commented:

"*My baby used to be in front of my eyes*, *so I did not have to worry about her*. *Although the baby used to disturb me during classes*, *I could look after her too*. *I did not need to travel to the university and could study in my own time*… *often at midnight when my baby would sleep*”

(SFGD).

Few students encountered challenges managing online classes with added home and family responsibilities. A student admitted:

“*I used to find it much more difficult with the baby*. *She used to take the whole class along with me*, *and I could not listen properly as she used to pull out one earphone from my ear and ask what my teacher is talking about*, *and she wanted to listen to her too*. *So*, *I was very disturbed*. *I could not focus*.”

(SFGD)

Students living in remote areas expressed different concerns than those living in Karachi. Students living in Karachi found the online option to be cost-effective. As one student expressed:

“*The benefits of online classes were that we did not have to bear the printing cost*. *We submitted our assignments online*, *and no hard copies were required*. *We did not need to travel to campus every day to attend classes*.”

(SFGD)

Perspectives from mountainous areas

Students in mountainous northern areas bear the additional expense of travelling to the assigned central hubs to access the internet. A student reported:

“*We had to travel 60 kilometres for an internet connection*. *We used to get tired*, *and extra fuel was consumed for the travel*, *and then we also had to eat outside*. *In addition*, *there was a high risk of exposure to COVID-19*."

(SFGD)

Internet issues and power failures were problematic during remote classes and assessments. Students from mountainous regions faced commuting challenges to reach the central hubs due to a lack of transportation during the lockdown and extreme weather conditions. Also, heavy rainfall and frequent power failures added to their challenging experience. A student lamented:

“*It happened one day that there was an exam*, *and the internet facility had stopped*. *So*, *I had to travel 50 kilometres on a motorcycle to reach another site*… *it has been very difficult for me*. *I experienced physical and mental fatigue from travelling to the central hub for a stable connection*."

(SFGD)

Faculty Perspectives: Faculty acknowledged that the students were provided with extensive support from their families. One faculty member specified:

“*They could not move alone and needed a male family member with them to bring them to an assigned school where they could have access to the internet*.”

(FFGD).

Key informants’ perspectives: Key informants acknowledged challenges faced by students and faculty and ensured their support in addressing these issues. As one administrator shared:

“*You can balance your job and your studies*, *with your home responsibilities*… *different time slots could be utilized for studying purposes…administration’s support is with you*.”

(IIKI)

Students from urban areas highlighted work life balance as their primary issue where they had to balance their family expectations while continuing to take full study load. However, students from mountainous areas travelled a long distance to secure internet connectivity. Given the safety issues, female students were escorted by their elder siblings and parents to reach the central hub or internet stations. They also highlighted the financial cost associated with long travel and accommodation. Faculty and key informants were aware of these challenges and provided support and flexibility to address these challenges.

***Category 2*.*2*: *Department-based support***. A complete transition to remote learning was only possible with department-based support structures. Students, faculty, and key informants discussed the support provided by the department to improve skills needed to learn and teach online.

Students’ perspectives: Students highlighted improvement in their time management skills and talked about accommodations provided by faculty during their difficult times. One student acknowledged,

*Our faculty* “*taught us time management skills*. *Our faculty helped*, *even in difficult situations at home*. *I remember a time when my father was unwell during my Bio-Stat exam*. *Even under those conditions*, *my teacher pushed me to give it my all*, *and I did*. *They also showed flexibility in their approach by extending assignment deadlines when necessary*. *We were able to successfully handle our work and personal responsibilities throughout the pandemic*”

(*SFGD*).

Faculty perspectives: Faculty and students spent extra hours and effort to make this migration possible. E-learning support teams, key informants, and curriculum committee members facilitated various workshops on using technological applications for faculty members to ensure a smooth transition to remote learning. Faculty underscored the need for continued support to teach online.

“It took a lot of preparation to get ready for online teaching because the majority of us had no professional experience in this field. Even when we went to workshops, they weren’t enough. Creating successful online courses requires more extensive institutional support, which is necessary for the success of online learning."

(FFGD).

Key informants’ perspectives: The departmental leadership was also actively engaged in the faculty’s professional development. One of the key informants said:

“A *lot of institutional and management support was required to prepare faculty through continuing education sessions to respond to the need…many of them were not prepared for the online pedagogy*.”

(IIKI)

Students and faculty underwent extensive professional development sessions. Students commended faculty’s flexibility and support and acknowledged that they were also undergoing a learning curve. Faculty and key informants also highlighted the lack of pedagogical skills to teach online and underscored the importance of continued professional development.

***Category 2*.*3*: *University-based support***. The Quality, Teaching and Learning Network (QTL_net) and university leadership organized varied levels of support such as a) administrative support established through sending USBs to students and b) negotiating with partner institutions to establish central internet hubs, and c) academic support through ongoing professional development sessions.

Students’ Perspectives: The faculty packaged learning content and password-protected assessments through USBs for the students living in mountainous areas. In addition, central hubs were created with the local schools in Northern Pakistan for students to access the internet for learning and assessment purposes. One student said,

"*Our university (department) gave us much support during the pandemic time*. *They provided us with all the teaching material and assessments on USBs*, *which is why we could learn*. *If this support had not been with us*, *then it would be impossible to complete the course objectives*”

(SFGD)

Faculty’s perspectives: Faculty recommended that going forward, they expect to get financial support to purchase of premium version of digital tools such as padlet and kahoot. One faculty member stated,

“*Looking ahead*, *institutional assistance becomes even more important as learning increasingly moves online*. *The organization must think about spending money on resources like Padlet and Kahoot*, *which provide both premium and free services*. *These resources need to be available to improve the efficiency of our online education and learning*”

(*FFGD*).

Key informants’ perspectives: QTL_net organized ongoing professional development sessions for the entire university faculty in pedagogy, curriculum, assessments, and the application of technologically enhanced instruction. This was done through (a) workshops on instructional skills and course design; (b) boot camps with digital tools; (c) an ed-tech lounge for faculty to learn from each other’s practice; and (d) one-to-one support for faculty as they converted their instruction from F2F to online. One of the key informants explained:

“*The faculty were optimistic to learn*, *and their positive attitude helped us to conduct workshops on online assessments and best practices for online teaching and learning with the help of the* Quality *Teaching and Learning Network*.”

(IIKI)

The university played an exemplary role in building the capacity of the faculty members through their training boot camps and Ed—Tech lounges. One of the stakeholders reported:

“*Through best practices*, *the aim was to equip faculty with the skills to use digital technology associated with teaching and learning in their online classes*.”

(IIKI)

The university leadership and QTL_net provided administrative and academic support to the students and faculty to assure the continuation of instruction in the face of pandemic—related challenges.

## Discussion

Our study revealed that the students, faculty, and key informants found complete transition to remote instruction to be demanding because of various challenges such as (a) access to technology and internet with respect to students’ geographical locations, (b) funds and escorts to travel to central hubs and purchase of study materials, (c) students’ and faculty’s home responsibilities and work—life balance, (d) students classroom presence and engagement, (e) faculty’s prior technological and pedagogical skills, (f) assignment design, grading, and academic integrity.

The study reported the intersecting challenges that students faced in online instruction during pandemic times. Online learning was challenging for participants living in Karachi and in mountainous areas. While students in Karachi saved on accommodation and travel costs by studying at their homes, many of them noted difficulties in managing household tasks and maintaining work life balance. The findings are consistent with the study conducted with nursing students in Nepal and South Africa with a multidisciplinary team [23,45]. Experiences of students varied, because of the extent to which their families supported them in household chores, where some female students who were married and had children juggled with more house-hold responsibilities than others. The study reveals that despite the heavy engagement of female students in household chores and having limited time to focus on their studies, female students still felt that the online medium was more flexible mode of learning during the pandemic. It allowed them to continue learning after the family and household responsibilities were completed, especially in the late evenings and nights. These findings raise questions about the gender differences in parenting and household responsibilities, indicate areas for further exploration, and suggest a flexible educational approach for female students juggling multiple responsibilities [46].

In contrast, students in mountainous regions noted low internet connectivity and the unavailability of devices made the learning materials inaccessible. These students struggled to reach central hubs because of transportation and financial issues. Female students required escort from their elder siblings and parents to reach the central hub or internet stations to ensure safety. These students did not mention struggling with household chores. These findings are congruent with other studies [15,23,47], emphasising lack of internet access and the availability of devices makes the remote learning more challenging.

Faculty and students faced challenges in developing classroom engagement and student-teacher relationships. Faculty verbalised fears and dilemmas regarding students’ presence, whether they were physically present or just logged in to mark their attendance. When the teachers could not see their faces, they felt detached from the students. This may be because of the discomfort caused by the lack of students’ cognitive presence, suggesting a disequilibrium in trust in student-teacher relationships [46,48]. The study also found that WhatsApp groups were commonly utilised as a communication tool due to its ease of use and accessibility compared to Learning Management System. However varying technological skills affected the quality of teaching and student engagement. Faculty with higher technological skills were able to engage students using varied technological tools, as compared to those that were struggling with technology. These findings are congruent with other studies [15,23,49], emphasising that a lack of technological skills act as a significant barrier to effectively continuing online education. Additionally, concerns around students’ and faculties’ privacy must be considered before choosing WhatsApp as a core communication tool in the post-Covid times [50].

Faculty and students also highlighted challenges with design and implementation of assessments. Students favoured short answer questions and multiple-choice questions (MCQs) for assessments over long essay questions. This preference was because extended response questions assessed content knowledge but also tested typing speed under time pressure, a point also discussed in other studies [51,52]. Faculty participants also showed concerns regarding academic integrity. Research suggests that academic integrity must be ensured by taking measures to discourage cheating when proctored exams are not possible. Nevertheless, this highlights the need to explore other non-proctored assessment modalities (such as designing open-book exams, scenario-based questions, vodcasts, podcasts, and iterative and incremental assignments) that ensure the quality and authenticity of the examination [53,54].

Partnership between students, faculty, and key informants resulted in varying levels of support such as (a) sending content through USBs in remote areas (b) establishment of internet hubs through partnering with schools and colleges in the community (c) professional development and support (d) faculty’s flexibility and accommodation of students’ needs (e) WhatsApp group and phone calls exchange between students and faculty helped in continuing the education, during pandemic. These supports helped in re-establishing the relationship between students and faculty and resulted in continuing education during pandemic.

Institutional support supplemented with in-house departmental support and professional development is critical to integrating technology and adapting e-learning approaches [50]. In this study, students shared mixed experiences with the actions carried out by the department and university. Especially the students from the remote areas acknowledged the efforts made to provide customised study resource packs at their doorsteps and arrangements for the local private schools to operate as internet hubs. Faculty also acknowledged the support and professional development provided by the e-learning team and QTL_net. Examples included orientations, Ed-tech lounge, bootcamps on various educational technology tools [17]. However, more support in terms of training and resources for faculty and students was required to make technologically leveraged education, smooth and effective. Online teaching was implemented as an alternative to traditional in-person instruction during the COVID-19 pandemic [55]. However, its effectiveness and sustainability for all students was questioned [48,56]. The factors discussed in the current study suggest that it is important for stakeholders to determine how to best equip the students, instructors, support mechanisms, and necessary resources to succeed in the online learning environment in the future.

As a result of partnership between students, faculty, key informants, professional development unit, families, and community institutions during pandemic, students and faculty reported to continue their remote instruction, despite various challenges reported earlier. Faculty and students noted upskilling themselves. Students appreciated and supported faculty’s flexibility in understanding students’ needs and providing relevant accommodations. They also commended use of e-learning tools and WhatsApp groups to continue instruction and maintain student-teacher relationship. Specifically, use of virtual simulations emerged as a promising strategy to teach clinical skills was commended; however, faculty and students underscored the necessity of patient-student interaction in a bedside setting. These findings are aligned with the studies [57–59] which stated that online education could substitute the theoretical elements of nursing education; however, interaction with real patients, systematic demonstration, and hands-on practise are important elements that can be effectively achieved via a hybrid learning environment.

Students and faculty noted upskilling themselves to continue remote instruction. For example, students reported improvement in their study habits and time management skills. Students also appreciated faculty’s use of e-learning tools to continue instruction.

Specifically, use of virtual simulations to teach clinical skills was commended; however, they underscored the importance of patient-student interaction in a bedside setting.

## Implications and recommendations

Covid-19 necessitated rapid changes in instruction at the school of nursing under study, but broadly to nursing education. The study presents three key implications and recommendation for institutions in developing countries to consider:

1. Preparedness for teaching and learning online in emergencies: We learned that students and teachers both need personalized support to learn and teach online, customized to their prior skillset and needs. While some faculty are more experimental and try out clinical simulation software and e-simulations to support clinical teaching, other faculty struggled in engaging students during instruction, therefore, provision of personalized need-centric technological and pedagogical support is necessitated, in offering remote instruction. Moreover, institutions need to prepare their faculty, staff, and faculty development units to offer support during emergencies, so that instructional decisions are based on tried and tested models of emergency education, and not a result of afterthought and complete trial and error. While we acknowledge that trial and error helped students and faculty to continue education during the covid pandemic, basic preparedness is necessitated to avoid faculty and student burn out during emergency situations. In addition, we also realized the necessity of mental health and resilience support during emergencies for faculty, students, and staff to cope with the challenges that emergency situation presents.
2. Strengthening institutional support: We learned about the instrumental role that cross-entity and department-based support plays in equipping faculty with necessary skills to teach and students to learn online. The cross-entity support, in our case, was provided by QTL_net that enabled cross-disciplinary discussions about online pedagogy and assessments, and department-based support was provided to nursing faculty by the nursing school to enable them to design customized experiences for the nursing students, for example, collecting the educational materials, compiling them, and sending USBs with content to the students who could not access the materials.
3. Strengthening family and community-based support: Through this study we realized that there is a need to strengthen family and community-based support in underdeveloped areas in the global south. It was observed that the experience varied for the students in the remote north and in the city areas. The use of educational hubs for the provision of access to the internet and educational materials can be practiced by other institutions where the students have different levels of access to the internet, and therefore educational activities.

Through this study, it was clear that married women juggled multiple responsibilities while studying from home and highlighted the necessity for having support and infrastructure at home, especially when education is remote. University can look into partnerships with local NGOs and civil society organizations to sensitize the families where women are pursuing higher education to help with emotional support, division of household responsibilities, and availability of infrastructure such as laptop, internet connections, etc. Such a holistic educational model would be necessary to help women thrive in education arenas.

## Limitations

As the entire study was conducted during the pandemic, using remote and digital means for data collection and analysis may be considered a limitation. However, it gave valuable insight to the research team into alternative means of conducting research. The study covered only the Post-RN BScN programme stakeholders and no other undergraduate programs, such as 4-year baccalaureate nursing and midwifery programs, which was a limitation. Focusing on one of the undergraduate programs, the sample size was considered adequate as the data saturation was achieved and no new codes emerged.

## Conclusions

The implementation of online teaching and learning methods in response to the COVID-19 pandemic has had a beneficial effect on nursing education at universities like Aga Khan University. The swift transition to an online modality presented challenges for both students and teaching staff including establishing a reliable and efficient technological infrastructure and communication channels, ensuring a high-quality online learning experience, implementing appropriate evaluation procedures, and managing the mental and emotional stress associated with the pandemic. Additional research is required to investigate the readiness and viability of implementing online instructional methods in nursing colleges with low resources in Pakistan and how students’ experiences vary and needs differentiated support given their resourcefulness and single or married status. It is imperative to prioritize a student-centric strategy to effectively address the professional and personal requirements of students. In addition, it is crucial to share success stories from both local and worldwide partners within higher education forums and policy circles to address the disparity in the quality of nursing education among various schools. The ongoing professional development of faculty members plays a significant role in the development of competency in online teaching. The study proposed strategies within the instructional and administrative infrastructure, that can effectively prepare institutions to respond to unforeseen circumstances in the future and enhance resilience.

## Supporting information

S1 File (DOCX)

S2 File (DOCX)

S3 File (DOCX)

S4 File (DOCX)

S5 File (DOCX)

S6 File (DOCX)
