# Supplementary material for: Beyond the classroom walls: Stakeholder experiences with remote instruction in Post RN baccalaureate nursing program during the COVID-19 pandemic: A qualitative inquiry
Source: PLoS One. 2024 Apr 4;19(4):e0300007. doi: 10.1371/journal.pone.0300007 (PMC10994296; doi:10.1371/journal.pone.0300007)
Supplement: S1 File — (DOCX) [file pone.0300007.s001.docx]

**Mega Transcript for Students FGD**

| **Sr #** | **Question**  **(From the interview guide*)** | **Participants responses** |
| --- | --- | --- |
| **FGD 1** | **What are your views about online learning?** | **FGD1**  **P1** Ma'am Online learning is 50 % correct and 50 % even not correct. As we observed mainly on online learning was this that we do not stay focused on study. Like there is no serious presence in the studies. Like if we talk face to face then in that face-to-face interaction the clarity with the teacher stays correct. Plus, if we talk about the group discussions then in group discussions, we can get things cleared and understood in the face-to-face discussion. On-line that is not possible, and things are much compromised on-line. We learn quite a lot but if we compare it with face-to-face then according to me, it is not that fruitful online. Face to face is much fruitful as the person stays focused, there is the presence of seriousness. So, the students also stay focused.  **P2** Ma'am, I am Nousheen, a year 1 student. PostRN. Ma'am, I agree with Nazneen, that we do not stay focused at all in learning. If I say that I am a mother and I have come to study after 10 years. Then if a mother sits to study at home it is not just enough that you have taken a laptop for study. There are numerous things required to be done and asked, sometimes we are even doing our lectures while cooking food as well. As when the teacher has time then she will teach at that time. So, it becomes so difficult sometimes that we miss somethings in our studies. Like when we are studying with so much connection, then in that there is not so much connection established there with the Miss. And yes, if we consider the skills of the computer then yes, this learning has been very good that we have got many commands on computer skills.  But if we talk about books or some article or some research work, then the research work had not been as effective and good as because as when we used to open any document then they used to ask for significance that you are not authorized for this or you cannot be allowed to read this article. That there shall be charges for it or something. So, much of our learning was being hindered over there. Like from where we should pick the articles. Like how we can read from books if we needed some research material, so with very limited resources we had to prepare for our presentation. And when there were group discussions the people were not able to get connectivity. We do not have such a good system of connection that we can work well on the network. The connection often drops during the class. Even if we are having a paper then the connection drops in the middle all the time. And then the anxiety level rises so much during the papers that once we are given graded assignments and things and then in between that these things are happening. And those nurses who are doing a job and as we are available, and she is doing a job then her time and our time gets much clashed. When we used to be in the school face to face then at the same time and the same location, we used to finish our work and after coming home we used to be relaxed and used to see to our home chores and things. This was 24 hours study time for us, and we felt that we were not being relieved from it. We used to get a call from somewhere if you are free then come for this work meaning it was a full day job, but the marks were given the same like there was no division for the marks. The teachers tough gave good co-operation, in explaining and such to us. but still, the things we were accomplishing on the face to face interaction had become very much difficult. At least for me, it had been very difficult. But yes, it had been a challenge.  **P3** Ma'am there is not any new point, mine too are almost the same concepts, my ideas are also these. But just that online the concentration of the student was not that much as compared to face-to-face. Because there you used to get your concentration done. And then there used to be quite a lot of interaction in face-to-face.  Because then and there you could understand most of the things and ask. In online, the issue was that if you had some other work to do and you have started the class and started doing some other work then your concentration is not done there as what the teacher is teaching you. During that time you could not understand anything as compared to face-to-face when then and there you could understand all the things.  **P4** Ma'am. There were many issues as such in online classes. Because of the net -connectivity.  As all have discussed. Then another main issue was this that mostly it happened in the classes that there could not be any recording happening of the zoom. So, we could not review it later on. In face-to-face, we used to be in class all the time and if we did not have our concept clear on anything then we could approach and ask the faculty after the class. As to anything which we could not understand and all such things. So, this was one main thing. And when the work used to be given we could sit in a group and get our work done then and there. And even when we used to meet the next day we could utilize the time in a better way. In online it happens that all are at their homes, so the zoom meeting is arranged and if some are available or not. That needed to be seen. so, that had been much difficult for us. With regards to the online classes. Rest in online we got so expert, that when we have to do any work online then we can do it easily.  **P5** Thank you ma'am for giving me the chance also. Here my views are not as my colleagues gave. Because in the remote areas, like the northern areas those students who lived there had the issue of the network connectivity. This was not my issue, and it was not my issue either that I was doing part-timers duty so I was not getting enough time and neither did I have this issue that I had children in my home or there is anyone for whom I needed to take care. So, I did not have all those issues. So, I had found online learning much fruitful. The students of other universities I found to be much worried. As our classes have closed and when they will reopen that is not known. For me, I felt that the studies kept continued was an extremely excellent thing, for our university. And we were much encouraged by the faculty. As the environment is blended, as I mean when we had come to AKU before this we had encountered but the full blended course. And this course of ours had blended 100 %. Synchronous and Asynchronous were both being conducting side by side. So, we were to some extent aware of them but here we got more polished. There were even some students in our class who could not speak in the class. They used to be very shy. I have seen much improvement of theirs, in this zoom. As if they do not want to open their videos, we could not see their body gestures but there was a flow on their part. The thing which I found as bad was the group activities. Like when the group used to be made then there used to be quite many of those people who used to be busy in works of the home. Like as motherhood. Or there were some such girls who were also on job. Then when their class used to be with them, we had to bother about their timings too. Rest it was all amazing. I found it very good. |
| **FGD 2** |  |  |
| **FGD 1** | **What are your experiences of online learning during the pandemic?**  Probes  Affected your life (routine, rest, freedom, feasibility, new set of learning) in any way. If yes  how? If no why? | **FGD1**  **P1** It has been my experience, as I was in Chitral and out of Karachi. The main issue which I had to face was that internet activity was unavailable to me. The major resource issue which I had to handle was that I did not have internet availability at my home, and even in the whole area, I had no access to even on 2G on my data. Although the work was going on with PTCL, things were not happening well. Second, some things had been arranged by AKU people but from where they had arranged the WIFI connection, we were already using it. It had been arranged from the same government school.  Second, it was like this that approaching it was out of range because there had been an issue of transportation from there too. As I was using my vehicle, so it was not such an issue for me. But still, that place was such that it was aside at one place around 8 kilometers away. I had to travel 8 kilometers to reach there. And there was a thing that when I managed to reach there, I used to be tired. As there are uneven roads and on which the potential a person has of one thing, the learning potentials which one has. So those potentials of mine used to drop. And the things which I needed to pick was not possible for me, that I keep myself focused on further things. This was one, rest for online the thing, which was good, as I had mentioned that there are some good and bad things of online learning. In this online, there was one thing that when we talk of cultural learning, face-to-face learning. In which although we are in the class. But online learning gave us this opportunity that we could acquire through a transition, which is modern learning too. As all worldwide are coming towards online sessions. So, in this way it is a good thing for us. I would not say it as bad. Although it has numerous issues in it, as you need to access lots of things. As they said you cannot search for an article. So, these things were also happening. Like FHS we were also accessing our library, so we were getting some things through it. It was not that much an issue. But the total issue which was faced was of travel and connectivity. Because I have lived in Chitral, so mostly in the remote areas these are the major things. Then if we come to the face-to-face interaction then in face-to-face when we come into the school even, in that a direct contact happens with the faculty. Whereas online we do not come in direct contact with the faculty. So, then sometimes your concentration gets lost and then the class is bunked too. To be very honest, the mic is turned on and you move from there. You go and do something else and attendance is done. In such things, the studies are hindered. When you are face-to-face you cannot bunk the class. You are physically present. And the concerned things are seen before you. And second, your professional, your physical grooming are also covered in face-to-face contact. As for how you are coming, as to how the style of your dressing is done. As for how you stand and sit. Then in this way in online these things are hindered. Which helps out much in the normal professional life.  **P2** I also have almost similar views. During online classes, the issues were network issues. If electricity load-shedding was done then the internet used to stop and as a result, quite many concepts were missed. And when we used to rejoin and we had missed many major concepts then we found it difficult in understanding them. And what is done throughout the evening was undecipherable to us. One was this issue, but there was this that the faculty gave much support, when we used to tell them the issue then they used to give us a short repeat again, but sometimes, as it was not possible all the time. Because if the electricity keeps going continuously in every hour. Then their whole day would be spent on giving the repeats. Right. So, we used to miss many major concepts in that, and then we needed to take out separate time and had to learn it from some student. That was then an issue too. Second that I too am a mother, so I used to find it much difficult with the baby. Like the baby used to take the whole class along with me, and I could not listen properly either, she used to pull out one earphone from my ears and asked as what my teacher is talking about. And she used to want to listen to her too. So, in that, I used to find much disturbance. I could not focus on what the Miss had taught. Then another thing which was very important was that we could not access books from the library. At the campus, we used to take many books and used quite many different things. Now sitting at home, we cannot take access to the library. We have to just rely on and focus on Google. Everything was just taken or picked from Google. But then there were some benefits of it too that we did not need to do any traveling and we could easily stay at home and with our accordance, we could study in peace. And then there was a style in modern learning.  We explored a lot of things and then computer skills were gained. That we could work on Word and could make our assignments on different documents. So, in that our skill got improved quite a lot like making graphs and charts. We learned many things. Other than that, my baby used to be in front of my eyes so I didn't use to have any tension for her otherwise I used to have to phone my mother and ask about her well-being frequently. So, my tension in that regard had lessened a bit. She used to disturb me during the classes but still my tension regarding her was lessened and I used to look after her too.  Then there were some classes which were such that the teachers used to upload them and we used to then access them in our own time, for me it used to be often that I could see the uploaded lectures at midnight when my baby used to be asleep and I could watch those in peace. Whereas in class the teacher needed to be heard then and there as they did not use to upload the lecture, so that too was an issue. In online we could access the things in our own time too. That had been a benefit too.  Meaning there were benefits too and then there were issues also. But during Covid Online was good and we could finish Post RN timely that was very good.  **P3** Thank you for giving me chance. For me, online I am utilizing my whole time and I am a full-time student. But I honestly think that it has not affected my personal life. Or my house life. I am a mother of two as well But it has given me a chance to manage my time as I was doing in the face-to-face classes. I had the whole night to study and the facilities which were online like recordings and everything, really helped us and I didn't feel any problem throughout. And I think it has not affected my personal life, that much.  **P4** Ma'am during Covid Pandemic we had faced many issues because when I had gone to Gilgit, as due to the pandemic we were given holidays from here and sent to Gilgit. There we had faced quite many issues because there had been lockdown over there too. And then at the time of the classes, to attain an approach towards the net. And then to attain the approach we had to do the traveling. Because the place where I was, had been much far from the central Gilgit. So because of it, we were compromising on many things. But it had been managed, from the support of the teachers and the faculty. And it did not stop it happened. But it had been done along with issues. After compromising with many things. During the Pandemic there had been issues in the family too. As there had been attacks in the family also. There had been Covid in the family, then I had to give them time too. I had to take care of them too during the time of my studies. So these had been the issues which had been with me there in Gilgit due to Covid. Covid had happened in the family, plus there had been studies done too and such studies for which I had to go far, as Sardar mentioned previously.  I would also say thanks to the faculty. As the faculty had given much support at that time as a result of which we have graduated now. Even during the time of the Pandemic. And AKU had also given much support to us at that time. They provided us USBs, and then in those USBs, they gave us all the PPTs, details of the courses and the data ‘and as to how to do an assessment, and all these things, so that is why it was possible. Otherwise, if this support had not been with us then it would have been impossible to happen. Quite hard to complete. For us people from remote areas. Because as there are a net issue and WIFI issue. Then as it is a remote area, we had issues with assignments. Online. And we had to travel and use financial support to go. We got much support from AKU and the faculty. Thankful to them.  **P5** Yes, I found these online classes helpful for me because whenever in the online classes some such work was done as personal then we had to just send a message or an e-mail to the faculty. And the class which we missed, if we wanted that they share the contents of it or repeat it for us, personally then they used to help us a lot. So, that is why the online classes were good and there had been no issue with the connectivity as much. My net, WIFI device, and my data, all were working very well. So, my experience in the online class was very good. And I was able to manage all things very well. I used to give time in class, I never got absent. And if anytime one or half-hour class was missed any time the faculty used to help out a lot.  **P6** Thank you, Ma'am, actually I had to face many issues in the online classes. My life was much disturbed. And my studies used to be disturbed also because my concentration towards studies used to break a lot, frequently. As I am the only one in my In-laws. And I have a mother-in-law. My husband used to be away, and I had to manage the home and to see the maid. And it was a coincidence or what that my mother-in-law got ill, she had fallen and had a fracture and in she is ill in routine too. And in having me at home she used to get support. And she used to call me frequently to get something done for her. I used to get up and listen to her request. If the class were not online and I had to go to the class face to face, then things would have been different. My concentration used to break a lot. Because when the break used to happen then to get back on track was very difficult for me to cope. When there used to be a gap then I would not know what the next point was, and I used to get completely blank. So, because of this, many things had messed up. But it was a relief that the second semester finished.  **P7** Yes, but it was this that during the Pandemic there had been much effect on the studies. Because there are multiple issues in the family considering the finances, the Pandemic. And all. It was a mental torture. That as the routine work which the family was doing as going on outing or going anywhere. Like going to the Jamat Khana or somewhere. All that was being done was stopped all very suddenly and if there were 11 people living in one house then they were all united at the house collectively. Everyone’s timing and all. Then management skills were taught well to us by AKU. That yes, as in which way we manage the time. Our family, our study, and our things. And our faculty had given support to us in everything. Whatever it was, be it that we were going through a phase mentally, like as the last week my father had become very ill and it was my final, it was my paper of Bio-Stat. And that same day I had him admitted to AKU. So, it had been very hectic kind of paper for me. I had been thinking as to whether I give it or not. But my Miss told me to give it. Like as to give as much as I possibly could complete. And as how much I could attempt easily. So that it be known as where I stand. Although I had practice of it beforehand. I had preparation of it. I had passed and my Miss had given a very good input. Then my one assignment of NMP is still left out. They gave me time that as your father is still admitted.  So, I was given time for it. It was not this that they had left us. That I need to submit it then and there. And give the assignments. They had been much flexible in the things. So, with that it happened that the situation of the Pandemic were handled, we kept care of our family too and we also gave our studies on time too. |
| **FGD 2** |  | P1 Miss , one thing which I just want to add here is that almost everything which we have discussed here was true because using IT and the Apps was new for everyone. And everyone tried their best to do something with it and there I think some credit should go to some teachers. We can further say to almost all teachers as every teacher tried their best to make us understand best and also there were some teachers who I would personally appreciate. That they helped on the WhatsApp groups even after the class. As if there had been any queries of the students, they tried to resolve them. It was difficult for us and the teachers too. But due to this sudden situation, it had been a first-hand experience for all. For us and the teachers. And overall, it was good and if we see to the grading then that too was not much bad either. It had been a good experience. But if we have to continue it in the future then I think it would be a little difficult.  P2 if there are some advantages for some people, then it is not possible that there is an advantage for all. And as one of my colleagues who mentioned here about the group work and that the group work of 5 to 6 people had to be done by one person. These kinds of things even happen when we are working in the group when we have face-to-face classes, I think that is not relevant to the question which you asked. Yes, but there is one thing to which I do agree that this used to happen even when we sometimes had to do group work. Because of the Pandemic situation, some people are sick, and their parents are not well. And there are these kinds of things happening and they cannot blame anyone if they are not participating in these online classes, everyone wants to do their best. For personal reasons and if somebody has some problem then there should be some leverage and these sorts of things should be considered.  P3 Actually, I wanted to quote something but in this, I do not want to disgrace any teacher or anyone but one of my experiences which I felt was that when a connectivity issue or any other such sort of problem is faced by a teacher is considered as acceptable. But if this same sort of problem is happening to any of the students is considered as a lame excuse so there should be some sort of elasticity in this manner too because everyone was stressed out, everyone was having different challenges being in this Pandemic. So, there was some sort of lacking in the flexibility towards students from the teachers. So, if we are going to continue the upcoming semester in this way, there should then be some sort of a free hand for these kinds of things too. |
| **FGD 1** | **In your view, what are the advantages of online learning in pandemic situation** | **P1** Yes Ma'am, thank you so much. As we spoke before about the advantages, that much of our online computer skills had been polished, meaning these had improved. And second our habit of learning as compared to traditional learning then considering that if I evaluate myself, then in traditional learning when I used to go in the class, I used to attend the class and had my clarification completed and then finished. Then and there I used to sit and get all things done and then I used to somewhere here and there. By online classes I had this change in me that I had to do much study. I was much connected with my studies.  **P2** The experience for me was much knowledgeable because I had become completely focused towards my studies. The main issue was just that of internet connectivity. And that had made it quite difficult for me to do the things. So, then it was this that whenever I got an opportunity I used to, It was one that it was me and Sultan in Chitral. We were two people and he has not participated in this discussion if he would have been here then he would have told a lot of things too. It happened one day that there had been an evaluation and the net facility at Chashma had been stopped due to some reason at the tunnel site from snow. And the net had discontinued. So, I had gone to Garam Chashma in the morning and from there I had to go for my paper at 12 o'clock. I had to travel 50 kilometers towards Chitral. 6 kilometers to Garam Chashma and from Garam Chashma 45 kilometer further on motorcycle I had to come to Chitral. So, all these things had been very difficult for me. Then with this when I got there then what I had to do was that I had to cover several things simultaneously. One was to arrange for a room and all these things. So, it was this that the things which I had studied priorly. The rapport that had been built of studying that had been very good. Because I had gotten things cleared in advance. One had been this that as there was an issue of net then anything can happen anytime. So, I had been prepared prior for my exams. I had done all the things in advance. Unfortunately, the USB which had been sent to us, My USB had been empty too. So, I had not got anything from the USB. So, then I had gotten to give the exam by getting it accessed from the internet. The advantage in that was this that there had been a transition on the pattern of international level. And we gave the exam and got graduation on time. And our access on the internet and the computer skills have developed well. Those have been enhanced much. And our knowledge has enhanced too. and due to that it was such that the other people who had been there we were also teaching them related to our nursing. And that had been good too. It is out of the topic but the nurses who had been working there whenever they needed our help, then we used to go and do these things with them.  **P3** Thank you Ma'am. In online learning what happened was that we had got to learn a lot. Because we used to think of Plan A and if that was not done then we used to go to plan B. We used to think of ways as how to solve it. That had been a very big advantage for us. Then second our computer skill had improved very much. As how to enter in Zoom, or as how to use a software. And as how to do online learning. That had been improved a lot. Then we adapted to professional learning skills. As how to try ourselves that is what we had got to learn much. Then we learnt as how to do individual learning. And as how to explore the resources. And as how to solve them. This we learnt well while learning online. As in plan B as how to solve, as how to learn. A manner of professional learning was gained as how to approach the resources. And if the faculty is not available then how to study ourselves. And if the Zoom link is dropped then how we have to utilize the online learning. Of those resources. So, we learnt a lot.  **P4** Okay, I agree to Nazi and Sardar for the advantage which they mentioned.  **P5** Ma'am there is this other benefit in online classes that we did not have to bear the printing cost. And when we had to submit any assignment, we used to type that online and we used to send it through mail or used to upload it on VLE. Otherwise we had to bear much cost on the printing if we had to give hand assignments. Second this that online we have learnt to make zoom link. Then we had learnt to make Quizzes. And the data collection was also learnt then as how to analyze the excel sheet. Actually, I had done a diploma in IT, but I had totally forgotten it. But during these three to four months of online, I remembered all that which I had learnt five to six years ago, and I had forgotten. So, this was very well. |
| **FGD 2** |  | **P1** In the advantages, I think the main thing was getting up early and going to the campus within a short period. Whereas online we are comfortably sitting on the bed and without a change of clothes or anything we take our class. These kinds of benefits were faced by us.  **P2** Remote   - - Low cost when you have internet accessibility   - Did not lose out the academic year   **P3** Karachi   - - Save travelling and physical presence   - Limit of two years to graduate   - Hostel dweller said, for me , coping up with study is easy when I am in my home city and not in karachi, hence, online learning was a bliss, particularly in the COVID times and I didn’t have to be alone in the karachi city.   **P4** Indeterminate   - - it is good if we have good internet connectivity, for me that was new experience and i found it interactive although initially that was challenging like concentrating on content or lecture   - Improved technical skills |
| **FGD 1** | **In your view, what are the disadvantages of online learning in pandemic situation?**  Probes  Changes in daily routine (time consuming, fatigue, balance with work, and family responsibilities etc.) | **P1** For the disadvantage I would like to just bring up this point that I felt that this semester we had emails at any time. That there was no time. No timings at all, from 8 to 5 the studies are being done. Whoever and whenever they want, they are sending emails at 2 o'clock in the night. The things are being uploaded, and that was a little bit disturbing. This is what I think. And secondly in online classes like we didn't have like proper break timings. Or class end time. So, if we have one hour break the classes are going on till 1:30. So at 2 o'clock again we have another class. So, the house mother for me and my other colleagues we had only one hour to cook for our children and for us to do our work.  So that was one thing that really was disturbing for me. Like we are not having a tea-break and without tea we were not able to understand anything. So, break timings and one-hour break should be, you know, on timing. And emails and other assignments should be like in our school timings. If we are already giving our time from 8:30 that is the time our classes start and till 4:30. So, things should come up in that particular timing. Yeah sometimes it is okay that we do get emails after that timing and if we as a student are acquiring anything on emails on weekends and late nights. So, we should also get the replies at that time also as if the faculty is expecting us to do the work that time so the students should also get replies on weekends and on the late hours.  **Probing Question: Thank you, so you mean to say that online learning is time consuming. And it should be...**  **P1** No, it is not time consuming. It should be in the timings which we already have from 8:30 to 4:30 or 5:00. So, it should be with-in. And then we have our extra learning, so sometimes, what is happening in online learning when the groups are made as there are WhatsApp groups then anytime you can get anything. That this assignment has been uploaded, this or that had been done. So, it is a little disturbing. That you have to leave all things at home and then you are, you know, doing this all.  **P2** Ma'am one issue was this that one there was not a proper, as Mehnaz said, the midnight issue. And it was that we used to keep away past midnight to do our assignment.  Whereas in face-to-face classes when we used to get off in the evening then we used to keep one or one and a half hour of grace time to reach home. So, before that we used to complete the assignment of that day. So, in the previous semesters we had this routine that we used to get our assignment completed over there and then we used to do our home chores after coming home. In this we had to complete all the assignments by staying up all night. So, after that when the course ended, I used to say that I would sleep the whole week, when all of it would complete. Because from all these many months we have been stay awake the whole nights to study.  **P3** Hello, good evening to all. The main disadvantage that had been was this that like there had been a course of ours that was of Mental Health Nursing. Although we had covered it very well, but the practical or clinical based kind of area or the learning of it was hindered. Like we were intervening with the patients online, but like the activities which we were planning to do with them, then that thing was not being done face-to-face. And like as we had wanted to do it with a potential then we were unable to have it done it that way, online.  So, I think that this thing as our clinical base and our practice and our assessment section was incomplete otherwise, I do not find any other disadvantage in the online learning. Except this.  **P4** One disadvantage which was of the online classes was that there were only our connectivity issues. Then apart from that another thing of ours which we can call a disadvantage was this that we were unable to know whether we were all at one platform or not. Like as all were connected but then all used to go silent. You cannot see the body gestures of anyone. And then especially when we had come on the Zoom meeting and MT for the first time then we were unable to operate it. We used to get calls and we did not know as how to put the video on or as how to answer in the chat-box. So, those things we could not manage. And sometimes there used to be a misconception that she had connected and gone somewhere and that there is no student. So, these things had been faced in the start, but later on we got used to these things.  **P5** Yes, Ma'am professionalism has not been kept maintained as Sardar mentioned previously that when we go into the class, we go there in a professional way and we take classes in a professional manner. We are serious during the classes. And in a professional way so that was not maintained. Like as now in Zoom, we open Zoom and then have the mic on and if there are any sounds from the home like as any other conversation is heard on the mic. So, in that the professionalism cannot be maintained. Like as sounds in between and voices, voices of children. Plus, if someone is taking a class on the way then the sounds of the traffic. There is disturbance and such issues. Then professionalism was not maintained in the class. Plus, in group working when we used to be working in face-to-face then we used to give proper time. We used to have a same time, as in this time we shall sit in a group and in this time, we shall be doing these works and would clear these objectives. in online it used to be such that if there was a mother of children then their time used to be separate and those who were unmarried, they got separate time.  So, in this way there used to be clashes in the middle. Like as when the other would be free or when they would give time, these were issues and objectives completion was not done on time. There used to be clashes in the middle whereas in face-to-face it is not so. In face-to-face you can be calm and cool and do the discussions with each other together with different planning’s. Like as we used to do group planning and group discussions previously. Or the group work. Plus in group work of face-to-face we had this opportunity that we used to go to the teacher and take their opinions, whereas in Zoom it was such that we had to ask for the timings of the teacher or the timings of the students then we had to make the zoom link and then finally we used to get to share the things. So, that was much time consuming. So, that is why I did not like it.  **P6** Thank you Ma'am, there was one main thing that as I told before that over there we not having the internet connectivity and then if we somehow manage to arrange the internet through some resource far away from our houses then the main issue in that was this that we had to also arrange some accommodation, which was a main issue. Because by having the internet accessibility we had to travel 60 kilometers and in that one was this that we used to get tired and second extra fuel was consumed for the travel and then we also had to eat from outside. So, then our exposure was increased. There was much risk of exposure of Covid. This had been our main disadvantage.  **P7** I think the questions are somehow interrelated so we can move up and down. So, I think one of the points which some of my colleagues have missed maybe, but it was not a challenge, but screen time was really too much. Sitting from 8:30 specially in our MHN Clinical which was Mental health Nursing, then from 8:30 to straight till 5:30 we were on screen and then on a daily basis we had to do different kinds of tasks to do for the other. We can keep in the mind that the screen time was really difficult. And people like me, I used to have two Panadols daily for my headache. Daily. And this was discussed in our class as some of my colleagues as well. So, I think this should be kept in mind for when you are planning. Like because we are taking classes online and then we have our assignments and then we have our studies. When we are in face-to-face, you know, we are in AKU and we are taking classes, then we come back home and then we start our computer and we study. But on online classes throughout the time we are studying on the laptop, so this should be kept in mind or the classes should be minimized, or something should be done and assignments for the next day should be not be given. That if today there is a class, then tomorrow again there is a clinical and then post conference is to be done too and Pre Conference also and then it needs to be done the day after. So, this needs to be seen to a bit. I think. That the screen time should be less.  Even the children used to say that Mama You tell us not to do much screen time, but you are the one who is sitting in front of the screen full time. When this is completed then your WhatsApp group is ongoing. So, this should be seen to a bit. |
| **FGD 2** |  | **P1** I think there were more disadvantages. Rather than the advantages. Because when we go to the class, we know we have to go, and we have it in our mind and we prepare for everything. Because what I would like to mention here mostly, is that as I have children, family because of the Pandemic the children were also at home and due to that reason, we had to give some time to them too. Even though we tried to keep them calm they did need attention too. So, we could not be able to work in such a way as we needed to concentrate and focus.  Another thing which I would like to mention here is that some teachers did not turn on their cameras. I feel that the teacher should turn on their camera because those teachers who turned on their cameras, I felt the interaction with them better as compared to the teachers who did not turn on their cameras. Like it was just listening and nothing else so even if we tried our best we felt as if anything was happening. And especially because of the home environment for me I think it had more disadvantages instead of advantages in studies. As when we come here, we need to learn something, we want to be something, and we want to achieve something. There were many challenges and many targets to see. But when the online classes started, the passion and the energy and those things I felt were getting not dim but it was like that we were not getting such level of study which we needed to maintain. It could because of us or maybe it could be because of the environment. So that is why I do not see much advantage here. Thank you.   1. Remote Areas  - Difficult to make up for the online classes when there is no internet accessibility - More stressed due to limited accessibility to online class, less stressed about the core content of the classes - Transportation costs (in thousands) and then seek help from someone for a ride to the spot where internet was accessible  1. Karachi  - In Online classes, you have to wait for the turn and the topic goes off the way - You cant ask questions when you feel like - Facial gestures are missing in online classes - Economic issues – transport expense, technological resources, internet bandwidth - Far away from family, facing depression - Home is not a conducive environment to study, because I have kids and household responsibilities to look after (2)  1. Indeterminate    - Too much screen time – sitting for 4 to 5 class have been difficult during online classes.    - Cameras were off due to low bandwidth issue, because we are used to teachers’ physical presence. We perceived that the physical presence would help build clarity in the face to face environment. Particularly teachers should turn on the camera (n=2)    - If Microsoft Team is not working properly, or an individual was not getting the conceptual clarity then faculty would move on or speed up the explanation because of limited time in synchronous classes. Teachers in all classes would not take care of individual needs, if I am not understanding a particular concept. (n=3). I would still prefer face to face classes. Particularly peers were sometimes rude and degrade the ones who don’t get the concept in the class and those who ask questions during synchronous class. peers usually say, don’t waste our time and ask the teacher, later on (this is online class issue, more than face-to-face).    - Cyber patient (a software) was very challenging to operate initially. Initially it was difficult to learn, and then it got easy.    - I also had to buy laptop so initially it was a bit difficult until i became familiar and comfortable using all these gadgets    - Too much connection with peers and no study- life balance    - Affected my study pattern and halted my capacity to go to library and study post-class sessions    - Concentration was difficult –    - yes, group work and time management was creating such unpleasant conflict in opinions |
| **FGD 1** | **Do you think online teaching is an effective learning strategy during pandemic crisis: if**  **yes how and if no why?** |  |
| **FGD 2** |  | **P1** Yes, I think that online learning is a very good and effective strategy during this Pandemic because due to that strategy we were able to finish all our courses on time and second important thing is that we have a vast variety of different modes of strategies, for example, we have some videos. We have received articles and podcasts and we got the chance for online clinical. We were even able to complete not only our theory part, but we were able to complete our clinical part as well, during the Pandemic. However some of the clinical expertise was able to manage in some of the courses whereas if it were for the health assessment or it was for any other courses which needed very deep clinical expertise, so for that, I think that online clinical wouldn't be a very good decision for the upcoming semester. Because some of the nursing skills need the supervision of the faculty, needs some experiences, needs some critical part. So, we were not able to think critically and see the things from that clinical perspective and an online clinical, but yes on the theoretical part online will be a very good strategy for study.  **P2** No, Miss . As Sanam has told all completely. I agree with Sanam. Mostly our Nursing field is practical. Our focus should be more practical as compared to theory. So, unless we do not get any face to face practice, our skills would not improve. So, I would highly recommend that our skill learning and performances should be done face to face. I agree with Sanam. Thank you.  **P3**  Yes, most of the things that I wanted to share Sanam has covered it, but the two things which I want to highlight are being a nurse and having an advanced learning degree in my typical field, I need to remain in touch with my patients. So as per my clinical point of view, I would say that to totally remain on the online study is not a good means of learning. As far as the theoretical concepts are concerned, I would say that being in the present era and being in the search of new means to discover how we can cover up the major things with minimum challenges. I would say that online study was truly helpful and no doubt about that, as it has enhanced my IT skills too. Now I can utilize different apps and multiple gadgets just because of this and so it helped me in that way. Thank You.  **P4** I wanted to add one thing, I do agree if we see in this aspect that to have the online classes in this Pandemic was not less than any blessing. At least even staying at home we were able to get something, and we were able to attend classes and even finish our semester. And we learned many things and one thing was our experience. But even then, in this Pandemic we still can say that it was a blessing, at least we did not have to stop our studies. We were not stuck. And at least our organization and our school gave us an opportunity to continue our studies. I wanted to tell you this. Thank you.    **P5** yes it certainly is |
| **FGD 1** | **What are the challenges that you are facing in online learning?** | **P1** Miss actually the issue here was that when we were giving the assignments, like as the presentations and making of the PPT, so all the material had been gathered but when the PPT was being made then I made one PPT of two slides from my home. Then if Mehnaz has to do work on it, and if you have to do work on it, then it used to be a challenging kind of work that I finish my work and then send to Mehnaz and then Mehnaz further send to you and if you want to add something then you send to Mehnaz and then from Mehnaz it come back again to me, then it used to become a such a long process, one work which we used to do in one hour at one platform altogether. then for that same work we used to spend the whole day. In it the mail used to go from here to there. Either she is busy or that task which needed to be done. Like when sitting together then many minds work together at one time. Here if one person does think of one thing and then to give the justification too that I have done this work over here because of this particular reason and have not added that, so we used to spend WhatsApp time in convincing one another. And we used to give so much energy on that so the energy which we needed to spare for our studies that energy used to be spent on our group discussion. So, these were extra burden and workload. So, the small works we used to do easily like making the PPT. Like in group discussion it was told to us that we need to do a sort of role play. It had been such a tough task for us. As how we show the people as role play. Like we have done this work. The voice is recorded but still at one platform there needed to be a meeting. If even by following the SOPs, we are not getting permission to go to AKU then how should we follow those things and in which way we get to learn it on one platform. So, our tasks should be modified in this way. Like yes, a bit of work is good, that we had got to know about google docx. And we did one presentation only once, so this we learnt too. That was a challenge.  As we were not able to make the PPT and then second this we learnt this that we went on google docs and then all the people can see one PPT simultaneously and then they can place their working at a time. This challenge we discovered in this and second in the video call, during the presentation the teacher had said that video call should be on, one our connection is not done as it is, alright and you know that we took two days and made one PPT ready and we inserted a whole video in it. and we did meetings in Zoom and we checked it out that our platform is correct. And that all our things were correct. We had made all the things ready till 12 midnight. And then when the second day we went there for the presentation and worked with the Miss and did video call too. As there was a video call of six people and there were 42 children already in it. So this much connectivity was not possible as we live in Karachi and even as good a device, I had with me. It was a full charged device and it was my own computer net still then my video got hanged. And the video for which I had spent three days that video could not be played in that presentation. Because as the Miss wanted to keep the video open as she wanted to see what our gestures were like.  Because if we are bringing something new here. To bring it to normal then there is some time required for it. And we need to understand what are our hinders? What are our challenges? We need to understand that we needed to be prepared beforehand and should have checked on Zoom. So, if I am seeing on Zoom then I am watching it under 5 people. That Zoom and when I am presenting it then it is with 42 people in it. So, the connection would be divided within all. So, this issue of connection needs to be resolved. As we are giving these assignments, we do not have any issue we are ready to perform as of course it is learning of our group if we see those things. So, you must open it for five minutes the camera to see if all are present or not. And all are doing it or not. But if you want to have it the whole time in continuity, then for us the effectivity is not applicable. The thing which we did with so much effort and hard work was not visible. And then marks are deducted.  **Probing question:**  **Are there any suggestions to improve this group working or group discussion, group tasks during online or blended learning environment? As how can we improve it, do you have any suggestions with you to make it better?**  **P1** Suggestion is only this, like the minds of all of us people is change, firstly the task given by our teacher should be well explained. Because now we are studying Asynchronous. So, you have read a sentence in English, your way of thinking is different and mine is different, then Mehnaz is different and Nazi has different. Each person gets different perceptions with that one sentence. Alright. So we get five perceptions, if teacher can explain these five perception in one time, as we are not explained the task, we are told that this is the task, you read it and assume it and then on that you need to make that task.  When we get the queries and we go to the Miss and that is then solved. Sometimes it even happens that we have changed all our work at the last moment. Our assignment for this reason, because as we are not able to meet face-to-face, we have so much to do as we have to do Zoom meetings and all then these are the hinders that we have face. So, suggestion is this that when you are giving us some task and are really trying to explain to us. Then we should be told then and there as what their expectation is, like as a teacher what expectations are kept by her. Then she should also listen to the students as what problems they have to face. Like as what we have to face as a student. Like if sometimes the teacher also thinks that it is a new norm and these have not been with us before, this is the first time that we have had to face such a Pandemic situation in which we cannot meet in a group and all these things so then we keeping all the perceptions the teacher should also have a flexible mold into it that it is alright. If it is our due date and there is some issue in this group, because sometimes such a bad issue happens that of the network issue, but no it was said that it will be closed at 12. Then there the deadline becomes so hard, you have to be prepared ahead of time. So before, we do not know what is happening in whose home. We can only assume as what it can be, if there is one or two-day flexibility given for that particular group is given where we can be flexible that is one thing and then in group discussions there should not be very big groups made. There should not be a group of 10 to 12 people or the group be of 5. The group should be of 3 people. So that we can connect with the 3 people on one platform. Like to connect 6 or 8 people is very challenging and the group leader gets a he adache like as how she needs to connect and get them together at one place. If one has not done a task, then the other raises the issue that he has not done that task.  So, you have not told her anything. So, as a group leader it becomes much challenging. One there should be smaller groups and so that our time and our screen time becomes of minimal usage. Alright? One is this point and the task which we are giving, any task then after uploading that task you should ask the students as what assumption they have gathered from the task or either the teacher herself tell that such is my expectation. Come to the point that this is my expectation and you people should work according to these expectations. So, it can be known. Sometimes we do get the rubrics and points that at such points we are giving justifications on our presentations or quiz. Sometimes those are not given or are given on the last day or even afterwards. So, the challenge then becomes this that we had thought that this would have given only two marks. And the Miss has placed ten marks to assess it. And we have done that work the least. So, if we are given these rubrics beforehand then such things are maintained.  **P2** I would not say no to what Nousheen has said but on some point I would not agree though. All the things we do discuss before aligning which is what I personally think as a full-time student. The point I would like to suggest is that if the group work is that much important that you cannot finish your work of the semester without it, which is not, I guess. If group work needs to be done, then at least when the presentation is being done then at that time the person who is presenting then there should be individual grading at that time. Like if in an assignment one or two beings are doing the whole work whereas the rest of the group is not doing it, which is true, and the teachers know about this. So, at least when you are presenting it then that person should be given the justified marks at that time. For example, if Parveen has completed her whole assignment and she is the one who is doing everything, then when the presentation is being given then she is getting the same marks and I have not done anything, and I am getting full marks too. Then that is not any justification.  One a person is not doing the work and she gets full marks and whatever kind of presentation which she is giving then for that she is getting the same marks and if I have had done extra learning and read extra articles, and I am even showing extra evidence. For example, anyone is showing it. Then that person is getting the same marks then this is no justification. And till whenever we have to keep these online classes as till when we are with the Covid, so I think there can be many other assignments apart from the group work. Which can be on the individual basis. And secondly if the group work needs to be done, and this was also discussed too with the groups, that we be allowed to make our own groups because I know with which people I have an understanding and that those are not part-time students either. Because with part timers, it is that they are mostly doing duties and their timings do not match with our timings. And even here there are some people sitting, who are with me in the session too. That we have worked with them and done practice with them on Zoom when they finished their duties even at 12 or 12:30 at night. Which is not justified for us, who are you know, we have been waiting the whole day for that person as he/she will come home at 12 o'clock at night and then we shall do the practice. So, the groups should be such that if me or Kiran, Kareema or Parveen if we are not doing a job then you make a group of ours. And those who are on duty they then with their own accord make their own group and get their timings set and do their work. So, here there had been much of a challenge. And I think because of the group assignment everyone had with anyone, one or two issues between themselves. And this was known by the faculty, but nothing had been done on this thing. I am very sorry to say that if that thing goes to them then the first attack would be this that this point can only be by Mehnaz.  **P2** No, no that’s okay because I have already discussed this, but nothing was done. And the semester has already ended.  **Probing Question**  **Can you suggest some of those tasks which can be done apart from the group activities?**  **P2** Okay. With a group activity the grading can be done individually, first. Secondly you can increase our individual assignments. And it is not necessary to give the same question to the whole class. If there is one topic then the teachers can, you know, give so many questions. I have a different question and you have a different question and I am sure that there is whole bank. There is a question bank. So, everyone can get a different, if we are 19 in class and everyone is not full time then everyone can get different questions and they can evaluate on that. And they can give article reading. There can be reflection written on it. There are so many things which can be done. I think that group work is total waste of time. Literally it is waste of time. |
| **FGD 2** |  |  |
| **FGD 1** | **What are your experiences of online assessment and grading?** | **P1** I agree with Mehnaz as she discussed each and everything. But one thing I am sharing to be very honest, one thing which I did not like in online classes was that our evaluation, our exams and in that it was such that if during the exams our net used to stop then it used to be all submitted and all those things. That had been a big issue. Then at the exact time of the submission, it once happened to me that as there had been an issue of net. And my time had passed, and I could not submit. I had waited for three hours to have my work submission done and it had been a very big issue for me as I had been in Chitral. And it had been my final and I needed to submit it. These things. And secondly, it was this that when we attend the classes online and then afterward there is an evaluation. Then in that my one recommendation is this that what we had done first as Asynchronous. That should not be done when there is time duration. Then there should be a purpose of study, that when you are judging a person then the aim for it should be this that as how much potentials the student has achieved and how much purposeful it had been. Then the evaluation should be done on that base. Even if I am to fail and I have not gained any knowledge then it is better that I fail rather than pass after cheating. So, this was one thing, we could even do things by asking here and there and then getting passed in our exams. So, this thing I do not find good to any extent.  **Probing Question:**  **By Asynchronous, you mean Asynchronous should not be done because you mean to say that there is not enough accuracy in it? And reliability?**  **P1** Accuracy and authentication are not there. An authentic source does not happen like if I have to submit my paper in 6 hours or 3 days. Then I would share this at ten places that I need these things in the exam. And in that, it is this that there is no preparedness of the student.  As they are not ready for it. Then it happens that people just leave their studies. If I talk about myself then it would be the same with me. Definitely. It would have been the same for me that I had messaged someone that do it for me, as I have this issue. So, to be very honest I too had asked some people one or two questions. That please do it for me. So, this is a thing which is to be very honest, I have told, these things should not be done. Yes, then afterward the Synchronous exams had happened and there had been a time period and we were preparing for it beforehand regularly too.  **P2** So, I agree partially with Sardar. This is not that like everyone does this that you have given me the paper and I will do it. The same goes for our class, I completely agree.  What he is saying is what completely is going on with the exams but if you take this individually. If I talk about myself, I know I can get the paper from all and everyone. If I ask Alina that give me the paper, she will send it to me. But it is up to me, like for example, I would say that Biostatic we had, I mean that if I had asked anyone to give me the paper they will give me, they will not say that no, we will not share. But the point is that I wanted to learn, so I did everything myself. In the end, I just said that please can you tally me the answer. If I am doing it correctly or not. So, it's all individual, it's up to your ethics and you. I also had two hours to do the exam, It's up to me that I open my Google or do my, you know keep up the notes. Though we didn't have any time because we had a limited 1 hour, 20 minutes or I hour 45 minutes. So, some of us, we do not have all the time to open up all the notes. But yes, if you say that there is someone, some people. There must be some people who do this. If you want to learn and if I don't want to learn Bio states, I too would have done copy paste and would have done it. But I know, that in my class, my class fellows, literally they have learnt. And then they have submitted the assignment. This is my opinion. And I am not talking about everyone. But this is done as well.  **P3** Miss , I want to say that I agree a lot with Mehnaz, she was saying correct that it depends on person to person. No doubt I too had quite many issues at my home. But we too had worked hard to prepare our assignments. We had stayed up the nights and managed separate times too. Just in any way we solved it ourselves. And we completed it by being capable our self. Because every person has come to learn, and yes in the Pandemic situation it was such that we could not judge as what was happening in the others home. As who was ill in whose house and what position was prevalent in whose home. And who was facing whatever mental torture. Because if you see the boys, then what is their source of income. And if you see to the girls then the maid is not coming then there were so many other matters to see to.  So, all the work had to be done by them. Alright. Although this that if we have done commitment that we are to study, then it is correct that we have to do it. Then in that it is not our prerogative that we seek out a second way. That we do a cheat with someone. I liked this point that if we were given a duration, then we were doing a consideration on things. That if this question has been given by him, then what is the reason for it, and the answer which I am going to give then what is the reason for that. So, we were doing multiple thinking. And were bringing out the answers on it. And then we used to get one answer as this is the best as this has such reference and its relevant points are these. So, our study was such that we were able to go deeply into our study. I studied very deeply. I am satisfied that what I had done was good.  **P4** Like as Mehnaz had previously told about the group work and we were having the final courses of our nursing research, it was some presentation of quantitative. The main thing in it was that when we had gone from here, we had been totally out of internet. We had zero internet facility. The PTCL net was not working either. So, then the 4 to 5 people who were with me in the group then they had to do the work. Then one day I had got the opportunity and then I had come and included the little bit of content which I could make. I do not think that it would have been so much fruitful for them or that it would have helped them out. Then when the evaluation time came then in the evaluation, I got the same numbers and they too got the same numbers. So, to some extent this was a non-justified kind of thing for them. I damn agree with that thing.  So, then I had some concerns and issues with me too. Because I do not have any internet activity available with me. And the second thing was this that the internet connectivity which had been provided to us by AKU was from Government High School, Garam Chashma. And that had taken a week. Because it had been provided from here to the people. I had given them a better suggestion that there had been a AKBH boys hostel over there in Dolomoot. I had sent them email multiple times that you have us stay over there. One from that our accommodation would have been saved and we would not have to do unnecessary kind of transportation and our risk of exposure would be lessened. And secondly that place had been such that it was in the town area and the WIFI access was there also. So, because of that much thing for us had been hindered and we had to compromise on our learning too. Then if we speak of the online evaluation then as I had said before that in Asynchronous, then the faculty already know that how much potential is within which student. They also knew as which student stands where. Then it is the same thing with each other. The reason for me to hit this was not that I was targeting someone, no it is not so, or that they are doing cheating, I had totally not meant that it was cheating. To pass by cheating as to pass is not a big deal. But we are in such a situation that moving forward we are delivering a care. We shall be dealing with someone’s life. When we would be attaching with someone’s life or providing someone care, then would our care be safe care? Like those things which we do by seeing someone and by just tallying it, then no doubt we would be giving our own of the things. But what we have learnt then would our learning be as much fruitful that how much we could give input in the improvement of some patient.  So, these were all the things, rest about evaluation it was this that most of the things which we were doing had many kinds of issues within them. There were distortions so the things were not being clearly done by us. So, then even in evaluation the online forums which we had in Chitral then I could not access those. It had not been possible for me in the initial phase. Then later on when we had to record a protest. All those students that were there we recorded a protest. Then it must have been received by the faculty and then when we had moved up to the administrative level and spoken about it. Then we had got those things done on will. Then we had even taken our political leaders in the approach too. Then we had done a direct protest. We had taken out a whole rally following the SOPs. We had faced many difficulties in it. Because we had to do something for the change as our studies were being compromised. We would not have graduated on time. And just because of two people the rest of the 30 more people were stuck. So, these were all the things. Then even it was the same issue with the grading that all were given equal numbers, and everyone was given things then the difference which is between each student then that difference is not left there.  Anyone can do something and get the grades but the knowledge which can be built that we cannot transfer to anyone. Then there would not be any knowledgeable thing in that until the grades come high.  **P5** Yes Ma'am. I would talk with regards to the assessment. It would happen such in assessment that in human health we had our exam, there had been around 100 MCQs, I think. And we had to do them within an hour. After opening the question paper, the anxiety level had gone to such a peak that I felt that the time would be less, and the paper would be completed and that my MCQs would be left. Just by thinking that my anxiety level rose to such an extent that I just clicked the MCQs without reading them. My anxiety level was such that it is not that my MCQs be left over and I fail. Because there were 100 MCQs and the timing was less.  Then this discussion we had with our faculty in the feedback too, regarding the timing being less and that with a 100 MCQs. The anxiety had gone to the peak level as when at the time of submission, this even used to happen in Gilgit too. As where we shall find a good internet and where I could go and submit it on time. So, I had run around a lot for the net, because of the assessments. As where do I need to go and do my paper and as how I submit it. So that it can be submitted timely. These were the issue which happened in assessments and every time our anxiety level used to be at peak. That it is not such that any of my assignment be left and I also be left out. Especially when we came here, and this paper of mental health had been done. The one which needed to be done in one hour. In that my anxiety level had been at the peak. Then in that anxiety I had been doing wrong answers in those MCQs just to get it done on time. So, I did not like that about online.  **P6** For this assessment I would suggest that sometime be given. Well this was a situation of Covid. So, in Covid there should be such a thing that there be some extended time for the students. As the thinking of the teacher is different and the thinking for us takes a bit time. The teacher thinks fast because she is prepared beforehand. We are just now learning and taking it all in. So, our thinking time period and the clicking of the MCQs that too took quite some time. To read the scenario and then to bring it to the mind then with our previous knowledge which we have studied, to prepare and select the required MCQ was time consuming. We could not click it suddenly. So, what I did was that out of fear, whatever seemed to come in front of me I kept clicking that. So that was a bad experience as an assessment. And due to that my marks could come less too. Thanks that I passed, but the marks could have come less or there could have been an effect on my GPA. So, the time level should be increased a bit to the level of the student.  **P7** Miss, if I speak about the assessment and online grading then in the previous semester it had been also such that we were using LRC, so for me then it had not been an anxiety level. That is how I would attempt my paper? Because this kind of attempt we have done before. In our midterms and our finals. So, for us here was this good point that our faculty was supporting us very much even they were telling us that we shall include such, such and such modules in it. And we shall not give all MCQs, we shall be giving you SAQs too. Short answer questions in which you shall have gaining points for yourself. They were assuring us continuously and the third thing which we all used to fear was the issue of our connectivity. There was an IT team already available with us. We also had their personal contact numbers shared with us. So that during the exam if we have any kind of trouble then we could call the faculty or call the IT person even. But we were also told, even though we had not known it initially that there would be a time period. And with the passage of time, we had been aware of everything.  So, they had made us backup plans that even before the exams they had filled evaluation forms from AKU in which it was asked as what the effectiveness is of the devices which we use. As if we have smart phones with us or not? Or if we have WIFI connectivity or not? And that what things we have with us as backups. And after doing all this we had then had our assessments started. So, we did not have any anxiety level with the assessment. Especially I did not have any. and we had some such papers that lasted for three hours. And we had grown bored with them as who will give such a long paper. So, it was like that too that we had requested the teacher to increase the time. So, we had even done a paper of three hours then in that it happens that a person gets bored. Like why the paper is so long. So, if the paper is of less duration then we get worried as why it is so short. So, we have been exposed to this beforehand so as related to assessment our anxiety level was not that much.  **P8** The thing which had been discussed about the MHN paper, I agree with that the 100 MCQs were too much and there was very less time. Even there were 20 to 25 MCQs that which I had ticked without reading. Maybe that some of them be correct. 20 to 25 is a very huge number of questions, and those I had done just on a fluke that there had been just some minutes left and my paper was just about to end. So, I just ticked without reading the questions, just assuming that some would be correct. So, this was an issue, and second miss there was much tension of connectivity, so we tried to finish the paper very fast and quickly.  So that the electricity does not go or either the net does not go. So that is a very limited time period. I also want to discuss a good thing that it was one English paper in the semester before the last semester when the Covid had started. In that semester there had been a very good paper of English in which it was such that the Miss had given 24 hours but in that she had given time of 1-and-a-half-hour paper time which we could do anytime according to ourselves. This was very good thing as I knew that at such time my light would come and then after that I had a limited time to do the paper around 1and a half hour, but it was such that we could do it according to ourselves within the given time of 1 and a half hour. It was not such that it was a paper of morning 8 to 10 o'clock.  It had been a limited time that even though it had been during the duration of 24 hours, but it needed to be done within 1 and a half hour. This was a very good thing. So, I had known that at such time my electricity would come and that at such time my baby would sleep. And with regards to that I had solved that paper in a very good manner. And there had not been any anxiety and I had done it in a very relaxed way. As there had been electricity and the baby were asleep too.  So, it had been very good that during Covid if this sort of facility is given to us be it even for a limited time. But it is done with our own accord and time. That the electricity be available, and the issues of the home also are solved. So that had been a very good benefit in that paper. |
| **FGD 2** |  | **P1** Miss it was very difficult for us in the online assessment, once we got to give the assessment as a subjective paper. It was very difficult for us to type so fast in a given period and to complete the paper and submit it on time whereas if we would get this paper to write in person then I think it would be much better for us. And later on, the final exam of that particular course had been changed to the Multiple-choice question. This I think was a very good strategy to give the MCQs online as compared to the subjective paper which had been supposedly given to us in the midterm. Because in typing the speed of everyone is very different from each other. So, that was very challenging for us. I think that had been very difficult.  **P2** I think I want to mention one more point over here. That some of the students were very active in the class, whereas the majority of the class used to remain very silent. So, I don't understand the grading or the assessment by the teacher. Like as on which components were the teachers judging the students as some of them were very vocal and they were very dominating in the class. So, the teachers felt that only those were the students who were participating much or were reading and that the rest of the students were not doing such. Then they were making their perception and were trying to randomly pick and ask, so even if they are trying to assess by picking randomly and if the students are not able to give the answer at that moment, it doesn't mean that such student does not know anything. I think this is also a perspective on how they are managing the grading and the grading marks.  Because those who are participating then their grading is being judged by you but those who are not participating then it does not mean that they have not done any prereading. Because we used to frequently get this comment or feedback from the faculty that if you are not participating and if you are not speaking or if you are not giving the answer to that particular question which is being asked that means you have not read it. So, if 3 - 4 students do not give any answer due to any reason whatever their reason or circumstances at their home then it doesn't mean that their assessment is that they have not studied. So, this is also a perception that used to occur by the teachers. There were some teachers who had even taught us in the first semester or had taught us previously. So, they used to have their perceptions too. Like if the student is telling then he is correct. And if these students are not telling anything then it is such that they have not studied. I think this online teaching is very difficult for assessments and grading. To judge every student on the content. So, this was an issue that I had felt when this grading was being done.  **P3** One thing which Sanam already mentioned, it was also my personal experience that at one time the teacher was asking about the feedback regarding her subject. My feedback to her had been that she was going a bit fast, on that one of my colleague, not even my teacher was telling me to pre-read before class, I was astonished at that time as I had mentioned something else and she was teaching to me something else. Upon which the teacher also went into an agreement with the other student that yes, we should pre-read beforehand for an online class. And they did not understand what I had asked upon her asking us for feedback. That is why I would like to appreciate Sanam as she raised this point.  It happens, as one time my mic was not working because of some problem, it happened once or twice only, and I had mentioned it to the teacher in WhatsApp and email. And she did not respond as she should have. With my efforts, I tried and solved things and even told the teacher but there was no response. I would like to add this thing. It should happen like that, if somebody is not participating it does not mean that he is not understanding, or he/she is not reading or learning anything. I think that thing is a little difficult for the teacher as to how to make a criterion, and as to how to grade the person.  **P4** I am agreeing with what Sanam has said. And I would like to give my point of view on this too. That it happens in the group work that the other group persons think that only one person will do the work. And that one person when he/she does all the work then the other subjects of that person are compromised. And the other persons in the group give more time to their personal or individual subjects and do not give time on the group work and the individual who is giving time to the group then his other subjects are compromised. So, I agree with the point of Sanam and am sharing this point of view.  **P5** Yes, Miss . I would also say the same, as the point which Sanam has shared is very good. Because in a group it mostly happens so. And it is a tragedy here in this too. Some people who are marks oriented are so conscious that they do not allow others to do anything either. And they bother the others a lot too. What happens is that they think that what we are doing that is not being done by the others. Even though every person is doing their task but yes let's suppose that someone has some personal problem out of the five group members. That is another thing, but mostly I have seen in the group works which had been done during the Pandemic situation that if a person who extra conscious even more than is required. One is being conscious but those are very conscious kinds of people. So much that they even forget that the person who cannot participate might even have some problem or whatever. But I would specifically say this thing too that there be the criteria to such an extent as much work he/she is doing. So that they can give their 100 %. But mostly those more oriented people do not listen to the ideas or opinions of the other people and do not even allow them a turn to do anything. They just outline the work and the amount of participation that needs to be given to the others is not given either. So then obviously the others feel that he /she is working more as compared to the others. Even though that person is initiating all the things upon himself.  The others even want to do something. I feel that in teamwork the more hardworking face this kind of situation in the group. They feel that they have to do all the work and the other does not need to do anything. So, this is my experience. |
| **FGD 1** | **Can you elaborate on the teaching learning strategies that you prefer with online learning**  **during pandemic and why?** | **In the beginning, you said people spoke about the group work. I would like to ask once again, does everyone have issues with the group work or do you have any other opinions too? Anybody? Yes, all have the same opinion that group tasks or group work have given trouble to you during the online learning mode?**  **P1** Yes, Ma'am. It was troubling.  **So any other recommendations to improve it?**  **P1** Ma'am, as I had told you of the way in having small groups. Then that would also be beneficial. As I had told that my father had been admitted on the 23rd and the second task which was of English that had been a group work of mine. So in that, although I had done my work because it was a group work task then I had given my work already and this situation was such that whole group had known that it had happened urgent and it was such a situation that in which we can give to them, even though I was participating on a regular basis in the whole class. Then the whole group had given me much support at that time, and they had made the whole PPT ready and no doubt that even sitting in the AKU I was dealing with the situation. I was doing the check and balance of it. That this needs to be changed in the PPT or this. But my burden had been left more than half of half. (quarter). If that task had been individual for me or if I was not in a group base. Then maybe I could not have been able to have it submitted. Or I would have felt that I need to be here more at that time. even if it is a small group, because in the group work, sometimes we get leverage obtained too. And sometimes we get stuck. It is not that, we are humans and problems happen. sometimes with you or sometimes with the other.  Then group task is such that sometimes there is a mistake. So, some people are such that they do it typically with every group that they are giving less work.  Then there we face hinders. Then this is said that can we make a group of our own liking? Like there should be group work, it should not be that it is not done. But it should be on a fair basis. That a log is maintained or that the teacher knows about it as which people are working at which leverage and who has which problems or who has only just denied the work for just that time period or that person has this same pattern of work that he is not doing the work. Or that he cannot suggest on anything. Group work is a good choice, because when we do learn within five people then it would be a good learning. Alright? Even though we will have headache. But still we would be doing learning over there. That he would be bringing a different task. And the work would be shared too. When there is a big task, when the work is being done on the basis of very many articles. There is research being done. Then there are quite many tasks. Then in a divided way the work goes on in a well manner. Like our work of NTM was much research based. And we were doing it the first time when it was a group work.  then all had brought small, small chunks of work. and they had done the work together. Yes, there were some who did not do any work, but yes it did not do this that there was a problem, that work had been done. Yes, but with regards if the log is maintained then there can be justification with those people who are giving their whole time. And those who are not giving any kind of time then there a justification should be in it. I think. That those people realize that if the Miss would cut the marks then I should work too. you know that in the lesson and the studies, the marks are hold an importance for each and every student. If we put a 5 marks component in the group, then in which it be told this that all people who work in the group will get those five marks. If they work only then. Then when such a component or such an element enters the group then I think it can give benefit in the group work. This has happened to me in one task. Previously I used to think that, in the last whole semester and whole year that the group work is a headache for me. And that I am the only one doing all the work or there are only a few people of the group who are working, in the group. But then at such time I realized that if my group had not been giving me a support at this time and if I would have been doing it individually then maybe I would not have been able to submit that task. And as the English teacher had said beforehand that we shall not cross that deadline. We were supposed to give it on the 24th and I had been with my father the whole night for the last three days. Then how would I have worked. It was not any possibility for me. |
| **FGD 2** |  | **Probing question**  **Thank you, I just want you to share an example where you felt that the strategy was used effectively or as you said the instructions were not given accordingly or you said that it was not appropriate or enough for you people to understand the task. But on the other hand, in some of the courses, it was effectively done. So, would you like to share an example so that it could help us to see which strategy worked well?**  **P1** Okay, the time when the teacher asked us to move to the breakout room, before splitting us into different groups she had told us that we were supposed to read this difficult part and discuss that and then we will rejoin and we will ask you to elaborate about the discussion that you have done in that small group and she had utilized the same amount of time. Like she had divided the whole content into four parts, each group was supposed to get the list of that typical paragraph and the content, and then that person will come up and elaborate in a combined common group. And then the teacher will add or subtract the pointers that will fall and then those things that `were not clear, she will add some points on them.  So, in the defined period we get some of the information about the other few points or other three paragraphs that we have not learned. So that was a very good strategy used by our teacher. I mean to tell that she is the only teacher who always used to come with a lot of effort and different sorts of strategies. I have just quoted one of them. Apart from that in the same breakout room activity, we have used in other multiple courses, and in that, we were told that we were supposed to read the whole paragraph and discuss the main points that arise. In that, we got lost. Because we had an insufficient time of 30 or 15 minutes, so that was one of the things that could be changed.  **P2** Yes, there was an app. I think Notepad or something. Maybe I am not quoting the right name of it. The teacher told us to prepare a test sort of thing before coming to the class and she just simply prepared the PPT and she just gave us the basic concept of the present class and she directly took us towards the test. I think that was another strategy used by our teacher which was very helpful because the learning concept and the different sort of questions can make us think about it that either we are going with the same concept which the teacher wants to teach us or if we are just going through them superficially.  **P3**  Miss , you asked us whether the breakout room and the strategies were helpful or not. I just wanted to say that using the app doesn't give us the sort of way as what we are demanding but the way the instructions are coming through the faculty can make and lead us differently. So sometimes in a breakout room, we solve nothing because of the instructions that are given by the respective faculty. Apart from that, the same strategy is used in an interactive way and the way we are supposed to elaborate on the things that are being concerned for the typical course. Those are also made and reflected in a different way for me. And in that way, I would say that some of the teachers have not come up with the same utilization of the apps. And there were different meanings and different things which were given during the Pandemic era. But some of the teachers have put much effort, so because of it we were able to complete and cover up the semester. |
| **FGD 1** | **What are your views regarding complete shift / migration to online modality after the**  **COVID pandemic is over?** | **P1** thank you so much, Ma'am. My personal opinion would be that if there would be a complete shift to online in MHN then it would be in those areas where the people are having internet access. As there is Karachi and if anyone goes to the remote areas to do those online classes, then I don't recommend anyone to do this online because it could not be that fruitful for a student like for the clinical experience. Like if there is some clinical experience or there is some clinical practice done or clinical practical done then those are very many things and I do not think that those things can be covered. Like as there was a clinical experience of ours of MHN then we have done simulation-based clinical of ours. But in this, it had seemed that we were just filling the position or completing our time.  There was that. It had not given us any clinical changes. Although it had given us much benefit in the assessment of things. But in reality, the direct contact we had with the patient, those things were not being done. And when next we shall be assessing some patient then definitely, we shall not be able to do it that way as we see an online patient, even if we are doing some tele clinic then it is something very different related to the real patient. So, these are all the things. So, it is this that if it is going to shift to post online then I do not recommend it. Because of the outcomes, the clinical outcomes those things could not be coped up. That they are able to complete it. I do not think that those things would be so fruitful. Because our PostRN is totally practical based blended learning.  **P2** Ma'am online some things can be done, but they cannot be done completely. So, until hands-on practice and till we do not see the patients then we do not understand the things. Until we do not work face-to-face, or we do not work hands-on then we do not understand. Because we are habitual from the start in project learning. So, there are many such things that we do not understand online. And there are very many things in this which we have not yet learned in the last semester. Because completely clinical online and a good talk with the patient, and not done any assessment of the patient and nothing has been done in a proper way, so it had not been enjoyed. That as we are learning this in a proper way. That we are doing a hands-on practice on the patient. And clinical itself means that we have to go to the patient and meet them. To listen to them and their complaints. And to deal with them in that way. Like in the teleclinics we last heard the patient's views when we were doing our senior electives, then there were some objectives in it.   So, in that, the patients were saying just this that the doctors do not check us and they just speak on the phone, so we are not satisfied. And then they are taking the full fees. Meaning everyone wants that there be a face-to-face thing. Only a little bit be online but not completely.  **P3** If in the future it is being considered to be a complete blended thing then we cannot do anything in this seeing the situation. Like as in the Covid Pandemic situation start we had some courses of the first semester left. When our lead and some faculty had shared this thing with us that if you are given SOPs and consent be taken then would you be ready to do clinical?  So, honestly, nobody was ready for it that we cannot take any risk because security and safety are in first priority. So, no one was ready. So, all our things were changed to virtual. And we had a lot of fun in that too. Post RN and BSCN level then maybe I would have said no. Because I do not feel a sense of duty with the patient. But we are of the level of PostRN level and we have done our duties before and we have that experience with us that how we have to speak with the patient, and how we have to meet them and as to how the care of them is done. We already know how our time passes with the patient. So, I do not think that I speak on this point of view that I am not completely agreed with this. If this situation still stays, then my own priority and safety will be first. So then how can I do it.  **I am sorry, I am interrupting you Parveen. The question says that what if the Covid Pandemic is over and what are your views if we shift completely to online mode even after the Covid is over.**  **P3** No, if the Covid does get over then we would not want to stay on online mode. |
| **FGD 2** |  | P1 I think to go completely online must not be done ever. Never. I would never ever support that postRN course should be completely turned online because there are lots of difficult and advanced concepts in the postRN program in which the student need teachers to physically explain and some of the concepts are there which need to be clarified again and again with the teacher in person or sometimes some of the clinical skills are very important. I think that face-to-face interaction importance, which is prevalent, should be there. Because in the face-to-face interaction, the teacher and student relationship along with the interaction in the university campus, has to be there. As the environment is very conducive to learn some of the resources and the facilities that are available in the campus physically, as like there is the Library, then the access to the internet and the computers sometimes for the group activity and sometimes in the schedule of timetable we get free slots for combined study, for group study and for completing our assignments. So, I think to have studies completely online should not be done. Not at all.  P2 Yes, I would completely agree with the point of view given by Sanam. That it should not be completely online because there are many tasks which we have to complete in the university, or we have the need of the university for them. Like as there is our library, then the group work, especially for the group work, because when we have to do group work or group task online then that is obviously also graded. And if there is one group of 5 to 6 persons then it happens that not one person gives their participation properly on the work and the work of the 5 persons has to be done by one person and it becomes stressful for that particular person, to manage such a situation.  And then there are other many reasons due to which it should not be completely online. There should be face-to-face classes as well. In face-to-face I would like to add this too that there be face-to-face also and then as our lectures are recorded now those be kept continued even in the face-to-face classes also then it would be very good because the students get something clear at that time but later on if they forget then the recording would be available to them to remember. If this option stays continued even in the face-to-face classes, then it would be very beneficial. Thank you.  P3 Miss , as per my opinion I think that complete shifting will not fulfill our criteria of clinical learning. Apart from that when we are graduated from an institution, we are updating our theoretical knowledge, we are being called as graduates for a defined course so that should also reflect in our personality. So, such a typical thing can only arise when we have interacted with our patients. With that sort of mindset, we can only achieve such a goal by interacting not only with the patient but with our peers and colleagues and with the faculty. I think that for the personal grooming coming to the campus and meeting different sort of people and then exploring as what their thinking is, regarding the same concept or a different situation in a clinical area which can not only enhance our clinical skills but also, I think will be reflected in our future practical life too.  P4 Miss , there is one thing which I also want to mention here is that going completely online would be very difficult and it shall also not be very fruitful as compared to face-to-face but one thing which I also want to appreciate is that if we are going to talk about a teacher and faculty. They gave their best even in online classes. They tried and we do not have any objections with the teachers, but the problem arises in the connectivity issues, as we are living in Karachi then sometimes there are electrical issues. Where I live most of the time there are no electricity issues but with many of my colleagues who live very far and have electricity in their areas, they go to AKU early morning because there are electricity and connectivity issues. |
| **FGD 1** | **What are your recommendations to improve remote teaching and learning?** | **P1** My recommendation for this would be that even if we shall be having this in remote areas too. Then as AKU main has a branch in Chitral. Then we can do something like this that there be some such person who is of the master’s level or on the faculty level who have things under them. There be a supervisor so that the clinical practice and all those things are done under that person and it is of that level that is the criteria of AKU and that it can meet it. Then on that basis, it can be done in remote areas. As we have the Agha Khan in Bunee. Aga Khan diagnostic centre in Bunee. And it is also being made in Chitral. Then under their supervision, we can do these things. And my recommendation would be this too.  There should be some facilitator over there. That there be any facilitator over there that can take our recommendations at the level of AKU. As to the guidelines of AKU and by following those the student be enhanced, their knowledge also is to be enhanced. And secondly, the other option is that if there are online classes being done in the remote areas for the clinical practicals then the faculty go there and assess those people over there.  **P2** Ma'am the disadvantages which we had mentioned then if we cover up those then the other net issues which happen and if those are strong then online learning can be done. Can be done easily, if we cover the disadvantages.  **Parveen had said in the chat box that part timers and full timers division should be balanced. So, this is another recommendation. Parveen can you just elaborate as what are the problems of part timers and full timers. How is the division not balanced yet?**  **P3** Yes Ma'am, I was saying this in this sense a bit that our faculties do not know as how many of our students are part timers and how many are full timers. So last time when my group had been made then unfortunately it had been such a group in which there had been a group of four people, I was the only full timer the rest of the three were part timers. When I used to ask for their collaboration, they used to really get angry that you do not know, you do not work. We are in areas, and in the clinical, so we do not have time. So, I had spoken specifically with the faculty on this that in my group there are four people and all four are of duty, so their timing is not coordinated with me. So, how can I do group work with them. On that the faculty told me that while making the group it had not been in my mind as how many of you are the full timers and how many are part timers and she had apologized for it.  **P4** Parveen actually talked about the same thing. Which I was about to say, so that’s okay.  **P5** Yes Ma'am, it was simple that there should be group work, very many things are cleared in group activities. As I had been in Chitral, it had not been possible for me and there had been only one person with me, Sultan. And in a group activity thee should have been some such task that I and Sultan could do. Or there be such people who would be in coordination with each other closely. Then there should be a group of those people. As Mehnaz had mentioned it too. That a group of such people should be made who can understand one another. It was also my issue that if I would have had my group with Sultan, that he would have been in my group then I would have found it much easier to do things along with him. So, it should be seen in this way that the coordination-based group is made then that would be fruitful. And then there is me in my group and second was Nazeena. And we both were totally different, and she also had an internet issue and I also had Internet issues and the rest of our group people were here in Karachi and it had been very hard for them. So, if I and Sultan had been a group and Nazeena and Shehla had made a group then they would have found it much easier. Because then we would have been able to contact each other directly. |
| **FGD 2** |  | P1 I think to improve group learning especially online, rather than marking criteria for a group to assess any group activity or any group task. The criteria should be transformed into individual criteria for that. And the component for the group should be a small chunk of that component. Where in a group task every individual person's contribution count. Because when we do a group task or when there is a group assignment to be completed then the marking for each individual is given as a group. In which some of the students take advantage and the others on which the burden is laid. As some are very much marks oriented, then those take the whole task on themselves. But whereas the marks are equally given to all as a group. So, in that online, if group learning is to be done then its criteria for marking should be individual. And then in it, there should one component included of group participation. So that each student can give his 100% in the group activity. If it should be the group assignment. So, I wanted to add this.  P2 Yes, and in it the individual criteria marking chunk be increased as compared to the group marking and rubric. Because when the rubric of the group is increased then the people take advantage of it from those people who are more hardworking, or the other colleagues seem to feel that they shall do all the work. So, they just participate only a little and do not give their 100%. Because they know that the major component of marking shall be given as a group. So, they take advantage of others in it. So, if it would be defined as an individual, then everybody will give 100 % of it.  P3 Okay we follow a strategy of the clinical log that you give a log of your work too. So, I think that this log is also not any true reflection because the faculty gets what is written on the paper. But that is not the real picture of teamwork. That is only that one has made, and the log is also taken as a task, as this is also one of the tasks and if you have even made the log then your participation is counted. So, the recommendation can be this too that rather than putting it on a paper and sending an email to the teacher. I think the teacher should have a small meeting with the group and to analyze and assist the teamwork if she wants to mark the group on the teamwork also. So, rather than putting it up on a paper, it should be a verbal interaction with the faculty to make a transparent and clear assessment of working.  P4 Yes, I just wanted to add another thing that we have just spoken about group activity. We could also be given a chance to make a group on our own. It can increase our learning in a way, as when we choose our group peers on our own, we select those members with whom we feel comfortable or with whom we have some understanding. We think that our understanding matches with the rest. So sometimes this can bring more effective results as far as our grades are concerned.  P5 I think that in online the teacher should use different online modalities as well during the class rather than only the PowerPoint presentations, because what happens that the class is so lengthy that to see that PowerPoint presentation and to grasp all the concepts only from the PowerPoint presentation. It is very difficult. So, rather than only using PowerPoint the teacher may use different online Apps as well to design some quizzes. like there is Kahoot, then there is Quizzes’ online App. Then there is NOTE and then Mentimeter. Then there are live zoom polls. So these are some of the digital online Apps that should be used in the class to make the class engaging and interactive, because once the teacher asks the question then okay can anybody share their point then what all the students do is that they mute the mic. and the teacher is continuously asking who would like to speak, but nobody will give the answer. But if she will use digital Apps in the class then what will happen is that it becomes mandatory for every individual to participate. so I think different digital Apps could improve online classes as well. And will increase the interaction of the student in the class as well.  Even this Miss . The teacher can use a chat-box also. Because any comments which come in the chat-box it's with the name of the student. So, it can also increase the participation and assessment that the teacher also can get help in the assessment. As who is participating. Sometimes the students are not comfortable speaking, but they are very good at giving the answers in the Chat-box. Because it is sometimes very challenging for the faculty too to learn the digital app and to implement it in the classroom, because the digital app needs some of the technicality and we should have a very good hands-on on that App otherwise your time could also be much wasted too if it is not used properly by the faculty.  And faculty should also give good instructions as to how to operate all these digital Apps in the class because only that can help the students to learn better. Because some of the digital apps are very good access to laptops but whereas some of the Apps are very good access to mobile phones. So, the hands-on skills of the user should be very good of even the faculty themselves. And she/ he will be able to give them clear instructions as to how to use these digital Apps in the class to the students as well. So, it will utilize the effective time of the class as well.  P6 Yes, Miss . I was about to say the same thing that Sanam mentioned that teachers should be trained on how to utilize these Apps and how to make use of those things also. As I have seen multiple times that we were given some instructions to come up with this App and we were supposed to download it and we were told that we would be using it in the upcoming class in a day or two. Then what happened in it is that when the class started, then sometimes the teacher used to say that we could not be able to use it properly because these things are also new to me. So, for the first and second time it sounds good as alright fine we all are new in this and we are not used to working on different sort of Apps, but it alright to hear it once or twice but when it becomes a repeated thing to happen the third or fourth time then you get like as, why now? Then what happens that in the defined time when we are supposed to cover the content and we are told that we shall hasten in completing just because a lot of time had been wasted in the utilization of that App. So, in that thing when we are doing everything in a hurry then we cannot grasp what we are being told in a good way, there is an issue than in the absorption of the concepts in the mind.  Apart from that as you were asking the strategy then I would like to add another strategy which is used by our respected faculty and what had been done in it was that she had given some different sort of manual tasks and she had said that you are supposed to take a pencil and pen and do all these things on your notebook. And click a picture and send it in the Zoom chat group. So that had been something new and that had utilized our time in an effective way. Because chatting in a Chat-box and using the gadgets or typing something via your keyboard or something, then all do not have a very good speed in typing and in online work that too had affected us a lot. And this had been done by the same faculty and apart from her, no one had used any such strategies.  P7 I just want to add to improve or to recommend that we just need to limit the time also in the online classes to improve the classes and also limit the time for the e-resources. Because once the teacher will ask us that this is an online module and so the student has to read all the resources before coming to the class. So, those resources are so many that the students do not read after seeing such a large chunk of information. So if instead of giving 4 or 5 articles if the faculty just give only one effective article, which is the most relevant to the module than I think that such time of ours would be effectively consumed rather than watching 3 or 4 videos in one module and the five articles in the same module and then give the PPT on them. And then afterward the Quiz and pre-quiz.  So rather than having it so much condense online if that can be reduced a bit and the student be given the time to grasp the concept, even from a bit of a chunk then I think it would be better to concentrate in the online classes as well. Sometimes there is so much content that the student feels that he/she does not study. That they open the online study, but some students just close it when they see such lengthy online resources. So, I think to improve the quality of the online, we have to divide its content into pre-module and post module. Like you decide your resources to pre modules that before the class such resources are necessary and then after taking the class these are the necessary resources in the post, rather than giving in the starting that all this needs to be studied beforehand. |
