# Supplementary material for: Beyond the classroom walls: Stakeholder experiences with remote instruction in Post RN baccalaureate nursing program during the COVID-19 pandemic: A qualitative inquiry
Source: PLoS One. 2024 Apr 4;19(4):e0300007. doi: 10.1371/journal.pone.0300007 (PMC10994296; doi:10.1371/journal.pone.0300007)
Supplement: S2 File — (DOCX) [file pone.0300007.s002.docx]

| **Interviewer:** Ms.  **Interviewee:** FGD of Faculty  **Topic**: Online Teaching |
| --- |

**Interviewer: My first question is, what are your views about online teaching and learning? And you can use the feature of the zoom hand so that we can see, and we can take your responses. I will repeat my question. What are your views about online teaching learning?**

**Interviewee:** Aslammu Alaikum, welcome to all the participants coming from my viewpoint on online teaching and learning is that despite being a very effective, very innovative, and modern strategy of teaching to a group of learners, yet it has many prerequisites. I mean, it's effective and at the same time, it's very demanding. I can say that there is a set of tools or skills which are required to make it go smoothly. Otherwise, it can end up being a flop for you.

**Interviewer: So, thank you, X, let's see what others think about it, yes X MsRZ over to you.**

**Interviewee:** Thank you, MsR. I totally agree with MsM that online teaching is an innovative idea and strategy, although sometimes it seems easier, but at the same time also challenging for us and the students as well to have knowledge about technology and how to use it. If we are using them for the first time or they have used them, they are not available all the time. The connectivity of the Internet was also one of the matters in smooth teaching. Yes, I totally agree with MsM.

**Interviewer:** **Right. MsRZ. So, it seems easy, but sometimes when it comes to practice, it is difficult, right? So, yes, let's take the response from X MsN, and then we will come over to you MsR. Yes, X. MsN over to you.**

**Interviewee:** Thank you very much. I think it's the total paradigm shift both for the teachers and for the students. We as teachers have been used to teaching face to face since we joined the profession. And the students are also used to learn face to face since they have joined the schools.

So a paradigm shift requires a mind change and a shift from Face-To-Face to online is actually required, would require some time for adjustment and for, getting acclimatize to the screen that we have in front of us and then shifting our focus from the face to face to the screen and then teaching on the screen. There are pros and cons for online teaching. So, my first point is the paradigm shift and change requires time.

So, the teething issue. It would take some time. And this transition has challenged both the students and the faculty, the students are not. Some of them are and we don't know. Because we cannot see the nuances, that is, the teachers are struggling with the technology, some of them are thorough with the technology, some of them struggling with the technology. People like me who I would say are fossilized with and this relearning is becoming unlearning and then relearning is becoming a big problem, a big issue, a big challenge.

It's, again, a mind shifts from what we have been, how we learned to teach and how we are teaching right now. So, the way we are teaching right now has challenges, although some aspects of online teaching are better as compared to face to face. But still, the challenges are there. So, my response to your question, what do you think about online teaching is this. So, I think you would further ask about challenges and then we can talk about them.

**Interviewer: Thank you, X MsN. We're like we'll talk about challenges and the pros and cons. But what I have gathered from your point of view is that there are some like changes or transitions that take time and some of them are good at adopting that change or going through with this technology. So, we'll see what are the challenges in a while. So, thank you, X. Let's take the response from X F.**

**Interviewee:** I wanted to say the same thing, that this online teaching and learning is going to take time, so we need to follow whatever is in the trend and whatever is the most viable and feasible option for the teaching.

And with that, I also believe that change is always difficult, whether it's face to face teaching or online teaching, or any speaking methodology. So, whenever a person sees a change in his or her life, it's. It's always difficult to adapt and to modify our skills in this specific teaching or teaching-learning strategy, the most important things are the resources and the expertise which contribute a lot. And because of it the teachers, as well as a lot of students are suffering, thank you.

**Interviewer: Right, so expertise, skills are required to have this transition. So, let's take a response from X S. Yes, X S?**

**Interviewee:** Good Morning! so online teaching and learning is the faculty delivered instruction via the Internet and we all know how a what is a competency on the technology?

Online instruction includes real-time that is synchronous as well as asynchronous interactions with the students. In a way, it benefits both students as well as faculty. Online teaching provides instructors with greater flexibility to address learning styles and use different technologies. But looking at our education system when the students are coming to us, we are not at all exposed to online teaching. So that is the biggest challenge for us. (voice was gone again)

Effective, if so, that requires a lot of, you can say, a lot of preparation, institutional support for how to develop the course, because whatever we are doing so far in online teaching, mainly most of us are not trained. So, we are doing it online teaching by hit and trial method. Yes, we have done some workshops, etc. but that's not enough. And mainly online teaching is the one more thing that is required for the success of this online learning.

Online teaching, learning is your technology control and how much you can motivate and get your students engaged and learn more. And more learning, material accessibility, computer availability, instructional design. So, all these are very, very important in developing, teaching, and learning online. And that requires a lot of time. So, I feel it's a challenge for us because it is something new which we have introduced with our students and they are not used to it. But it is going to take time. That is going to be a lot of time taken when we will be having this as we will be more relaxed and comfortable with this education system.

**Interviewer: Right, X S. So, this means that it's very similar to the other points, that it requires skills, resources, and training to teach students online. And on the other hand, students are required to get exposure to it so that they can learn in a better way. So, yes, what do you say? Yes Sir, MM?**

**Interviewee:** Yeah, I would first like to say that. Good morning, everyone. And regarding the online teaching, according to the question which you posed for me, It was a new experience and it's also a new experience for the participants. Initially, we were juggling with technology connection problems, students had problems, they couldn't hear. These are the normal problems that we had, but somehow, I was able to give the content. But as a language teacher, I feel that in online teaching we lose out on other things like the student's emotions, the physical gestures, we cannot captivate the certain temperaments. Which are, I think very essential for the teaching. But taking on a positive note I think, perhaps we need to educate ourselves more on this because in the future we will be using it, It will be a part of the system now, the online teaching. And I think as many of you have already mentioned that we need to learn more about the technology. And it's not only us, the students as well need to learn on coming to the online system.

**Interviewer: Right Sir, A very important point. Yes, you have discussed that it's very important to understand their emotions and what they are going through, and what is back and behind the screen. So that we cannot see, and we cannot feel. Yes, this is another thing that is required when we talk about online teaching and learning. So, thank you for your point, Sir. MM.**

**Yes, next. Yes, A?**

**Interviewee:** My point is that I think as I am a teacher of my education technology course, so the students and the faculty both need to take ongoing training as which can help them to improve their skills. I have learned this thing from the course which I have been teaching to the students.

Because we have to teach them online. (Recording thwarted a bit) There also I have trained the students plus the teachers ongoing during the online courses. I think the online going training should be done all the time. So, then every time you would learn something new.

**Interviewer: Right. So, training is required, and you can say the ongoing and continuous professional development is required in order to help the teachers to teach in an online mode. Yes, X S is there anything else, Yes?**

**Interviewee:** I just want to add to what A is saying, that online instructors need to keep in mind that active learning is the one of the keys to success in the online classroom and instructor needs to employ new techniques and technologies as a strategy to ensure that their courses make the best on online media. So, this is also very, very important.

**Interviewer: Right, so as we discussed the training is required, ongoing continuous professional development is required, so what is needed in those training and what are the faculty needs? Can you just elaborate on it? This question is for everyone.**

**Interviewee:** Could you repeat the question, please?

**Interviewee:** Or could you write the question on the chat so that we can all read it?

**Interviewer 2: Sure, we will write that down, or perhaps rephrasing it a little, what should be the content of the training that you think would be very much needed for the faculty? So, should it be around tools or should it be around techniques of teaching or should it be around the blend of both? What should the content of the training be?**

**Interviewee:** I think it should be a blend of both and the main thing is to accept the technologies. But most people do not accept the technology.

**Interviewer: Right so acceptance is what you mean. That some people accept it easily and some take time to accept it. Now let's see what X S wants to say. Yes, X. S?**

**Interviewee:** what I wanted to say here was as I just said in the beginning also that we just have to see our curriculum. We need to redesign our curriculum and we need to see how much institutions support is there. We should have an online learning centre if we are planning to have our curriculum online. What will be taught online and what will be face to face. How to develop the courses according to the online teaching and learning because now the courses which we have are not based on the online developed course structures and not only the faculty support but also student support and then as A also mentioned that the technology control is very important. And learning material accessibility and connectivity and computer availabilities, then instructional designs which you are using, these are all various factors which are required for the online teaching method.

**Interviewer: Right, when it comes to transition we required curriculum and we require changes in our courses and also as you talked about the resources, so all type of resources as human resources as well as the other resources like the internet and the computer availability, right. So yes, X**

**Interviewee:** adding onto X S's point I would like to say that specifically in the course we have for example, in the nursing courses, we have the theoretical concepts as well as the clinical parts. So, for the clinical parts, we cannot switch completely to the virtual clinical. In clinical as we know that physical clinical teaching is significant. It's very important for the students to learn things by doing them, so for that clinical component and face-to-face interaction is very important, in these circumstances we need to modify which we or revamp the strategies or the content in a way that we give a blended opportunity or options for the students, so this is the way we need to revamp our curriculum or our course according to the need of the time. And I also think that the content training for the faculty which is required nowadays include the technologies which we usually deal with when we interact with the students.

So, we have zoom, we have MS teams, and other modes of communication or connections through which we connect with our students. We need a third training in getting hands-on to such modes, through which we connect to the students and we also need to have training on different strategies or techniques which are useful nowadays. Yesterday we had a chance to attend a boot camp that was regarding voice discussion. voice thread discussions, so this was a new strategy that we learned and except the writing discussion forums which is not very useful. and the research also proves that it’s not very interactive because students do not take part, so much in writing discussions. So, this was one of the strategies which we were taught in the training. So, the content of the training should be based on techniques and application software’s which are really very helpful in communicating our content or in delivering our content to the students.

**Interviewer: Right. So, thank you for elaborating, on that question that content training is required especially the online tools that can help you to teach with these platforms like Zoom, or the Microsoft team**

**Interviewee:** Thank you, MsR. Mostly all the points have been covered but my idea is that Maybe we cannot list out things and get fixed on that list that this is something that is required in the training, the training can be of variety depending upon the learners need. Learners as like us, the teachers. So, if I need something and I need that training and if somebody else does not need that particular thing but something else but then we have to draft training according to all the teacher's needs first of all. And it can be a variety of needs. And then maybe it is again then evolving, so as we said earlier it is an entire paradigm shift and it is something new, we are getting into. So, the training can be according to the need and they can get evolved with time but today I required this new thing which I wanted to be trained about. But mainly, yes, it’s all about what we learned in face-to-face teachings. How to deliver our teaching content and then as to how to allow the students to get engaged. How to relate our assessment techniques as related to what we have taught online. And as to what several learning strategies can be used effectively, so these are some points that are broad enough as training points, but it all depends upon faculty needs. And the new up comings.

**Interviewer: Right. Now that’s what we discussed as continuous professional development is required but a very important point that you discussed is that need changes might be such that some people need help in content and some people need help in technological resources. So, yes it matters a lot, and training should be based on these needs. So, let's take two more responses to this question, and then we shall move to the next question. Yes, X MsN, over to you.**

**Interviewee:** Yeah, thank you I just want to add much has been said and quiet appropriately said, I just want to add that while this is a skill and this is a new skill for all of us, so the training should have some portion focused on troubleshooting.

**Interviewer: Okay.**

**Interviewee:**

**Interviewer: Right. This is a good suggestion that we have noted down. So, I think Sir, MM, and you are raising your hands. Let's move to the next question. It would be great if you can just write it in the chatbox. keep writing your thoughts in the chat-box and if there is any else that you need to add you can later add that question. So, the next question is, what are your experiences of online teaching during the Pandemic. As you had discussed that it was difficult in the beginning, you were juggling with the things, you can elaborate with your experiences. So, who would like to go first? X. MsN are you raising your hand for this particular question**

**Interviewee:** Okay, so my view on this is that the experience is a new experience every time. First of all, whenever I had all the instances where I have taught online then my first reaction is that I always get nervous. The thing is that I don't know whether the class will go smoothly or not? or if the internet would work continuously or not? Either the electricity problem would be there or not? And things like that. So, first of all, there is a great deal of uncertainty whenever we start class and throughout the class, that uncertainty remains till the class ends. So, this is the first thing which I have experienced every time. So, my anxiety seems to be every time when I am teaching online because I am not sure how my class will go. After all, in face-to-face, we are very pretty sure that okay, we are prepared and that our students are there. We are here and this is how, and we can imagine, and we can envision how our class will go. But online it is the very thing that we don't know.

**Interviewer: So, the uncertainty was too much. And you were not sure as to what would happen next.**

**Interviewee:** Yeah, can you hear me now?

**Interviewer: Yes.**

**Interviewee:** I agree with what X. XXX has said. And the thing is, what my experience was that, when I taught the content, I was not sure whether the students were following it or not? They are all saying yes, so you see it was very important for me because I cannot really see them and so they just are probably saying yes, some of them, and I don't know whether they are following the matter. Because when I set them on a task and assignment, I didn't get what I actually projected. So, I was a little disturbed about that. The other thing is that students often tend to play up, they come online but they disappear. And which means that I have to repeatedly take attendance. And these were the issues and it becomes a disturbance when we are actually set on the content and we are teaching because I basically don't know whether the students are following. So that was my concern and some of them don't even appear on the screen and if they do or seem to appear to be online, but they are not there.

**Interviewer: So, the first thing that you mentioned is that you are not sure whether the learning outcomes have been achieved or not from the student's point of view, and then when the student seems to disappear that is another problem and a big concern.**

**Interviewee:** Yes, yes.

**Interviewer: Right that has been your experience, so let's take the response from Ms X**

**Interviewee:** Thank you, MsR. Yes, I totally agree with Sir. MM. I just wanted to elaborate on what Sir. MM has said. We have also faced the same issues of student disappearing and the connectivity of the students while we are teaching, I am a teacher who is teaching at the clinical point. And clinical is very important. Virtually teaching the clinics are as much as challenging as the theoretical component. We used to have a physical clinical where we have a physical bonding with the patient and with the students as well, we could see their emotions, we could see their anxiety, we could see their fears regarding the patient care. But in virtual clinical, it would be difficult for us or I felt difficulty in having that connectivity with the students especially in the post (voice thwarted) furthermore again students are disappearing, physically you may see on the screen the students are present but when we called student name to contribute their learning during the class or during the clinical time, they don't respond that means they are not physically present. So, it was a challenge again. Further during the time when we are teaching an important component the students we find are moving back and forth. Coming and going. And they are waiting in the lobby, the prompting of the students who are waiting in the lobby on the MS team is disturbing and that distracts us in our teaching. This thing we have felt strongly. Thank You.

**Interviewer: thank you, X. for sharing this that the movements of the students of coming and leaving due to any other issues, internet or any other thing behind the screen that distracts a lot. And that is another concern which you have added to Sir. MM's point. So, let's see what X. MsM thinks about it. And secondly, I would like to just further add to my question that does this affect your life in any way, and if yes then how, and if not then why not? So, you can even talk about these as well. Yes, X. MsM?**

**Interviewee:** Yeah, as far as my opinion is concerned well before I share my experience with the group, I completely agree with what X. XXX said, what MM said and what MsRZ has just added to the discussion. And then adding onto that my biggest challenge and the concern that I faced was that it is extremely challenging to engage student's mental presence, attendance is an issue of course sometimes they are joining in, they are appearing in the participant's list but they are not there. I don't know, they simply log into the class and then they are not physically there and if they are there, they are not paying any attention. There may be several other screens active on their systems, who knows because there are a few things which we can hardly control when it goes to online modality. And along with this, I mean even if they are there. The effectiveness of their participation is not there, unfortunately. There are a few sincere students who would take charge of their learning, who would produce what you are expecting from them but the rest of the group and you know, I can say that if it's not majority it's at least half of the class who still does not consciously take any charge of their learning when it comes to the online learning, which ultimately would affect the learning outcomes of the course and something that they would produce in the end as the finished product will not be up to the mark. And that is where they clearly say that we are not satisfied with this modality and we really wanted it to be a face-to-face class. In a class we can control them, I mean, we can restrict the distraction.

I mean, we can have a say on what they can do and what they can't do. Once they are in the class and of course even while they are sitting in front of you, you cannot ensure that 100 % of students are mentally engaged in the process of assimilation but still if you see any kind of signals through their body language or their gestures you can still bring them back to the track. But here when they are not there. You are just listening to them saying Hmm, and this and that and the rest of the things you are complete, I mean, you are blind about it. You cannot control it. So, the attendance is there, the power supply is disturbing, the infrastructure itself is not developed in a country like ours. Along with this when the students are not ready to take charge of their learning consciously and sincerely you sort of find yourself handicapped to some extent.

**Interviewer: So, you mean to say that some of the students are good at self-based learning but the majority is your concern because it is very difficult to grab their attention on a screen, and X. A has even suggested one thing that we can have program assistants who can help the teacher in taking attendance and keeping a check on them.**

**Interviewee:** No, marking attendance or taking the attendance is not an issue.

**Interviewer: Right.**

**Interviewee:** By looking at the participant’s list we can still gauge that. But the point is, Yeah, mentally they are not there.

**Interviewer: I got your point. So, it’s not only the matter of the attendance but you need their mental presence so that you can at least know what they are learning and like Sir. MM said that you are not sure what the learning outcome is, from their side. As to whether they have achieved their objective or not? Thank you, X. MsM. Let’s take a response from X. F and then X MsN. Yes X F, over to you.**

**Interviewee:** Thank you so much, MsR. I would categorize my experience into two categories, so first of all when I interacted with a large group, let’s say, for example, 70 students. At that time my experience and my feelings were completely different. And when I compared this group with a group of 14 students the experience was completely different.

**Interviewer: Okay.**

**Interviewee:** So, in a large group I had the same issue, I was thinking that I am talking to the walls because at times when the concepts are so that, you know such concepts as you are introducing a disease process which the students don’t know. In that class, it is very difficult to make such classes interactive and in that, you had to say a lot of things and you get a feeling that you know nobody is responding. Either they are listening to you or not. That was the feeling that I had when I was taking a large group class. But then when I switched to smaller groups, smaller clinical groups, so, in that, I felt that we were able to connect. There was a human touch. You were able to see the students. The students were able to connect with you. They were very participative. In that, I felt that I can connect with them, I can achieve the objectives which I wanted them to learn. And listening to their comments, their feedback, their appreciation after every class was very much a booster and it pushed me to further plan effectively during online classes. So if after three hours or two hours online class compared to a larger group I was not much satisfied because I was always thinking and I was dubious about that, either that the students were able to learn something from the class or not? Or the entire hard-working that you have put into making the presentation and the delivering of it, whether it was wasted or not. So, this was the thinking that I had in a larger group and a smaller group.

**Interviewer: It is a good comparison that you have shared, it is easy for you to connect with a smaller number of the small size of a class as compared to a larger group. So, that is a new thing that we have noted. Thank you, X F, and yes X. MsN over to you.**

**Interviewee:** I would add just one very personal experience because much has been said and all that has been mentioned is a very true reflection of a teacher who has been asked to switch to online teaching. The best part of online teaching is that I learned using a whiteboard, I learned to draw on the whiteboard. And that was giving me an elated feeling like this that I am now able to understand things. as how to teach online. But on the other hand, my teaching experience online was that all the teaching focus was on the teachers to teach online but to as how it is being received at the other end by the student there was no focus on the student. So, training sessions for students in how to receive teaching online or how to learn online is very important because the entire focus of most of the institutions, because I have teachers in my family, the entire focus was on the student. I never heard about a workshop that is designed for the parents, that is designed for the student in how, to be honest in online learning, and how to respond to online learning. How to make the most from online learning. So, this is what my personal experience with teaching and learning online is. Otherwise much has been said. Thank you.

**Interviewer: Thank you X MsN. So, from the beginning, we were saying that training is required for the teacher, but this is something that you have added that it's not only teachers who need to give input it's from like needed from students side as well and there we can have a balanced relationship between a student and teacher. So, it is good that you have brought that point up. Let's move to the next question and as in the beginning we started about discussing the pros and cons, now it's time to talk about it in detail. The question is in your view, what are the advantages of online teaching in the Pandemic situation?**

**(silence)**

**Interviewer: I shall repeat my question that what are the advantages of online teaching in a Pandemic situation? Yes, X. MsM?**

**Interviewee:** Well, despite all the uncertainties and the uncomfortable feeling that we initially had with this online experience, the online modality kept us connected. And to some extent, we were able to fulfill our objectives for each course and we could bring the students till the end of the semester. Because if we had waited for these face-to-face classes to open, we might still have been waiting for our last semester to end. With the help of this channel of online teaching, we could at least get to the finish line. So, we kept connected.

**Interviewer: Right. So, it helped us to remain connected with our students, and let's see what X ?**

**Interviewee:** Yeah definitely I agree with MsM. This is the only thing that we looked upon during Pandemic where coming face-to-face and getting together was not possible so this is the first advantage of online teaching that it can happen from anywhere and anytime. I guess this is one and the only big advantage of it that it can happen anytime, and it doesn't have to be time-bound and space-bound and presence bound in fact, also. And it's not that you have to stay face-to-face all the time you can record your things and send them the way we do asynchronous teaching. Yeah, this is something that is very much helpful about it.

**Interviewer: It can happen at any time and place. And we can record and revisit as you said. Yes, X. S?**

**Interviewee:** I just want to build on what XXX has mentioned. That it was flexible.

**Interviewer: Right.**

**Interviewee:** It enabled the teacher as well as the student that they were able to set their own learning pace and added flexibility in the schedule also as well as in the agenda and I feel that there was no need to give anything up. Studying online teaches you time management skills which makes finding good work and study balance easier. And I felt that in it there was one thing as it was flexible but at times this flexibility was also a disadvantage as sometimes you were not having any connectivity. whether it is synchronous or asynchronous. So, that was one of the challenges that we had to face.

**Interviewer: Right. So, if we summarize what the advantages are? Sir. MM has also mentioned a very good point that we start the race and a lot of people have agreed to it that we are able to start the race and then finish the race, so that’s a good point to summarize it. Let's talk about some disadvantages, what do you think are the disadvantages as X S has started with one you can add up on that or if you have anything else to say. Yes? So, flexibility is one thing and advantage but on the other hand when it comes to the internet and everything this turns into a disadvantage. What else?**

**Yes, anybody who would like to go first?**

**Interviewee:** MsR can you just repeat the question?

**Interviewer: The question is what are the disadvantages of online teaching in the Pandemic situation? So, you can talk about the change in the routine or any other thing that you have experienced. Yes, You can take turns.**

**Interviewee:** Looking at the disadvantages I felt that it was time taking duty online in just having a full skill on IT usage. Second the internet connectivity and electricity problems these all had ended in the time taking activity if we have an example when we are taking post RN clinically-based studies online exam and at that time, we also felt that the students had issues of internet connectivity. And we had to give them 15 to 20 minutes slots to interact with standardized patients and at that point in time if the student has not connected, we have to call students and ask them to connect and request them to join the case -based that your time is going on and that it is your exams time. So, that was a big challenge for us, and that exam which had supposed to be ended as a three-hour activity. It took 4 and a half to five hours to complete it. So, I think that was the time and issue for us in the clinical setting.

**Interviewer: So, we can say that it is time-consuming. Right?**

**Interviewee:** Yes. It is time-consuming and it is also disturbing the whole schedule. If your time is planned for three hours and after three hours you have another clinical conference, then you have to shuffle that clinical conference on the next day. And have to adjust it to the next day. So that was a, yes it was a challenge.

**Interviewer: Right, thank you X and yes, over to you sir ?**

Interviewee: Can you hear me?

**Interviewer: Yes.**

**Interviewee;** Right. There is one thing which I would like to note, it's like a little personal note. When we were just face-to-face, we the ELT group. we were teaching as a team and so we were teaching the same content to the different sections.

Like when we were teaching face-to-face, we the ELT group we were teaching as a team. So, we were basically teaching the same content to different sections. And the good thing is that in teaching face-to-face I was able to relate my experiences to the other tutors or the faculty to know how the content and to whatever else happened in the class. However, in online teaching, I realized I was just to myself. So, it was just like it’s me and that's it. I couldn't share things because obviously the other teachers did not share the content or did not teach the content. So, I felt just only to myself. That was my disadvantage.

**Interviewer: So, you mean to say less student engagement?**

**Interviewee**: No, between teacher's interaction. Between our teachers, the interaction.

**Interviewer: Okay.**

**Interviewee:** Yeah.

**Interviewer: Right**

**Interviewee:** As on how the particular content went and how it was and as to how the students react. So, I was not able to give and get any feedback from my tutors.

**Interviewer: Right, thank you for this, and also X. S said that it's the lack of interaction in online teaching and lack of motivation. The students are not prepared. There are some other points that are coming up, is there anything which is left? And which you want to discuss? Is there any other disadvantage? X. you were asking the question, is there anything which you wanted to say?**

**Interviewee:** Yeah.

**Interviewer: Yes, go ahead?**

**Interviewee:** One thing that I felt during my online teaching is that it gives an opportunity to students to skip very easily. If I am not hearing from one student and I just call or his name and I ask as please let me know what we have discussed. The person will respond that "Oh X, just now my connection got disconnected, so I didn't hear you." And things like that. So, I am not sure whether he was there or not. whether the person is saying correct, that the connection got disconnected or the person was not at all present there and was just making an excuse of this disconnection and things. We just cannot know at all how many students participated, and as how much did they participate. and as what quality of learning actually took place. This is very difficult and very challenging in the online mode.

**Interviewer: Right. Sir. would you like to add?**

**Interviewee:** Yes MsR, during my experience of online teaching I observed that the students who need more attention or the students who are slow learners, weren't identified in the beginning and due to that they weren't even able to pass the course so i believe that due to online teaching when we cannot actually see every student we cannot gauge or judge that as how they are participating and as to how they are learning. At that time the ones who are smart and who can learn on their own. They are able to pass the course. But the ones who need our attention and the ones who need more support, we are not able to identify them. I think that this is the biggest disadvantage of online teaching.

**Interviewer: Right. If we just summarize what we have discussed up till now it's time-consuming, it's lack of interaction and lack of motivation. The students X out from the classroom and it's difficult to grab them on the screen, we do not know whether they are lying or telling the truth about what is going on behind the screen. This is what we have discussed, also the interaction between the teachers was Xing, the feedback was Xing so these are the disadvantages of online teaching in the Pandemic situation.**

**Moving to the next question. This we have already covered but I just want to reiterate it, if there is anything left you can just add-on otherwise, we shall move to the next. In your opinion what are the competencies, skills a faculty member requires to teach online during a crisis? Earlier we discussed that teachers should be trained to handle different technological platforms like Zoom, like Microsoft Team, and also that the teachers should be able to use the various strategies in order to engage the students in the classroom. What else? Is there anything that you need to discuss further? Yes, MsM?**

**Interviewee:** As I have already mentioned, I mean I have written in the chat box, too. That along with the different software or the techniques of connecting with the students online or engaging them in a number of activities which are interactive in nature. We also need to be trained ourselves in developing online assessments. Because that is something which I really felt deeply during this Pandemic time when we were switched to the online modality of teaching completely and there was a time when I believed that the entire pressure somehow to some extent was on the blended team because there is a lot of technical working involved at the back end when we have to project something online towards students. When it's content-based we still somehow know how to make presentations and how to prepare voice-over presentations. Or how to prepare online quizzes and assignments and upload it on the BLE course pages but when it comes to online tests that are time-bound. Which have a certain number of questions involved which have to be transcript and then they have to be connected with a score and this and that. There are a whole lot of skills which are required to complete it. That is something which we need training in.

**Interviewer: Conducting online assessments or organizing how to go about it and...**

**Interviewee:** And developing them as well.

**Interviewer: Right. X has said that we need to reach in the assessment and make the content to interactive ones instead of uploading plain lectures. Right, this has been noted. Is there anything else and we are about to discuss regarding assessments and gradings so is there anything that is left, and you want to discuss?**

**Interviewee:** I just wanted to add one point that perhaps even as how we have struggled much on online teaching but lack of resources was a hindrance. I felt that sometimes that was an hinderance our planning of our clinical, especially. I am sorry I am talking about clinicals most of the time because I am a clinical teacher. When we are planning clinical at that time as in mental health. I am teaching Mental Health. Then Mental Health has a specific need of communication skills. This all depends on it, mental health completely and entirely depends on communication skills and for that when we were planning we had thought about the standardized patient interaction with the students most of the time but due to the university at crisis regarding financial issues and everything we have to limit our budget also. And according to that we need to plan so that the students cannot be suffered, and they can at least have few of the communication experiences with them so that they can have that exposure of having a patient feeling. So, I felt that was a challenge for us because due to this we had to plan again and again and we had to move back and forth. I am talking about the faculties and the group of our teachers who were planning, and we had to go back and forth for the resources, fight for the resources. And then plan accordingly. Planning takes a lot of time. I felt that I needed to share this.

**Interviewer: Right and clinical is something that is you can say that this course demands it. So a very important aspect of this particular course. X A has shared that at the moment the technology cannot be controlled but as time passes the technology can be controlled. Okay right**

**Interviewee:** I feel that there should be some kind of orientation for the students and the faculty for online teaching and learning. In the beginning only when we are starting the program and all.

**Interviewer: Yes.**

**Interviewee:** I am not talking about in terms of Pandemic. Let's say the Pandemic was one way of piloting online teaching and learning in all the courses. We did have blended courses previously, post RN but we ended our blended courses in online learning. maybe there can be some kind of orientation in which we can get training to the students as well as to the faculty.

**Interviewer: Right. Somebody has said. X A I think that it is in pipeline, so it is good to hear that. I think let’s move to the next question which is regarding the student assessments and grading. So, what are your experiences of student assessments and grading in complete online learning environment during Pandemic. I will paste the question over here so that you can read. Yes X.**

**Interviewee:** Again, the experience is varying depending upon the type of the assessments. If it is the paper pen assessment, the experience is that we need to accept the cushion of quality, compromise during the assessment. But if it is a presentation kind of an assessment or some paper submission kind of an assessment then yes online is okay, we don’t have any, at least I didn’t face any specific problem in it.

**Interviewer: Okay.**

**Interviewee:** But if it is in exam based where student has to sit and write at the same time, synchronous kind of a thing then there the definitely the quality is highly compromised. And we as a faculty tend not accept this kind of an exam.

**Interviewer: It differs from the type of the assessment that you are taking. Yes X.**

**Interviewee:** I just want to add that the trust relationship between our student and ourselves is not there. It is not there because this is the first time, they are writing over the book exam. And this is the first time they are not being monitored or proctored or invigilated so I see a completely different generation who has passed exams in professional schools and in academic institutions. I don't know what will be the level. This is what my fear is, this is what my experience about this assessment is that grading and assessment would matter most in the next fifteen years when this generation would enter the practical life. I know I am talking about totally different scenario. But as what has been said so far is there, it is the issue at present. But what I feel most with my experience of assessment that writing assessment is not an issue, giving assessment is not an issue. But how can we be, how can we be certain that whatever has been assessed will be utilized, will be retained by the students, and retained in the form that it is applicable when they would enter the practical field. So, a totally different perception of mine and I am sorry for that. But this is what I fear about giving assessment online and grading them. Everyone is getting a good grade, I never had a class that was A plus or A minus grades in it. In ERP and physiology which is a difficult subject for them in year one and I know people would agree with it but then this was there, so no control. Thank you.

**Interviewer: We respect your opinion X. and it is very important when it comes to implementation so yes and again, I repeat that this field or this degree requires or demands for, to implement whatever you have taught them. So, yes you are right. Let’s take response from X?**

**Interviewee:** Yes, X. you are very right actually. When I was assessing the poster presentation and poster presentation is like something which you present verbally and you explain what you have done and unfortunately when i saw a glimpse of the clipping, I saw that the students were reading from the book, or from the papers or the notes, whatever. So, you know that was not supposed to be so. This is one point which I just want to raise.

**Interviewer: Thank you.**

**Interviewee:** Yes, I want to add up the same that MM and MsN have mentioned. I would say we have made history in this Pandemic in having all A grades in not only in one course but in most of the courses. So, if we are having this kind of assessment, we should just pass the students they should not be graded.

**Interviewer: Okay. So, this is another thing that we have noted down. X. MsN thank you for your time if you are leaving in the next five minutes for another meeting. Just want to add if there is anything which you just want to share please write us the reflective vlog on the same ID from where you got the email for the Zoom link. So that it can help us in enriching the data. Thank you, X. MsN, once again. I think we have shared our experiences about assessments and grading. Sir. MM also agrees to this pass and fail grade point. So, thank you Sir. MM. Let's talk about what the teaching learning strategies that you prefer with on-line learning during Pandemic and why? So, you can talk about the teaching, learning strategies that you used in your classes and you preferred to have it in this online mode. X S do you want say anything about this?**

**Interviewee:** Well, I used a number of teaching learning strategies because I was trying my best to engage the students through online learning like as we all said that we feel that we are talking to a wall. So, I had used gaming, pair, share activities where they can interact more. I used Kahoot, which was very much appreciated by the students as well. I tried to engage them into small group discussions, break-out rooms and then large group discussions but as we have all mentioned that this student participation or student availability we did not know that the name was there but we did not know whether they were there or not. We tried our best and that they open up their video window, but they did not. Majority of them did not. But I tried most of these strategies with my students even role playing. Even what we did as MsRZ was talking about the clinicals, I would like to talk about the Labs. As my course is Lab-based course. It has got a component of Lab as well. So, I had to record the Labs, the experiments and the practicals and had to send to them. Even that was one of the strategies which I had used in their demonstrations and the students appreciated that as well.

**Interviewer: So, a lot of different strategies that you have discussed Kahoot and then role playing. Then think gestures and discussions. Right.**

**Interviewee:** Discussion Forum.

**Interviewer: Discussion forum right. So, yes, X.**

**Interviewee:** Thank you. I agree with X. S’s point that she has shared. We have also used almost the same but adding to that we have used standardized patient strategies which we called SP's. The standardized patients were actors actually and they have acted like a patient. And we have given training to them prior to conducting the activity. And that strategy we have used. A cyber patient is one of the apps which we have incorporated in our learning. The students learning. I and X have also worked on video recordings. We have priorly recorded two videos which we developed for the students learning on the communication skills, so we have our video recorded learning. We had used a theater-based learning for the students, especially for the mental health. Here we have given them a concept to clear their concepts. We gave two videos to them with different topics where they could learn about the mental health. One is schizophrenia and other is related to Bipolar and they have used that video in their own style, and they have learned. And we have given them a demo strategy where the faculty give a demo on the spot on how to communicate the sudden situations regarding if the patient has issues regarding the mental health. It was few of the strategies besides what we have used as strategies for the students, I would like to add that we have also learnt new strategies from the students as well. As when we have our post conferences and we consult with the students they have used innovative strategies to communicate their content so that we had learned a lot like during the art therapy, one of the students had introduced online art mandala activity which was new for all the faculty as well when we were conducting the post conferences. So, it’s not that matters as what we have used but what we have also learned from the students as well.

**Interviewer: Okay. So, demonstration technique and other than that as X. S said you used all of them and the other point that you have shared is that you people have learned from students as well a lot of different online techniques. Right. So, I just want to add, that X. XXX has said that Padlet, menti meter, quizzes all these are some more activities that I used as a teaching strategy in the online classes. Right. Would you like to talk about group activities, group work or peer work, break-out rooms. Anything that you experienced, or you wrote about it?**

**Interviewee:** Are you asking me?

**Interviewer: Anybody can answer, you can begin if you have anything to say.**

**Interviewee:** We had several group activities as well. Like as the clinical skills-based learning camp. And for the first time we had used online virtual field visit with us. Actually, in the mental health we always take the students to institutes that are ours in Malir. Where they get an exposure with the clients who are drug addicted. And we cater to them and learn how the institute is working with them. But this time we brought that physical visit as a virtual visit. So that was a new strategy that we have used for the learning because we could not leave that part as that was an important part. We had planned multiple mixed activities for the learning of the students.

**Interviewer: Right. So even X said that working with mix of the activities making the content more interesting and exciting. Is there anybody who would like to talk about group works or peer work and group activities. Or anything? Group assessments, have you ever given them group assessments? This question is for everybody. Anybody who would like to speak just raise your hand and answer. This can be told by anybody, you can talk about your course or anything. Yes, X you were saying something?**

**Interviewee:** This I wanted to share that we had a case-based study and that was a group activity actually because all faculties have conducted that case based study is an assessment for the clinical assessment as well as a strategy for students to learn more and that would be conducted as many activities in a group form specially with patients it was also in a group form where patients or standardized patients were online there and all students had come in their particular slot. All SP's had been given and trained under 4 different scenarios and the faculty prompted them a number of the scenarios. They are not told the topic, the student has to guess what the patient is actually suffering. We had to work hard to make the scenarios, but it was an effective activity in the group form. Two students were present at the same time and the rest of the students had to observe those students. And their observation was what they had learned especially in the mental health communication skills and then these were conducted back to the students and then the students had highlighted that this activity was very affective for them to have a learning online in spite of going clinical face-to-face.

**Interviewer: Thank you. X for sharing this point. Yes, X?**

**Interviewee:** I was saying that formatting evaluation was very important and was very good. And i was saying that I had used break-out rooms so in these break-out rooms i had divided them into small groups then when they came to a large group one of them was supposed to present what they have discussed in the breakout rooms and one thing which I felt was very important was ongoing feedback. I feel that ongoing feedback was a very essential component for this effective learning environment especially on the online which we had done. And as an online teacher my feedback helped the students to have their e-learning experience more informative and I was able to make them more engaged and motivated. And I also felt that the feedback I used to give them when they used to come from the break-out room or the larger groups and what they were presenting. This e-learning process was very constructive and the feedback which they were using. and then they were utilizing that feedback later on in the other activities as well. So, I feel that feedback was very important and then as I mentioned about Kahoot. That Kahoot is one of the strategies in which they connect through the mobile so making e-learning contain through mobile and the faculty on the computer and they are on the mobile and that was very much acknowledged also by the students. And it was very important as how they use mobile learning for online teaching. That was also very much appreciated by the students.

**Interviewer: Okay. As you discussed X S that the break-out rooms that you divided the students into groups and then you gave them the tasks. I just want to ask that were you satisfied with all students, were they participating equally?**

**Interviewee:** No, I wouldn't say that everybody was participating that some of the areas which we have discussed earlier that student participation is the biggest challenge in the online teaching and learning. That is how to engage them and how to motivate them so that they actively participate. All of them did not participate but I used to try my best to involve them in the large group in discussion to make it more interactive and so on.

**Interviewer: Thank you for sharing this concern. Moving to the next question and it is related to the challenges. What are the challenges that you are facing in online teaching, some of you have already discussed about internet connective tissues, and using of various platforms, so I would like to ask you about some more things that left to discuss. So, it is not necessary to repeat those points. If there is anything left which you feel is a challenge for you then kindly discuss that. I shall repeat my question that what are the challenges that you are facing in online teaching? Anybody who would like to go first?**

**Interviewee:** I think we have already discussed this.

**Interviewer: Yeah, we have discussed it. Okay, so I think we are left with three more questions, so the next question is what are the views regarding the online teaching and learning for the students residing in remote areas? Would you like to talk about the students who are residing in the remote areas? Any challenges that you faced or any experiences? Yes, Sir. MM?**

**Interviewee:** There was a very big challenge actually in the spring semester. The students were back in their hometowns and there were one or two students who really had a problem because there was no internet connection. They were living somewhere very remote. And just to get the information or just to get the content matter, they had to travel three or four hours to a certain school, and they had to come in that certain time where the school was open and where the internet was on also and then to go back.

So that was a very big challenge for some of the students. And the students who are staying at very remote places, for them that was a challenge.

**Interviewer: Travelling and then moving to that place where they could get internet available was a challenge.**

**Interviewee:** Yes, there was one instance where when the student had arrived at the school the school was over and the students had left and that student had to sit with the guard who was there and he had been kind enough to let her use the connection. So, I felt very bad for her.

**Interviewer: Okay. Thank you, Sir. for sharing this. Yes, X.?**

**Interviewee:** Adding to Ms point, yes the student had that issue of going to the far areas for the connectivity of the internet but when they reached few students had shared they consulted to us that when they reached at the school from where they had been facilitated the connectivity, they had an older version of technology which they were using, the windows software and due to that they could not upload the content of the teaching from that particular software. Let’s suppose they had window 7 which they had been using and if they did not have Windows 10 the content which was needed could not be downloaded. They could just be able to log in, but they could not download it, so that was the biggest challenge which the students have shared from the remote areas. And there had been safety issue as well. They could not move alone, they needed to have some male family member with them available to bring them to that particular area where they could have an access on the internet where they had been assigned a school to have the connectivity.

**Interviewer: So, security is also a concern for them, and X. A has added that older version of software’s that may have caused the hindrance to their routine. Yes, X. S?**

**Interviewee:** As A has just pointed out older version of software. Like when I was doing some workshop training and I found tools and I started using those tools in my teaching and learning. And then I found that they have to be purchases. So, we don't get most of the instructions in it because those tools can only be purchased and then only can they be utilized. So, in future if we are going to go online more, we I think that as I said in the beginning institutional support is the biggest support. Alright? So, the institution should buy those tools for us. Like Padlet. There are a few things which are free in the padlet. But there are certain things which we want to utilize but they are not free. Even in the Kahoot the Kahoot is free to some extent but not too much extent. So, these are some of the online tools which we would like to use in our online courses and in our online learning. They should be purchased. for any extent of effectiveness.

**Interviewer: Thank you X S for discussing a very important thing and i noted it down in the recommendations.**

**Interviewee:** Coming back to the question which was about students accessing the teaching learning material from the remote regions? Yes.

**Interviewer: Yeah.**

**Interviewee:** I think something that MM mentioned was hundred percent true and I also heard some of such stories from my students when they returned. After having sometime spent on remote learning, they shared with us as how difficult it was for them. And I think on one hand where they were investing into those assigned schools frequently it was also putting additional financial burden on them. Because they did not have personal conveyance and the majority of our students, the economic background to which they belong to they hardly have their own vehicle or any automobile machine. So, it was seriously difficult for them to take a public transport and get one hired for them to reach to those places and the frequency of commuting was also there. So, I believe that it must have put some additional financial burden on them.

**Interviewer: So, it was a challenge for them to just reach the specific place. And then yes Sir. MM has also agreed to your point. So, now I think X would like to share something. Is she still connected? Yes ?**

**Interviewee:** Yes, I am online I had lost my connectivity, so I Xed one or two points. I have just heard a few of the last points that the financial burden was a big issue for the students as well. Yes, in the Northern areas the transport is too expensive. If they have to go five miles even from their home, they had to pay around for one trip 5000 to 8000 per trip. So that was a big burden for them as well. Because I had to go to Chitral, and I had experienced the same. The financial expenses are very much for the students.

Am I audible?

**Interviewer: Yeah, you are audible. This is what X has shared that it has put extra financial burden on the students living in remote areas. So, I think let's move to the next question and then we are left with the last two questions, so the second last question is what are your views regarding the complete shift or the complete migration to online modality? If there is any fear or anxiety. would you like to talk about it? What are your views about the complete shift or migration to online modality?**

**Interviewee:** I think again, the point is which MsN had initially shared and that S had shared that we would require a complete training if we would like to go completely online. We all, the faculty along with the students. We attempt a complete training regarding all as how to access the different tools and how to have accessibility of connectivity. We should have all these. So, we need to make better all these things if plan to go for complete online.

**Interviewer: Thank You.**

**Interviewee:** MsR I should ask that question to you because you have listened to our conversations and our discussions and all the questions which you have put up to us we have answered, so what do you think are we ready?

**Interviewer: I think you people have mentioned in the beginning that it was difficult for you to juggle with the things and all. But it's time I think to inquire from you about your views. Because if I will give my response them it might give a biased view.**

**Interviewee:** Just to conclude I would say from all the discussions which we have been having since 10 o' clock. The success factors of online teaching and learning is a number one institutional support. So training, tools software’s. Then I talked about the curriculum. The curriculum development course structure students support, the faculty support evaluations and assessments. and as how to do that online and how authentic they are and the we have talked about the technology control for the faculty as well as all the students. Motivational competency of the patient, competency of the of the students and the learning material accessibility. Computer availability. Instructional design. And then so forth. So, we need all these things which we have discussed and if these are done then I think we are ready to go and pursue online.

**Interviewer: Thank you X. S. Is there anything else that you would like to share? Any other person? Or even you can write it in the chat-box. Anything that you think as if you are ready for it because X. S has concluded it so well. I think let's move to the last question and few of the recommendations you have already given. So, what are your recommendations to improve the online teaching and learning? So, let me just summarize what you have recommended uptil now. You have recommended training sessions for the teachers and also for the students then just before this question you discussed about the software’s that the institution can buy for you in order to help you out in teaching in a better way and effectively like you said Kahoot and then there are some features in Padlet too. So, any other recommendations which you would like to mention? Yes anyone? Okay Sir. MM said that we need to expend our technological staff for assistance, okay, thank you Sir. MM. Yes X. S?**

**Interviewee:** I mentioned this earlier, I would like to build on it. We need online teaching and learning centres, small centres in which they can cater to all these needs of ours, like even if we are doing a recording. We do not have any separate space for the recording. We do it, where the faculty sits or in their own offices they are recording the lectures and the presentations for the students making the podcast, Yes we have one person for that, but if we are thinking for the future online program then these are some of the very important things, I would say that these are the pillars of online. So, we should have a teaching learning centre online. It should be something where the teachers are planning, when they are recording the lectures, when they are preparing their presentations they can go to those areas and sit there quietly with no sound problem and that area is specifically for online training. So, we need something of that sort.

**Interviewer: Thank you for this recommendation. So, yes Sir. MM also agreed that we need online centres and if we have any problem, we can take the assistance from them.**

**Right Sir. MM. So, is there anything left that you need to discuss? Otherwise we are about to end our discussion. So, is there anything?**

**Interviewee:** How much time are you going to take analyze your result and we get to know your findings?

**Interviewer: I will let you know about it.**

**(Disturbance in recording)**

**Interviewer 2: We will let you know perhaps in a months’ time it should be ready with an inhouse circulation of some kind and then will share some results of the interviews. Thank you.**

**Interviewer: I think that’s all from our side. And thank you for taking out time for this interview and the last thing is that if there is anything that you want to discuss or share you can always write in the reflective log. I have posted the email address in the chat -box. So please not it down. And if there is anything you can discuss it in a written form. So, thank you once again. And Allah Hafiz. Take care.**

**Interviewee:** Thank you very much.

**Interviewer: You are welcome and take care.**

**Interviewee:** Good Luck.

**Interviewer: Thank You.**

All respondents say Allah hafiz.
