# Supplementary material for: Beyond the classroom walls: Stakeholder experiences with remote instruction in Post RN baccalaureate nursing program during the COVID-19 pandemic: A qualitative inquiry
Source: PLoS One. 2024 Apr 4;19(4):e0300007. doi: 10.1371/journal.pone.0300007 (PMC10994296; doi:10.1371/journal.pone.0300007)
Supplement: S3 File — (DOCX) [file pone.0300007.s003.docx]

**IIKI 1 – January 5, 2021**

**Interviewer – Ms. 1**

**Interviewee- Ms. 2**

**Speaker 1:**

Thank you so much for your consent. Might be one more member will join us for note taking. So if you see any other person in the group that will be Ms. Zeenar, and she will be part of this interview, as a note-taker. So I am just informing you beforehand. So are there any questions that you want to ask,

**Speaker 2:**

No, please proceed.

**Speaker 1:**

Okay. so my first question is what are your views about online teaching learning?

**Speaker 2:**

So, I have been associated with e-learning field for the past 14 years. And I have been you know working for international clients and you know multinational companies and I've been really fascinated by the way they have taught or by the way they are teaching and promoting their education. So if you ask me, I've been always thinking about how we can integrate this online learning and how we can promote this online learning here in Pakistan. The sad part is that it actually started in you know, such misery because due to this pandemic, we all shifted or moved towards this online learning. But I always wanted to promote them in a good way or in a very friendly environment where we can teach and promote this online learning. But yes I am hundred percent supportive towards this online learning.

**Speaker 1:**

Okay. Good to hear that you are working in this particular field from the last many years, and you are still working though. We got this opportunity. We can say that this COVID-19 has become a window for opportunity for us. Though, It's a big issue, but has become an opportunity for us. So going to the next question, what are your experiences of supporting the migration of existing curriculum to remote teaching and learning during the pandemic? How can faculty and students be better supported in this transition?

**Speaker 2:**

So basically the thing is that when I started working at AKU, we wanted to move all the content that is not available online. We wanted to create online courses to support our students in every way possible, because, you know, teaching online is one of the necessities these days. There are a lot of people who can not afford their own learning or their own education. So they are usually working somewhere and during morning hours and they want to have something that whenever they are free from their working hours, they can get the education from somewhere. So, yes. Over here, it is a bit difficult to convince the teachers, the faculty, the students as well, but this pandemic again. As we have discussed earlier as well that this pandemic we can say that it's a blessing in a way that everyone started believing in this online learning it, everyone started you know, forcing themselves to move towards online learning. So, again it has been a very you know roller coaster ride because when the pandemic started, I was along with one of my departmental resources, we were on our toes 24 seven. We had been receiving calls from the students. We have been receiving calls from the security, our seniors, everyone, because, you know, everyone wanted to know more and more. And since there were you know the internet connectivity issue, it's not available very easily in urban area, so we need to facilitate them as well, we have to, or we used to, you know, provide them solutions that if they are not, you know if internet is not accessible through LAN or maybe wifi may be, they can try using the mobile data package. If the signals are there, because usually they are using WhatsApp to communicate. Right. So this is one of the things that, these are a few things that were problematic for us, but then again, if you ask me, I personally enjoy helping the students and the faculty, because this is one of my dreams and I, you know, want to promote online learning in Pakistan.

**Speaker 1:**

That's good to hear. I just want to repeat the question as you discuss that, like students faced internet connectivity issues, and those who are in remote areas, they might face more difficulty due to internet connectivity. So you, people are already supporting no doubt, but are there any other plans to better support in this transition? Because we don't know whether we will be continuing with this problem for the next year also. So are there any further plans that you people are planning to have it?

**Speaker 2:**

Yeah, so basically, besides the internet problem and internet issues, we have been providing them the material by USBs and you know we have been providing them all the online material converting them into files, the reading material, sort of things in the PowerPoint or the videos and then sending the work to the students. Because then again, when I said that there were internet issues as well. And then when I say internet issues at times as 3g was not also working, so these are, USB's were very helpful for them. So, since phase one students also realize that how much the internet is important for them as well. So they are also trying their level best to acquire the internet access on their own. And, we have been providing solutions for these things as well, and this USB thing was one of the solutions that we have been providing to the students from day one.

**Speaker 1:**

Okay. So, when we talk about online teaching, there are some advantages and there are some disadvantages. So I would like you to first talk about, some of the advantages of online teaching.

**Speaker 2:**

So, like I mentioned, online teaching is one of the blessings because, you know, the students are the people who are working in days or during daytime. They can take the online courses whenever they have time, whether on weekends or whether online during the night time as well. For me, if you ask me, I have taken a course as well, and since it was from a foreign university, so you know, the classes were in the night, so everyone was sleeping at my home and I was taking the classes. But then again, at the end of the day, I was really content because I have acquired some information and some knowledge through those courses. So this is one of the, , one of the best examples, or you can say that the advantage of online learning.

**Speaker 2:**

Plus, like I said, that I was in Pakistan and I took the course from one of the foreign universities. So I didn't have to travel from one country to another. I didn't have to bear extra expenditure because of moving from one place to another, because whenever you go outside, you need a place to live. You need food to .. you know.. And everything. So this is one another, another thing, that you can do. Disadvantages can be one of the main, , or the biggest disadvantages over here at, since we are talking about Pakistan, is that internet connectivity issue, because not everywhere, like I said, there are certain remote areas where internet connection is one of the biggest challenges for us. Besides that there were some challenges just like electricity, power failure, and not everyone has generated backup plans to, you know, connect to the internet or to the electricity.

**Speaker 2:**

But then again, one of the advantages is that the courses that we are offering, you can access it through your mobile phone as well. So if your mobile phone is charged. And, , if you can, you know, log in to disconnect to the internet log into that particular website or the VLE, and then just download the material and then just disconnecting, you can go through that PowerPoint or the word docents even when you are offline are not connected to the internet. You can read it on your mobile phone. So, if you asked me then again, since I am a supporter of online learning, I have more advantages rather than disadvantages.

**Speaker 1:**

Okay. So I will just repeat what you have said. You talk about travel expenses, which, saved because of online teaching. Then you talk about, a student or anyone can do it at their own pace, because the availability of material online at any time or at any place. Secondly, you talk about the devices that can be accessible from a mobile phone, or even any time whenever you want to, or you download it. And even if the internet is not working, you have the material to study. Is there any other advantage that you come up with?

**Speaker 2:**

There are a lot of advantages, you know, it's cost saving as well, as I've mentioned, you know expenditure cutting ...cost saving? , it saves your travel cost as well. , it's accessible anytime, any place you can just connect it and you can go through it. I guess that these are the most important things that one should consider, because these are the main things that we consider while pursuing higher education.

**Speaker 1:**

Right. So let's move to the disadvantages as you discuss about the internet connectivity issues, or electricity, or power failure, any other thought or any disadvantage like time consumption or balance with work and family, would you like to talk about these aspects?

**Speaker 2:**

So, I guess this pandemic, when this situation started for me, since we are talking about something that we have faced right. So if you asked me, so, 2019, when this pandemic started, you know, I was literally one of those people who suffered a lot because of the time constraint, no matter what time is it, I used to get calls from the students or the faculty as well, that they are facing this issue. Till September I've been on my toes, 24 seven, and resolving students' problems and teachers' problems on and off. , but then again, if you ask me, I guess, , you know, it's very, , since it was the new thing, a change. Whenever there is a change, everyone don't take it as, as a positive thing, right?

**Speaker 2:**

There's a lot of pushback to it, but if you don't have a choice, you have to accept the change. So, for example, I want to give an example of my five-year-old, she's also taking online classes, right? I don't find any problem in her or any issue in her. She is mashallah learning, she's participating in the class as well, whatever the activities as well, they are conducting, even when she's having her PE class, she's having her music class. She's having her art class besides all the compulsory subjects. She is having all other classes as well online and she's really enjoying that. So, yes, she misses going to school, but then again, she has everything for half of the day online right there. So, I guess when she's a kid, if she's just a kid she's just a five-year old, if she can accommodate or accept the change, I guess everyone can accept the change. Like, and considering the fact that, you know, you don't have a choice. So the older you are, you can, you know, write down your pros and cons of the situation that you are going through. And then you can, you know, we take out the important things and the less important things, and you can proceed with the changes in your life because these kids don't understand what we are going through. Right, what the dynamic is really. So if they aren't accepting this change, I guess everyone can accept it.

**Speaker 1:**

Right. So a very good point to note that, if something is there, it's better to accept it rather than just panicking and avoiding the situation. So moving on to the next question we talked about how you people are supporting and all, but what do you think, and what is your opinion on what are the competencies or skills, faculty members required to teach online during the crisis?

**Speaker 2:**

So, basically, these faculty are usually most of them at SONAM, most of the faculties I have been teaching online, they have been conducting classes online, and I think they find it very interesting as well, because there are few figures. Those who are not even here in Pakistan and they're conducting their classes online. Right? So, even the students are also taking the classes online. We have been, you know, the material is online for students and we have been promoting them before the pandemic too, or, you know, take the classes or at least go to VLE. The reading material is there even with the faculty, they are forcing students to go to the VLE and read the material online because, you know, this is maybe on the next day. And if, if this pandemic would have written, have been here maybe next 10 year we would have been, you know, using the online platform because, if the world is moving towards online education, then you also have to move towards the change or the online learning platform.

**Speaker 2:**

So I guess we are also doing good. Yes, there are a few things that we need to get hands-on experience. Like for example, they need to know the technicalities. For example, if they, if we talk about the zoom class, right. They should know each and every feature of the zoom, like how the class would be, how we can distribute the classes into smaller classrooms, how we can bring the students from different classrooms into one classroom. Similarly how we can use the whiteboard on the zoom. So if we talk about the Microsoft team then we should get a hands on it and MS team as well. So these are the few things that even if we are not there to support them, because you know a lot of faculties are there. and there are only two support people at SONAM. And at times, we both are occupied with someone else, and there are few faculties who wouldn't get a chance and we are, you know, rushing from one place to another to support them. So for such cases, I guess training would be delivered more and they can learn more and they can conduct classes on their own without our support and help.

**Speaker 1:**

Okay. So, what I gathered from your point is that almost all of them were already teaching in this online mode earlier, and the rest of them have learned with the time, how to operate zoom or how to use various features of zoom and MS Team. So these were the few basic skills that a faculty member required to teach online during the crisis. Right. So

**Speaker 2:**

If there are more difficulties, like I mentioned, there are few faculty facing difficulty again. We are there to support them, to guide them, and it's not something difficult to learn.

**Speaker 1:**

Okay. So, it's very important to engage students during an online learning environment. So in your view, how can a faculty effectively engage students in the online learning environment?

**Speaker 2:**

So, you know, I also teach at SONAM. And, if you ask me, for example, last two weeks before I was teaching them about Ms. Word, and what I was telling them was, I was actually asking them questions, right? Because you know Ms. Word is a very basic docent that everyone knows about. Right, but it's so very , you know, , a very detailed document, not everyone knows about all the features. So the basic features, everyone was raising hands and participating. For example, if I ask them where you can, you know, align something, so they weren't raising hands and participating in it. So when I started going into deeper features, they were not quite at that time because, you know, I started, or I initiated with asking very general questions in which everyone was very excited to answer. And then when I moved towards and I appreciated them. And when I moved towards, you know, asking complex questions, they were encouraged to ask them whether right or wrong. So, these are the things, you know, you can play games with them online, like, you know, just a refresher game, maybe we can use Kahoot and we can ask them certain questions, brain teasing questions. And these are the few things you can engage, your classmates, even this is we are talking about online class, right? Even when we are in a physical classroom, we usually do such activities because we want our students to be engaged and very responsive. , so that we also know that they are learning and they are getting, you know, the information that we are trying to pass it on to them.

**Speaker 1:**

So, summarizing it, the features of zoom itself gives us an opportunity to engage students and as well as online games, which are available or different educational tools, which are available as you mentioned, Kahoot, or some brain teasing question, and there are some more that can be used effectively to engage students during the online class. So, moving to the students who are residing in remote areas, I just want to ask you that, what are your views regarding use of online teaching and learning for the students who are residing in remote areas or different parts of the country?

**Speaker 2:**

So,

**Speaker 1:**

You discussed this earlier, if you want to elaborate on this a bit more, it would be great.

**Speaker 2:**

The only thing is, that, if there's a will, there is a way right? , like I mentioned, I'm a working woman. I'm actually a working mother.. I have a toddler in the house. I have to take care of that kid as well. I have a nine to five job, which is not actually nine to five. Since I am, you know, a technical assistant as well. So I usually receive calls or messages in my after shifts as well. So I, then I have to take care of my home and besides all of this, I am there to get more education, right. So I am willing to learn more, I can find ways to learn and to do things. so for the remote area students, like you mentioned they were also very eager to learn more and more. And when we receive calls from them, they are really very, you know, , enthusiastic to know more about it, for example, if the internet facility is not there. They used to ask us if there is any other option they can use a mobile, or you know, we can access courses from, and we used to tell them, for example, yes, go and connect your 3g from there, download the course material, and then just disconnect. Right. So that your package doesn't expire and save your cost. And then you can read it online as well. Right? So, this is how we can motivate them. And then again, when I said, there is a will, there is a way these students are eager to learn and they, if you just push them a little bit, they just, they are willing to do everything.

**Speaker 1:**

So, you have provided all the facilities to them, and now it's on them, how to use them effectively. So moving to another thing is, what are your views regarding complete shift or complete migration to online modality? Like, are we all prepared or do we have fear or anxiety, would you like to talk about it?

**Speaker 2:**

See, I mentioned it earlier as well there, whenever there is a change, not everyone is willing to accept it. Right. Obviously everyone has to accept it because the changes are already there and there are people who accept them unwillingly. So yes, we have been online completely. We have been you know supporting the staff, the teacher, the faculty, and the students and, as well as, you know, there are a few things for example there are certain courses which include both theory and, labs, right? So the theory is there online, for the lab we are making certain videos as well because videos take them. Right. And we also conducted online labs where the students sit at their home and see what the demonstration is done by the teacher at the hospitals.

**Speaker 2:**

So they are in the labs. So this is there as well. , but, , if we talk about certain areas, like, for example, if we talk about the nursing school, or if we talk about the medical school, , theory needs to be done, or the hands-on practice needs to be done at the school. Right. So, shifting content online is doable. But then again, even if we can make videos online and for the labs and everything, but then again, they need hands-on experience and for that they have to come to the hospital. So I agree a hundred percent from moving the content and making the videos and uploading them. But yes, I agree as well that the students need to come to the hospital for the labs

**Speaker 1:**

So you mean to say that the nature of their degree or the nature of their course demands for some hands-on practice. So no doubt that the preparedness is there and the readiness is there, according to the course content and from the faculty and the student side, but the course demands somewhat hands-on practice. So it must be a 50, 50 ratio of both of the things like face-to-face and online modality,

**Speaker 2:**

Not 50 50. Oh, yes. We can say that 60, 40, 60, online and 40 labs. it depends. It varies from course to course. So if you talk about the courses that we are teaching at SONAM the nursing courses, so the ratio should be 60, 40. 60 can go online but 40 needs to be done on school, on board.

**Speaker 1:**

Okay. So, , now, , another aspect of this interview is regarding students' assessments and grading criteria. So I want to ask you, what are your experiences of supporting the planning of students' assessments and grading in a complete, online learning environment during pandemic?

**Speaker 2:**

So, we have been conducting exams online before the pandemic. So, our faculty is very much aware of this online. You know, taking exams and assessments, and, there were a few things that we needed to fix, before fully going online for the assessment. And we have already fixed it before the pandemic. So, right now, for the past two semesters, we have been taking all the exams online before that we were taking on online exams as well. But just to make this confirm that in this pandemic two semesters has passed and all the papers were taking online, the assessments were taking online, that result was announced online. So since we were used to doing that, we didn't face any problem with it.

**Speaker 1:**

Okay. So, any issues from students or faculty side?

**Speaker 2:**

No, because students were also, you know, , they were used to taking exams online, so they didn't find anything. The only difference was when they used to take online exams then they usually take it in LRC at Aga Khan campus. And there were a few people to assist them or support them physically. But at this moment there was no physical presence for them, but we were there to support them through WhatsApp and everything. But then again we didn't find any such issues which can be you know recorded away here. A few shows that are very basic or, you know, ignorable is that, you know, when the student started taking exam, some of them, they get scared or, you know, ,

**Speaker 1:**

Anxiety?

**Speaker 2:**

Anxiety you can say, so they couldn't find the button where to start the exam. Let's say, for example, a class of 146 students, only two of them reported such things we used to tell them, okay, refresh the page. And as soon as they refresh the page, the button starts appearing into the bottom of the screen. So these are few other things which are not even noticeable, or we can say we simply ignore them.

**Speaker 1:**

Okay. So good to hear that it went smooth. And even from both the sides, right the faculty and students, they did a good job. So next to moving to the challenges, , what are the challenges you are facing, or the team is facing in implementing online teaching?

**Speaker 2:**

So this is if you asked me, , that I am facing as a technical person, is that I am short on resources. We are only two people. And at times, if I am occupied with one of the faculty and my other resource, she's also occupied with someone else especially during the exam time, you can say shortage of a technical person in our department.

**Speaker 1:**

You mean to say human resource.

**Speaker 2:**

Yeah. So this is only one of the things at my end, other than that, because then there's this time constraint as well. Then, at times, like for example, my resource, she was on leave for the last 20 days. And I literally, I didn't find you know I couldn't manage my personal time, my professional time one, and I used to work 24 seven in these 20, 25 days. So yes, these are few of the challenges technically but besides this, as I've mentioned that we have been already implementing, , online learning on taking exams for the past few years. So I don't think that there is a major challenge that needs to be addressed.

**Speaker 1:**

Okay. So, we are left with few more questions only. So now coming to the recommendations, , what are your recommendations to ensure sustainable remote teaching and learning in the future?

**Speaker 2:**

See everything needs to get developed or get better with the passage of time. , obviously for now we are using this VLE platform. We have been integrating more features, or we have been using more features into VLE the model you can say. And I guess in the future as well, as we are completely shifting online too, I guess we will be exploring and introducing more features into that. But then again, as I mentioned that since the students are fully aware of this online teaching and learning, I don't think that it would be challenging for them and these young bloods, they accept challenges very easily and they don't find any problem with it. The problem arises, with experienced people. But, I guess, we are lucky enough to get the support from time to time and people start learning and implementing things from time to time and then do the same in the future inshallah as well. Hope, hope for that.

**Speaker 1:**

So, even earlier you talk about the resources, I just want to ask you, besides human resources whatever the other resources are they available for faculty and or the students or the staff members, or any recommendations for that?

**Speaker 2:**

I think it is accessible for everyone. And if we plan on bringing our students on board in SONAM , I guess we need more computers, , or, , proper computer lab for them so that everyone can use that. This is one of the major things that we are lacking at SONAM although there is a VLE, , there is an audio visual library, , at main AKU, then again, everyone uses it. So if we talk about our students using it, we need to improve that. But besides that, mostly students have their own laptop and their mobile phones and they can usually access it very easily.

**Speaker 1:**

Okay. So, now when it comes to a university's role, , how do you see university support or role in executing remote teaching and learning programs?

**Speaker 2:**

I didn't actually get them. Can you repeat that?

**Speaker 1:**

yeah, how do you see university support and executing teaching and learning programs?

**Speaker 2:**

Usually you know, whatever we do we do with the support of the university and everyone, and, , by the time, even before the pandemic, we were encouraged to move to online learning and teaching. And, obviously during the pandemic, it was majorly forced to move toward online learning and teaching. So all of this is done by the support of a university and, , you know, everyone because otherwise nobody would be implementing or using it. And they all will be facing challenges. If it wasn't forced from upwards. So yes I guess we usually have the support from the university and the upper management for this online teaching and learning.

**Speaker 1:**

Okay. So coming to the last question and that's regarding, SONAM... How do you think SONAM can be a trendsetter or a role model in introducing remote learning programs to meet countries nurses demands in the healthcare system?

**Speaker 2:**

So basically SONAM is a trend setter for this, online teaching and learning how, because SONAM started this online platform using an online platform in, I guess 2015 and nobody at that time uses this online platform. And we were the one who experience and experimented this model and then it was started implementing everywhere then again you know, we are ahead of everyone, in the sense that our courses are on level two and level three, there are certain levels of courses. Our courses are on level two and level three. And majority of the university courses, which include all the locations, they are on level one. So if we particularly talk about SONAM so SONAM is a trend setter and we are ahead of other departments,

**Speaker 1:**

Okay, so it's good to hear that it's already a trendsetter and a role model for others, and that it is already a step ahead and inshallah, will be improving more in order to provide better facilities for new badges and the faculty members. So I think that's all from my side, if you would like to add anything which you think you missed, or you would like to share, is there anything left out?

**Speaker 2:**

No, I guess I have shared everything that I have. And it was nice talking to you. The questions were really nice and interesting, and thank you very much for this opportunity.

**Speaker 1:**

Thank you for taking out time for this meeting and these ideas are very important for us. So, , and it will be kept confidential, like will be part of the study only. So thanks once again, and nice talking to you.

**Speaker 2:**

Same here. Same here. Take care, Thank you.

**Speaker 1:**

You too. Allah Hafiz

**IIKI 2 – January 11, 2021**

**Interviewer – Ms. 1**

**Interviewee- Ms. 2**

**Speaker 1:**

Okay. So my first question is, what are your views about online teaching learning?

**Speaker 2:**

So online teaching and learning was perceived to be difficult before the COVID-19. And there were opportunities for online teaching and learning on the campus. The QTLNet was doing a lot of capacity building workshops in order to train the faculty members for online teaching and in their bootcamp. And they were basically aiming to train our faculty to equip with new technology to use the new technology which is associated with teaching and learning in their classes. But it was observed that most of the faculty members were also not accustomed with the new technology. And we were, all of us were actually dependent on the traditional methods of teaching and learning. So no one has thought about that actually whatever we are doing face to face right now can be done online as well. So when you talk about online teaching and learning, I would say that it was perceived to be difficult. People used to think that it is not my cup of tea or, people used to think, okay, we are out of it. I mean, we don't have to do it because we are blessed that our students are there face to face, but when COVID came, it became a necessity, it became a must do thing that everybody has to do. So at that time, many of us were not confident that how it will happen. So this was a challenge when, when we talk about online teaching and learning. So, whenever the term online comes so, it will be a difficult thing, but now with time, people are adjusting with this. And, I must say that university has tried to give a lot of support in order to make a strain and make us comfortable in using and teaching online.

**Speaker 1:**

All right. So as you shared that earlier, it was assumed that it might not be required or it is always seen as a difficult thing to do, but after COVID-19 it has become a necessity. So my next question is that, what are your experiences of supporting the migration of existing curriculum to remote teaching and learning during the pandemic, and also how can faculty and students be better supported in this transition? So you can first share your experiences, and then we can switch to how can you better support this transition.

**Speaker 2:**

Okay. So the first question you asked that what was my experience regarding online migration? Right,

**Speaker 1:**

Right. Okay. Existing curriculum to remote teaching. Yes.

**Speaker 2:**

So, actually I am representing the curriculum committee at SONAM what happened that as the COVID started, our students were sent back to their homes and they were living in remote areas across Pakistan, almost 60% of those students were not accessible to us. And they were in the mountainous region and remote regions where internet connectivity was not there even some of them were not able to connect through WhatsApp or Microsoft teams which can easily be used on 2G as well. But our students, some of them belong to such a disadvantaged group that such a simple 2G device with the connection was not available to them. So what university did was that, first priority became, we were in the middle of the semester, right? So there were plenty of assessments that were remaining and the curriculum also had to be taught. So what the university did was that they tried to establish the hub and spoke model, in those regions where connectivity could be established with the help of the Aga Khan education services schools. So one thing which the university tried to do, and students were asked to access the internet through those hubs which were AKESPs schools. So one thing was taken care of like this now, but with regard to the curriculum. So being here, our responsibility was to migrate everything, including the teaching, as well as the assessments to the online modality. So that was quite when it came to us, it was quite hazy. We were also not familiar with that, how this transition will happen. So what we did was that we connected with the QTL net first. And then we try to understand that how, and what are the best practices for online assessments and online teaching. So that meetings, a couple of meetings took place. And then we come up with our faculty needs assessment and their apprehensions for online migration. So we conducted a need assessment first in which we received the responses that majority of them were apprehensive with the online migration. And most of them were fearful that probably they are not having the skill and not the appropriate devices to actually move in the new direction. So that those were fears at that time. and this was conducted in August 2020 at that time, that quick survey gave us an idea that majority of them felt that they were fearful, but almost 40 to 50% were also thinking that it is a way of opportunity. And we should, it is now on all of us to acquire new skills and to become accustomed with a new learning and cope with this, because this has to be done. So, we also found that they had, they were positive to learn, and that actually, their positive attitude actually helped us to conduct an online assessment, a workshop. So with the help of QTL net, we designed an online assessment workshop for the entire faculty, SONAM. And then that, what we did was that we had invited several facilitators. One of a few of them were also from East Africa. And one of them was our visiting professor from university of Saskatchewan. And we had facilitators from QTL net and our SONAM facilitators. So it was a multi disciplinary sort of a group that was conducting this workshop. And then we delivered this workshop in order to sensitize and to share the best practices for online assessments. So this was a readiness exercise and preparation exercise for the entire faculty members. And then we also received a lot of feedback on this step that what were the fears and now how they are feeling about their own competency and skill regarding the online assessments. Then we give time to the faculty members and then the revised guidelines for the assessment and planning in line with the HEC guidelines. So we also looked at the HEC guideline and we also looked at the best international best practices guideline that can actually help our context, which was no bandwidth and poor connections. So that could take care of both the things. And then we developed a new guideline and we gave them the new guidelines set to all the faculty members to revise their assessment plans in the current situation. So the faculty members revised it and they brought to us, they submitted the revised assessment to us. And then in the curriculum committee, based on their judgment, based on our context student need and students access, we gave approvals to the modified assessment plan. Great. So this was the entire story that how we migrated what principles we thought about. and definitely in our mind there was always the principle of access. equity and quality. We were completely thinking about all these principles. We have never tried to compromise on any of these principles while we we were doing the online migration.

**Speaker 1:**

Right. So, like the workshops you did with the help of QTL net and, and other like departments in order to support your faculty members and students, is there anything in your mind or within your team that is going on that, how will you be better supporting for suppose if this pandemic remains that like, unusual circumstances remains like this, and we need to be still teaching students online, then how will you be better supporting your faculty members and students, especially those students who are living in remote area, as you discussed in the beginning that I think more than half of the class or 60% of them are in the remote areas where internet issues are there, power failure is very common, even in Karachi. So is there anything that you, people are planning to better support?

**Speaker 2:**

Yes. yes. So, when we were trying to migrate on these so we had developed certain principles that we will not keep any of the assessment that requires, which our students cannot do actually. And we will not give the strict timelines. So, for example, if previously, when students used to be in hostel and when they used to be in face-to-face time, then we used to have very strict deadlines that, okay, if 15 is the deadline, so deadline means deadline, but when we were doing this migration and considering our students access and their remote situation. So we had also asked them that you can accept a few late submissions if it is within three days. And then what we also did was that few of the assessments had to be done through our face to face viva exam. So, those were the challenges and in that face to face there were certain examinations that in which we had to see what they are doing actually. So if they are checking the eyes, if they are checking the nose, so I have to actually see them how they are doing it. So those were our challenges and problem was that we couldn't say them that, okay, make a video and send me because the bandwidth was issue. So how could we support them? So, we had asked our faculty members to be creative as much as you can, in order to complete both of the things that their objectives must be complete and the assessment also be complete. So we had kept our faculty members open, and sometimes we thought that probably to me be impossible that due to the poor bandwidth, we may not be seeing everything, but our faculty members were so creative that they themselves extracted a solution that instead of asking them to submit their video, we are going to ask them to share their photographs powerpoint photographs, which is limited to these number of slides. So we are not exceeding the MBs requirement. And simultaneously we are also looking at their skills and, and assessing their skills as well. So this was our principle that we are not going to have a very strict timelines. And we will give flexibility to the students who are having difficulty accessing internet. Then we also were flexible that if you have any creative ideas, so please don't keep it to yourself and share and share in the curriculum committee. Then we had also discussed how we are going to Mark on the assessments, which are actually time bound assessments. And in that we have to assess their recall knowledge. So how we are going to do that. So for that as well, we allow the students to have password protected documents, and then you can submit the short question answers to us, and then we are going to accept that. So we have given our students and also that some of the students after doing everything, they were unable to give us tape recorded, video recorded in some of the assessments when they were not able to give us the video recorded, then we also became flexible to take their, for some of the courses we have also taken there telephonic viva. So, flexibility throughout was a key keeping in mind that we are not losing any course outcome or the learning outcome. And we are not losing any of our quality of our assessment. We are not compromising any of the rigor of the assessment, managing both the things were very, very difficult, but we have ensured that when we are flexible, we are also ensuring that we are not compromising the reliability and quality of the assessment as well, and teaching as well,

**Speaker 1:**

Right. So, good to hear that keeping quality in mind, and also a student's perspective, you people remain flexible so that this unusual times become easy for everyone, especially for students. So moving to the next question, I would like you to share your views regarding advantages of online teaching. So, over to you.

**Speaker 2:**

So, if internet is available, if it is completely an innovation of teaching/ a revolution of teaching, I would say. So, many of the aspects that I have noticed regarding the online is mostly face to face. and they were too much dependent on their faculty and they used to depend on the PowerPoint and slides and everything. But now when all the resources are with them, the repository is with them. So they know that they have to do it. So they are coming in classes prepared that I have seen they're coming in, classes prepared, they're coming for clinical prepared. They're coming for their skills, prepared, everything is prepared. And they know that if I miss anything, if I will miss that class, I will not be able to apply theory into practice. So it is making them more responsible. Number one is that I think if we follow all the procedures, then it enhances the rigor of your teaching, because then now what previously, what used to do, I used to deliver a face-to-face lecture and then students used to forget everything, but now what is happening that students are requesting, okay, ma'am, can you please record the session for us? So if we don't understand, at least we can go back and we can have this. So this is also benefiting our students that they can, they can rewind, and they can at the time of examination, they have too many queries as they used to have before.

**Speaker 1:**

They can revisit it.

**Speaker 2:**

Yes, exactly. So, what online education is also giving us an advantage of having lots of activities. So we are able to.. okay. So, once we are done with the class, okay, so let's have a post-test or let's have a review, so I can give them quizzes and they can keep playing with the quizzes and, and they can review. Okay. So how I did, so it is also helping them to become prepared for their summative assessment. So the opportunity to, for the formative assessment has also increased, which due to that time bound of the face to face class, we were not able to do as much as we could. but online is giving us an opportunity to, to play with the lot, many formative activities. so sometimes even I can say that, okay, so today I am not teaching it directly, but there will be five activities that you have to do. And that will allow me to complete your hours and the objectives will be achieved if you do these activities. So it is actually engaging the learners more as compared to the class face-to-face. So , when I was not able to engage all 70 of them, but sometimes it is, if I do this way, so everybody's, if everybody's doing the activity so they can get the opportunity for them, for, for the engagement becomes more. So if everybody's doing and everybody's reading and then doing their activities, so I will Mark their attendance on the basis of their participation and activities done. So, so this is also the advantage. And what else? I think this is, this is all for my side.

**Speaker 1:**

Okay. So what I gathered from you is like, online teaching is more engaging because, even as a faculty member, she can use various tools to have formative and summative assessments can be done online. And then you said if internet is not a problem, then, online teaching is possible any time. what, I would like to talk about like according to the advantages, does it have any relation to study routine or place of study? Would you like to talk about these areas?

**Speaker 2:**

Okay. yes. study routines, if you completely asked them to, to move to asynchronous, then I think learner become sort of careless in, meeting the timelines. And so...

**Speaker 1:**

So, you think it's a disadvantage.

**Speaker 2:**

Ya, it shouldn't be completely asynchronous for our students. It doesn't work actually the level of learners that we have. So, yes, they will be able to, but, but you have to really grade it, if you want to have the task done, then you have to grade it, then they will do. But if you say that, okay, this is my class today's class, and you have to complete all these activities, and this will be counted as your class participation. And the attendance will be counted based on that then five out of 70, will do it. So that's disadvantage. So therefore we encourage synchronous mode.

**Speaker 1:**

Okay.

**Speaker 2:**

Yes, please. Go ahead.

**Speaker 1:**

I was saying, we have started discussing about the disadvantages. I think it's the right time to ask you about what are the disadvantages of online teaching in pandemic situation and how can these challenges be mitigated? So if we can talk about these.

**Speaker 2:**

Okay. So the disadvantage number one is poor access students are disadvantaged a lot. And sometimes those who are having difficulty with the technology use, they also face challenges with the online teaching and learning. So for example, when I was teaching, and then it is also good that everyone has their own devices. So, our learners also have the habit of having a peer sitting besides them. And if they don't understand anything, then they slightly go and whisper to that colleague instead of asking the faculty member. So if the COVID has brought online modality, it's fine, we have all migrated, but our student mentality has not migrated. And if during that time, if that, learner who is a shy student who is struggling and who had the habit of whispering and the peers’ ear next to so that student is still struggling and, and how we encounter that learner when they come for a brief encounter during clinical. And then they say, okay, miss I missed this concept during online, and online is not good. I couldn't study online. I don't want to study online. So it has brought a lot of challenges when some, for those learners who were too much dependent on the face to face mode. So, it depends on the learner motivation actually. What I feel is, and there are some learners who don't want to change, but they need a constant reinforcement that there is no solution. If you will not cope with it, then you will lose everything. So I thought that this is one of the major disadvantage that I have encountered. You already talked about place of studying. So, yes, this is a challenge that our students encountered because although they were living in the urban areas, but, in our difficult living situation, so in one or two rooms, maybe seven or eight, family members are living. So, and when for, for one or two seconds of their mic is unmuted so entire, you will feel that something at the back lot of people are there. So it made me used to think that how that learner will be focusing and concentrating on the lectures. So that was a real concern.

**Speaker 1:**

So balancing the work family and responsibility was, um, like a disadvantage for a lot of people who think that home is not a conducive environment to study.

**Speaker 2:**

Exactly. And, then we had poor access issue, which I already talked about that, the load shedding, and poor connectivity. So they requested me that if you record your sessions, then even if we get disconnected, it will be easier for us to go back and review. but for example, if I'm doing an activity in the breakout room and the learner is actually going through a process of learning and what we are doing in the breakout room was an important objective which was important for the learner to understand that at that time, so that, that cannot be learned through recording that could be done through practicing. So that was also a problem that connectivity, if it is lost, then important objectives can also be compromised for that particular learner. Then, all the objectives couldn't be taught online that I realized, during my last few classes that previously I used to teach that face to face. And in that particular class, I used to Mark, take the scenario and Mark on my examination sheet. So, and then I use my learners to save to I wanted my learner to show me where they are marking. So I used to have a round in my class and I used to, Oh my God, I pick this up. Now you have marked it incorrectly. No, no, no, not here, go here. So it was easier for me to, to see where they are going wrong, but now when they are remote and most of them do not have the webcams. And if I allow all of them all 70 to open their webcams, my connectivity is gone. So, and the bandwidth is, becomes affected. So all the objectives cannot be done online. And it is very difficult to at times, ensure that all 70 are active and alert and they are engaged in the process of learning. It is at times very difficult to say that, out of 70 we only hear the voices of 20, but it is really, we don't have any idea of where those 50 are...

**Speaker 1:**

Is it like having large class size on online platforms and handling them is not easy or not possible?

**Speaker 2:**

Yes. And you can't have lot many activities then as well, because if 70 are there, I tried Kahoot that day and it was very difficult, 70.. how can I manage 70 on a Kahoot and out of 35, only, only 20 joined Kahoot. So 15, were again, saying, okay, ma'am my mobile is not working, or I have a connectivity issue. If I will do this, then I would not be able to, Oh ma'am, I am joining the class from mobile, then how can I go to Kahoot now? So, so device availability was also a problem. So I think they ensuring that all 70 are learning was difficult with the online classes and engaging the large class in too many online activities was a challenge from where I come from it, from where my context is, because all the students are not having all the devices with them. And, most of the time they are joining classes from mobiles. And if I see, okay, share the screen. So they say, Oh, well, sorry, I cannot share it because, and then show me, where are you? So, so from learner point of view, you get sometimes, it is difficult to engage them fully in the process of learning.

**Speaker 1:**

Right? So, thank you for sharing deep insight about advantages and disadvantages of online teaching. I think it's time to move to the next question where as in the beginning you discussed about the fears of faculty members. So now I would like to know that what are the competencies and skills that are required by faculty members to teach online during the crisis.

**Speaker 2:**

Okay. So number one is, yes, command over a VLE, okay. Having, command over other tools as well. Other, for example, we can have Mentimeter, we can use kahoot. We can be, we now have other mechanisms such as quizzes so that we can engage the learners if it is difficult to engage them in the synchronous mode, then at least they are engaged through asynchronous mode because learner engagement is the key all the time, whether it is face to face or online. So if you are in the class of 70, if you are not able to hear the voices of 50, then it is our responsibility to ensure that they are learning. So we should be having certain strategies that help us, know that they are engaged.

**Speaker 2:**

So, yes, what the tools that I have used so far is a Mentimeter. I have used Kahoot. I have used, the quizzes and, what else I have used... I have used several tools from my own, VLE, which is H five P we also use the feedback form for taking certain evaluations at that, how they are going through, besides that, we also should know, because certain skills also have to be transferred now to the online modality. So we should know how to plan it effectively time wise, so that we don't face to face it was okay, but for online, we should be extra cautious with the planning part because all 70 will not be face to face with you. So if something went wrong, it will be very difficult because students are not seeing you what you are encountering at that time and what is going on. So, your planning should be thorough. Your team, should be on the same page, while, you are in the mode of the online teaching, then definitely using the Microsoft teams or zoom is important. And, using that tool is that these two programs are also one of the competency, which is required by all the faculty members.

**Speaker 1:**

So, need to know how to operate various features of zoom and various tools or educational tools. We can say, like you mentioned a lot of them, and you have even answered my next question, that, how do you have effectively engaged students? Like you said, you used Kahoot and then quizzes and Mentimeter. So I would, again ask you that, how can the faculty effectively engage if there is anything left you can add. So how can faculty effectively engage students in online learning environment? So one of them that test tools using educational tools, any other,

**Speaker 2:**

The slides that we develop, sometimes become wordy. So, from now, what I do that I have turned the shape of my slides, that they are colorful slides, and instead of writing content on it, I used to give them picture or reflective question to think about it and answers. So my every slide has some thought provoking question or an activity. So, it depends how you develop the slides and how you... so let them think, and then there, they will respond. Sometimes I also ask them that, okay, so we are playing now fastest round. And then, I will take the, I will ask the names, but then I will ask the question with the names. So everyone has to be very, very alert. And so everyone becomes alert, and if somebody is not there, then they respond, Oh, ma'am, I'm there. Actually, my mic was muted or not working. So they, the students know that, I can be called anytime during the online class, even, if I'm not seeing them, they should know that faculty will ask a question and I can be asked to answer. So this was one of the strategy which, helped me to make my learners engaged.

**Speaker 1:**

Right.

**Speaker 2:**

And then annotations tool that I use, so that also help us and white board tool also help us to know that how many participants are writing actually, and they are engaged.

**Speaker 1:**

Hmm. So using of educational tools and then using of various features on zoom, like annotation and whiteboard, and asking random questions from the students, make them alert and keep them engaged during the online learning. Right. So let's move to the next question. And it's regarding the students residing in remote areas. So what are your views regarding the use of online teaching and learning for the students residing in the remote areas or different parts of the country? So you have discussed about internet connectivity issue. Any other thing that you would like to talk about?

**Speaker 2:**

This is actually the sore point of when students do not have internet connectivity, how will you connect with them? And some of them are even not having the smart phones. The video calls cannot be done with them, or so, and some of them are not having the access to WhatsApp even. So that actually make learning difficult that this is a sore point that you have right now in ensuring an equally engaging them becomes difficult. So, for that, a yes, it was for us, it was completely a remote experience in which what we did was that even we couldn't send them the material through the .. online.. or VLE was even not accessible to them. So what we did was that we provided them all the content, all the PowerPoints in the USBs and USB was provided to them. And we also shared with them the schedules of everyday starting, but that the students anxious that made the students what we would say learning fatigue. It was like learning fatigue, learning by their own or reading by their own. And if they don't understand anything, they were just, they were just struggling with every concept that how, if they're not understanding how we, to whom to ask and how should we attempt those questions, even, even the assessments exams, we sent them through USBs and they were password protected. And, when they received that they had to study simultaneously. They had to do the assessment and SAQ questions as well. So, so that, that went like this, but whatever, I heard from my students, they said that it was very difficult. We were having, if we don't understand anything, then, then how many things should we ask? Some of them were not having WhatsApp. They were not having access to WhatsApp group as well. So they were not very attentive on the discussion going on through the WhatsApp. So they were sort of, they suffered a lot and they are suffering a lot. So this time what strategy our teaching and learning office took was that HEC announced that only 30% of the students can reside in the hostel and remaining has to leave. So, it was decided. It was a very difficult decision that was taken by our DEAN’s office that year 1 and year 2 will be sent back and only year 3 and year 4 can stay. So only year 1 and year 2 before they were sent back they knew that the material will be inaccessible after they leave. So what facility we give to them that you read everything, you complete everything. You complete all the assessments and then you go. So this was the support, which was given by the teaching learning office so that they, they are not lost and they do not face that learning fatigue, again, reading by themselves and sitting by themselves. And there is no teacher, there is no peer, there is no interaction. So that is a sore point that when the internet connectivity is not there, yes, learning is going to suffer. And the hub and spoke model was created. However, our students shared with us that it was so far from our residents and accessing those hubs was actually, they were, yeah, they are not part of those hubs. And those hubs were the property of other people, other students. So they were not getting... they were like aliens for those places. And they were, they were accessing it as a favor. It was not their right that they can access it, but it was a favor from the community given to them not as a right. So, so they had difficulties accessing it if, and they had to follow their time in the, when they were in the university, they were on their own. And they, they, they used to study at whatever the time they wanted to, but now they had to follow their time strictly because then the school will be closed. They will be sent back, even if their work is not done. So the quality of assignment that I received from those who were, those remote areas, it reflected that what type of difficulty they went through. And, fortunately the semester was pass and fail. If they were graded, they must have been at a very bad disadvantage.

**Speaker 1:**

Hmm. Thank you for sharing. Very important point. And I think you said it right, that this is something that you people have faced during this crisis, especially the students who suffered and who are suffering right now due to what we can say, the lack of facilities. Well, the next thing that I need to ask you, it's regarding the complete shift or the migration to online modality. So what are your views regarding it? Are we prepared or there are still we have some fears or anxiety...over to you?

**Speaker 2:**

I think Most of us are adjusted to it. but, some of those actually in our school, there is a shift actually. So in one of the program was 70, 80% online before COVID. So they were like on their own, they, they were, the students were given a good educational technology courses. Students were quite happy, and they were quite used to off using the online modality. So that was our post RN BScN program. Master students Yes, most of them are accustomed with, but this batch was also from, most of them were from Gilgit. So due to lack of access to internet, their classes were also hindered. So, if they are also saying back then again, master's program also face difficulty of continuing the classes. So this is one. Now we have the four year BScN program, which is a majority of the students are in this program and almost 150 per year. So you can say, we can say almost 600 students with, we have in the four year BSN program. Okay. So now the year one and year two are the one who are the most disadvantaged because they, they, they have not got all the opportunity to, to feel that migration. They are, they have mostly seen the remote teaching and learning. So they came here for, for a month or so, and they were trying to adjust, but they couldn't feel the full scope/full flavor of the online classes and online teaching. So, at that time priority was that quickly let them do their, those objectives, which are required to be done face to face. So they, they quickly completed that, those chapters. And then again, they were sent back due to HECs notification. So year one and year two have not experienced that the faculty members have also not experienced the full scope of online teaching and learning, those who are in year one and two now coming to year three and year four, they are here. And faculty members have experienced, the students have also experienced the full scope of online teaching and learning. And we are implementing every new things that we can do. and I already told you how we are improving day by day. And so, at last, what I internalized through this experience was that when we started, we considered ourselves that we are not prepared and how this will happen. There were lot many apprehensions. When we started and gradually, there was a hope of light that the online assessment, training and then various sessions from QTL net, and then the support from teaching learning office was that our IT experts were always there if any issue encountered, they were there to resolve. And there, it was overwhelming for them as well, but they were not saying no to anybody. That was a big, big blessing. So that helped us to actually dive in the pool of online teaching. We started delivering our classes. We started delivering, we didn't know how it will go, but when we started with then we came to the, okay, the problem is that students are not engaging. Students are not answering. We had strong apprehensions that some of them have really opened, they have really signed in and they are not physically there and they are not listening anything. But one thing that we had ensured was that our summative assessment was made in such a way that we prepared our students earlier in the beginning that look, if you are not going to learn, or if you are not going to take classes, then you are going to lose your many of the things in your summative assessments, you are not going to. So they were adult learners, they understood and then we started and then we started giving them classes. Many a times students also shared that you don't use other modality other than the PowerPoints. So you should also use, so this was one of the feedback, which was shared by students. So we tried, okay. we also should have other modality so that they feel engaged. So then we gone through that experience now where the turning point was that after completing all the theory there was a clinical rotation, and there was a skill rotation where we were going to see our students for the first time. So when I went to them, they said, okay, who are you? And then, when I saw them, I said, who I knew, so where are my students? So, so we were completely new to each other. We had never seen each other, but what was making us recognize was our voices. They were knowing us. And I was knowing them with their voices, not with their faces. That was completely shift. That was a complete shift. And that was sometimes also disheartening that, and passing by, but my students are not greeting. So that was one worry. One of my faculty also told me that what happened, you know yesterday a student knocked my door and when I opened it, I was not wearing the mask because I was alone in my office. And when I opened the office, she said, where is Ms. Sadia? So, can I speak to Ms. Sadia? So she said, I am Ms. Sadia. Oh ma'am, sorry, I couldn't recognize you. You are there. So that was a complete shift for us. It was also not what I would say. It was something which was, we were not expecting that our students were not going to recognize us, but the only connection that we could have was our voices. Then when we took the class face to face the first class, because it was a skill that couldn't be done online after a day or two, when we spent with them, they realized that ma'am, trust us, nothing can take the, nothing can replace the face to face teaching nothing.

**Speaker 1:**

So what I am gathering here is first thing that there are two aspects of it. Like if you talk about the demand of this particular nursing course, it has some theory courses and some clinical courses, right? So for theory, it can be shifted completely to online modality, but when it comes to clinical aspect, there are some restrictions because students need hands-on practice. And, the other thing that you shared is the interaction, which is missing. And, the one example you shared that students are unable to recognize, or it seems like that, the bond has not been created due to online mode, which is possible in traditional learning is what you are trying to say is, am I making sense to it?

**Speaker 2:**

You are right. You're completely right. Because when we write exact connotation, but it doesn't communicate to them that I'm being polite or I'm being humble. And sometimes they take me that, okay, I'm, I'm bit rude with them, but when they, when they came face to face. They gave me a feedback. Ma'am you appeared, you sound so rude on the zoom, but we never knew you are so much humble. And you are so much concerned about students learning. You take students' concern so seriously. So yes, it is a barrier bonding doesn't develop when we are online, especially, especially, and when the, all the learners are not at my level, they are actually kids actually 20, 22 years old adults set group, I deal with, so yes for going to their mentality, the bonding has to be there, which is actually not, not happening in the online modality. Yeah.

**Speaker 1:**

Right. So, so thank you for your response. We are left with few more questions. So, the next one is what have your experiences of supporting the planning of students' assessments and grading in complete online learning environment during pandemic? So you were as a part of the team, like you were supporting the student assessment and grading, right. So what were your experiences?

**Speaker 2:**

So this is our norm that for every assessment there has to be a rubric. So we ensure that, whatever the assessments are proposed, they must have their rubrics. And when the students are evaluated, they are not evaluated on a criteria, which is actually assessing their use of the technology, but it is assessing their conceptual clarity. So we also tried to based in these types of principles, when we were approving the assessments, what we did was that those students who have a complete, full access of online modality, we ensured that our bank questions, which are available in department of educational development, they can be used so that the reliability and credibility of the assessments can be maintained. And we did this the same with the year three and year four, BScN and postRN BScN, so that those who are having full access, complete access with good bandwidth can have at least the reliable assessment. The problem we faced was with year one and year two, to whom we were compelled to provide short question and answers, this increased faculty members' workload for too much checking. But unfortunately those who were in the mood destinations, we couldn't have, we couldn't give them the closed book exam. So the open book exam was one choice that could be given. And we didn't had any other option. So for them, year one and year two we had limited options for giving different sorts of assessments, but for year three year four and postRN BScN we applied all sorts of technology can be any pause, the recording, or shall we, shall we wait for a while? There is somebody at the door

**Speaker 1:**

Okay, I can pause it so you can continue. So you shared about the year one and year two, and then you were talking about more. Yeah.

**Speaker 1:**

Can you repeat the question again?

**Speaker 1:**

I was asking you how you worked for your experiences when you support the planning of students, assessment and grading in complete, online learning environment during pandemic.

**Speaker 2:**

I covered that, that we ensured that the grading is done on the basis of fair rubrics are reliable. They are, they are approved from the curriculum committee prior. We also ensure that all the assessments are approved prior to administration from the curriculum committee. And, no unplanned assessments are administered to the students. We ensured that the questions are available from the bank. They can be used because which are already tested, which are already being tested and they are reliable questions. So this way we ensured that quality assessments are given. One challenge we encountered was that in medical college, the assessment, the close book assessments were conducted with the help of proctoring. Okay. But, so Proctoring is a technology which allows, online invigilation. And if a student is cheating or something, so, the faculty members can actually detect the plagiarism. So, for that we had a limitation because our students didn't have all the devices required or for that particular sort of invigilation. So what we did was that we developed our own guideline for the online examination. We didn't give any closed book examination, but we ensured that those questions are given, which cannot be easily Googled. And, we ensured that students we gave all the examinations which were open book and which doesn't require any invigilation. So, but they were time bound. So for example, if at nine the exam starts, there will be 60 questions. So if nine at nine, if they start, so at 10 30, they should end, and there are 60 questions. They will be long scenario-based questions. And if you go and cheat, so definitely you are going to lose four or five questions, which are an attempted, so students were aware. So what we did was that we administered wisely all the assessments that ensure that our students are able to do it. And if they cheat, they will be losing… they will be conducting one question through cheating, but the time will be so less that they will be missing four or five. So it was designed in such a way. So, due to that, when the results came, we compared those results with the print of the previous, closed book face-to-face examination. And fortunately in at least my course, I'm confident to say that there was no significant difference in this open book and close book examination that we conducted. So, so we were able to identify bottom scorers and in all the other courses of my year that I teach in year three. So others were also saying that we also encountered that those who, those students who were found to be slow learners in other courses, they were also found to be having a bottom scores in other different courses. So, our guidelines were developed in such a way that the slow learners or the bottom scorers were identified and high achievers were identified as well.

**Speaker 1:**

Right. So as you were discussing about the challenges that you faced while using that particular, software or application, I would like you to talk about some more challenges that you faced in implementing online teaching. So not only assessment, if you talk about online teaching or while supporting the students and faculty members, were there any challenges that you faced?

**Speaker 2:**

Yes. So students competency in using the different tools of the computer. So one was that their availability of the devices, this was also a challenge and gradually our faculty members become accustomed with all the technology and the skills required gradually. They are learning, and I would send, at least none of them are there who are completely dependent. All of them has tried their best to meet, uh, the requirements for online teaching and online teaching also helped us to make all everything electronic, actually. So now everything is electronic, everything is in writing. So that also, uh, was a benefit, uh, for online teaching and learning. Uh, so, uh, other challenges, I think the major that I felt, uh, I,, faced was from student point of view. And one thing I would like to add here was that, uh, yes. Oh, I also told you about. It was difficult to know that how many of them are actually attentive and how many of them have yeah. That was also a problem.

**Speaker 1:**

A large class size was a challenge.

**Speaker 2:**

Yes. Yes. Large class size was a challenge. Marking attendance was a challenge at times. Microsoft teams was a challenge. Yeah. Yes, yes. So Microsoft teams itself was a challenge because everyone cannot have, uh, every week for every class, we cannot have zoom. So for, for, uh, in our, in our, uh, school, what was that, that, uh, all the undergraduate classes used to be conducted on Microsoft teams. So it is a free software, but, it has several issues that you cannot use the full fully scope of all the tools you cannot, because it is slow. It, it actually make your system slow and it hangs many of the times, or you cannot see the full participant list, uh, because, because your students are coming as a guest that you cannot see full participant list. So marking the attendance was, was a challenge on my Microsoft teams.

**Speaker 1:**

Okay

**Speaker 2:**

yes, Microsoft teams was difficult for managing the large classes for some, some of the classes where I thought that Oh my God, this cannot be done on Microsoft teams. Then we used to ask for a zoom link, which our program coordinator used to provide us, but not on the short notice.

**Speaker 1:**

So what are those things that cannot be done on Microsoft Teams.

**Speaker 2:**

for example, just, I shared with you that we couldn't see the full participant list, so some of the, because our students are not AKU employees. So all the guests, you cannot see all the guests, then everyone has the host right. It was the problem. Yeah. So everyone, when everybody has a host, right. So if I don't want a student to admit, so other can admit, then in the chat box, sometimes all of the students do not have access to the chat box. Okay. This was a challenge then, some of the students also said that we do not have access to raise hand option because we are guests. So this was a challenge, right. Then, one more thing was, let me think about it, which was a very important issue that I encountered with teams. Pause..... Yes. Breakout, breakout group. During teaching, you cannot make a breakout group. If you want to make a breakout group, then you have to inform the coordinator prior to the session, at least two to three... These are my groups. So now divide them and make separate teams for them. And then in zoom, it is easier for you to see all the breakout groups, and then you can switch going to students to facilitate them what they are doing. Right. But for teams, it is not possible.

**Speaker 1:**

Okay. Yeah. Right. Thank you for sharing a lot of challenges. Uh, I would like you to, uh, tell me about recommendations. So what are your recommendations to ensure sustainable remote teaching and learning in the future? So anything regarding resources or anything, which was, uh, lacking. So any recommendations

**Speaker 2:**

Yeah. Something has to be done in a longer run. something has to be done for those who are disadvantaged.

**Speaker 1:**

Yeah.

**Speaker 2:**

Yes. Because they are disadvantaged. They just need support. They are having every sort of, I mean, in terms of poverty.. So yes, they are not having devices that yes, they are not getting every sort of support they need. So something has to be done for them because ultimately they will be the sustainable members of the workforce. Those who will be powerful enough, they will go away leaving the country and the profession behind anywhere. But, those who choose nursing, they are actually willing to change the fate of their families. And they are mostly from disadvantaged background. And, the university should identify them and, and do something for them that at least to sustain connectivity for them to sustain access for them, so that their learning is not hindered. One thing is that and continuous faculty development for online teaching and learning, and, then also forum, where faculty members can share their challenges, this has to be there so that, proper recommendations can be brought into, the refresher for online modality is also required. Yes. , and one thing that yes, I understand that for year one and year two we had to test all the modalities that we could, we could ensure their learning is not hindered and they are progressing well, but I still feel that, , they're teaching learning, and when they will come to year three because their clinical has not been done yet for year one and year two, year three, and year four. We have ensured that when they are entering the workforce, so they are having ample clinical exposure before they are moving to year four. And before they are year four, before they are going to the workforce, but for year one and year two, and just having an apprehension that when the year two will be coming to your three, they will be completely blank. So the school has to think that, how we are going to manage those lost clinical hours because nursing education cannot be completely online. So that is also important. And, when we are saying that yes the opportunity for virtual reality simulation need to be explored more so that our students are more engaged and, not only the virtual reality simulation, but how faculty members can transform their previously developed classes into online modality not compromising the principle of engagement.. Learner engagement. Yes.

**Speaker 1:**

Okay. So, uh, thank you for a lot of recommendations that you have shared, uh, last two questions. The second last is that, how do you see university support or role in executing, remote teaching and learning program?

**Speaker 2:**

I see the role of QTL net has been very important in doing that in sort of capacity building of faculty members. And, mostly it is nomination based or wish based, that sometimes if I want to go, if I have time to attend that tech lounge, if I had time to attend boot camp, then only I will go. But certain workshops that QTL net can do, compulsory entity wise, for example, one day QTL net is coming to SONAM. And this session is mandatory for all. Everyone has to do it. And one day it is going to DED one day It is going to medical college. So this sort of Mandatory capacity building has to bepart and parcel. one thing that university can do and university has also ensured that those faculty members who were not having access to proper devices, they are supporting those, but there is no announcement, or there is no email open that those who are facing difficulty, so they can talk to this person, if somebody do not have the device, or if they are working from home and they do not have the proper device to work from home, then I don't think so that there is any particular announcement or email from the leadership that if you are facing difficulty, you can have, you can contact this person and you can get your devices or so, so I think yes, this is the issue. So protection is important. And one more thing is that, we have been told that 50% of the faculty can work from home. So remaining 50 can come, but sometimes if I have to take a large class, I cannot risk of connecting from home, because internet connectivity is uncertain. And if I get disconnected, my 70 students will suffer. So, if I decide to come to office and if at the same time, my roommate is also having the class, then it was used to be a problem because, when I used to speak, she also used to speak and everything used to get chaos. So yes, space is a problem. This is also a problem which a university can be revising the sort of layouts existing layouts. We need to revise that. For example, if this class is going on. So previously we used to give venues for classes, but now there is no infrastructure. So in the online modality, if this class is going on, so all the venues are vacant And how should I go for the booking? There is no support from the program office that, okay, this class and this venue is booked for you, so the students will be online, but you have to go in the class and you have to conduct the class over there. So, this was not happened. I should sit in my office and I start taking the class no matter whatever the background is, whatever my colleague is speaking. So that infrastructure needs to be developed.

**Speaker 1:**

Right. Uh, so this, I have noted down in the recommendations as well. Um, so moving to the last question that, uh, how SONAM, uh, can be a trendsetter or a role model in introducing remote learning program to meet country's nurses, demand and healthcare system.

**Speaker 2:**

I think capacity building, if we are doing so much, then, we should publish this and write these papers and not only publication but also we should impart this knowledge to the other universities and we should develop their capacity and SONAM has several grants which can be used to do so, we should teach, we should train other institutions across Pakistan, that how we have gone through the online migration and how we have sustained the teaching learning. So the similar workshops can be conducted by our faculty members. So we can show the institutions across Pakistan that how we have reached out to them, to our audiences and to our learners. So one thing can be that we should follow the best practices all the time before applying any, any new concept in our practices. So, that curriculum committee ensures, and from curriculum committee our processes are quite transparent that, once the faculty writes the proposal, they come to curriculum committee and then from curriculum committee, it goes to RWG. And from RWG, it went to HEC. So our processes were quite, Stringent and streamlined prior to whatever we applied and implemented. So that can be sustained in future as well.

**Speaker 1:**

Right. So following the best practices in showing what you are doing through the publications, or sharing it with other departments, I think this can, uh, help SONAM to be a trendsetter and a role model,

**Speaker 2:**

Not only the departments, but other, uh, nursing universities and other competitors.

**Speaker 1:**

Right. Um, any other thing that you would like to share?

**Speaker 2:**

I think I have shared everything.

**Speaker 1:**

So, yes, your ideas and answers are valuable to us. And thank you for taking out time. Uh, last thing that I need to tell you is that still, if you feel like, uh, sharing anything, or if you missed out, or if you think that, uh, it's right now, it's not a comfortable place to share in if you want to write. So there is an option of writing a reflective log, and you can email that on the same address that from where you received the zoom link. So that, that is another sharing.

**Speaker 2:**

If I feel that something very important I have missed out.

**Speaker 1:**

Okay. Right. so I think that's it. And, thanks once again for taking out time. Is there anything that you want to clarify or you want to ask otherwise we can end the meeting?

**Speaker 2:**

Okay. So for postRN BScN, fortunately whatever we designed was implemented, in the same way, because our students had most of them, almost all of them had access to the internet and they were available to take classes online and, but also available to perform the time bound, synchronous examination as well. So for the challenges that I talked about earlier regarding limited access Post RN program was exempted from all these challenges. but yes, for postRN program, what we encountered was what I, as a faculty encountered was that, since the students are, coming and most of them are part-timers or they are doing their studies with their job. So their engagement used to be there much more when they were face to face. But when the modality has changed to online, we faced that, that they were slow slight, their engagement, engaging them was a problem initially when we were doing the online migration gradually, but, with them, it is a problem always that yes, because they are balancing two things. So their performance is not the same as we see other programs or other students doing who are the full-timers. So it is a challenge for postRN students always. but from students' point of view, it was at the student's level, students found it doing it whenever it is feasible for them, they didn't required to travel to attend their classes. So, so yes, it went into their favor. but, I being a faculty felt that maybe face to face learning would have benefited them more.

**Speaker 1:**

Right. I think, uh, this is, uh, specifically to PRN, group, so that will also help in enriching the data. So, uh, thank you, Ms. for your time and, uh, uh, talking about all what you felt and what you experienced. So that's it from my side. Is there anything that you would like to share further? You can write a reflective log.

**Speaker 2:**

Sure

**Speaker 1:**

Thank you once again. Take care. Allah Hafiz

**Speaker 2:**

Take care Allah Hafiz

**IIKI 3; Date: January 22, 2021**

Speaker 1:

Thank you so much, Sir . So, is there anything that you need to ask me otherwise I will begin with my questions?

Speaker 2:

Yeah. Just go ahead. No problem.

Speaker 1:

Okay. Okay. So the first thing that I need to ask you is what are your views about online teaching learning?

Speaker 2:

I would say first that it's the way of the future, is the way of education. The online component, especially in teaching and learning. It's something that has been ignored for quite some time. At the time we had the pandemic and it was .. People had to just go online. Some went into online to be able to survive and remain relevant others were like this is the way that we really wish to teach. And it's like they want to know more, especially in terms of how teaching and learning should be done online. So what I want to say is that online teaching and online learning is the way of education and it is something that we really need to embrace. Thank you.

Speaker 1:

Right. So, what I gathered is that this Covid has become a window for opportunity for us in order to experience this online teaching and learning, which was neglected before. Right. So let's move to the next question. And it's regarding your experiences of supporting the migration of existing curriculum to remote teaching and learning during the pandemic and how can faculty and students be better supported in this transition.

Speaker 1:

So shall I repeat the question?

Speaker 2:

Yes, please do repeat the question.

Speaker 1:

So what are your experiences of supporting the migration of existing curriculum to remote teaching and learning during the pandemic?

Speaker 2:

Okay. So maybe just, I start with that and then when it, whatever, whatever I miss out, maybe you can just be able to remind me in terms of the experience, especially for the migration of the curriculum. I think it was met with quite a number of challenges depending on where faculty were in terms of use of technology. I know that it was quite difficult because people wanted to do what they've been doing in face to face to online, somewhat running a three hour session, maybe on face to face and they wanted to do the same. And they found that by the end of it all, it's like, you don't have all the students, the students have faced with a number of challenges and it is like this class is not as smooth as you really expected. That's why in supporting faculty, what we did is that we asked faculty to be able to look at what they're going to teach plan very careful so that there are some things that they should be able to cover asynchronously and others can be covered synchronously. So we really advocated for those two months in terms of online learning, that is the by chronous kind of blending, that is a synchronous and asynchronous, where now we worked with faculty without those ones, we would even meet every week to be able to see to it that whatever they want to use asynchronously that is on the VLE Moodle was done, especially in terms of how they were able to package or their content on the VLE class, the activities that were associated with it. The VLE that is used in Aku that is the Moodle. So we had someone who had already started teaching, using the VLE during face to face sessions before COVID. And those ones had quite some easy time, a in terms of having their materials uploaded and making a few adjustments here and there, but the ones who had not, then they had to struggle to be able to fast learn how to use the virtual learning environment, the VLE before now, you are now able to have them packaged or have the information, the content package for teaching and learning. So it was a bit of a getting people from where they are, those who already had an experience with VLE and those ones who had not. And we worked with the, both the groups. We had quite a number of sessions across the schools including across East Africa, across Pakistan. So that was quite a bit heavy for us, but we work tirelessly to ensure that we are supporting them across, because we had our support required across SONAM across IDE across GSMC. So that was quite a bit heavy for us, but I think we were able to manage since we've been working with faculty for quite some time. So that is what I can be able to say in terms of the curriculum. It was met with quite a number of challenges as I could be able to point out challenges in terms of connectivity, even for faculty, because you could be handling them with a session in terms of one-on-one consultation. and it is like by the end of it, or they are, some of them are disconnected during the sessions, but what we learned is that it's the whole idea of being patient.

Speaker 2:

And you have to be patient, especially with the technology, be patient with them so that you are able to bring them to speed. By the end of the day, I think it was a, it was really, really appreciated. So some of the things that we took them through act points, we would have like all group discussion or whole group sessions where it's like an entity. We are able to meet all of them and be able to reflect on their experiences in terms of teaching what challenges and how we can even support them better. That is a whole group. At one point, we even had small group sessions. We have maybe faculty-wise sharing sessions or lessons units could now be able to come on board. And we are able to check them through certain areas that they find difficult in, and also be able to introduce to them some of the things, the new pedagogies that they can be able to use even during this particular pandemic. So we had this small group sessions. We also now apart from this small group sessions, had what we call the one-on-one consultation clinics. And those of us who are able to meet with a particular faculty, one on one, and you're able to address some of the issues that they are facing, especially in terms of the technical needs, the pedagogical aspects, some would even come and tell me next week, I'm teaching this particular lesson. This is how I'm going to present my session and the comments that you have be able to give me an input would help me. So those are the things that we would really take them through. And it was quite a very kind of interactive session. So depending on the entity and whatever people were interested in, at one point we name and have had what's, now we could call reflective sessions with ease some of the entities. So that to be able to just also be able to look at, if they are able to teach the whole semester, what are some of the things or lessons that we can be able to learn from the experiences, and then how do we support them better in the next semester? So that is a, those are some of the things that we can be able to share, especially in terms of moving that curricula to online. So it wasn't, it wasn't like it's too, just to be like uniform one, fit for one size fit for all. But it was that you really needed to understand the needs of the faculty so that they are able to support the students better. Yes. Thank you.

Speaker 1:

All right. So, what I gathered is you, people planned group sessions, you people planned reflective sessions, one-on-one consultation and need based sessions that were effective for the teachers. Right? So, I just want to ask you, we don't know how long this pandemic will take and might be transition will be for one more year or whatsoever, six more months. So is there anything in pipeline to better support this transition or you will be carry on with the same practices that you were doing.

Speaker 2:

Okay. The same practices will still continue. We always in our entity QTL advocate for one-on-one sessions. So if faculty have some needs, we expect them to write an email and then we set up a session for them. We also are open to all group sessions. If one entity had faced that we need to reflect with their teams, we are also open to that. We also advocate for small groups. So those sessions will still be able to push them in terms of support, whether the pandemic is there or not. Because a teacher, you are not really the owner of it all, you are not an expert in everything. And that's why we would also want to take those programs for one, especially in supporting the curriculum or offering curriculum support to our faculty, remember they are not like they're trained instructors or teachers. So the support is supposed to be like an ongoing kind of a thing. And remember, curriculum is now not like a fixed kind of an entity. There are new things that I imagined out of it. So that's why those programs are supposed to really continue. But alongside that, we now expect them because as a team BDL and QTL, we are putting programs in place that should be able to support faculty throughout the year. So we are bounding workshops. Those ones will also be able to out of those particular workshops, there are new things that will emerge, faculty will need some more support in the same, so that support will still continue even after the pandemic.

Speaker 1:

All right. So, anything regarding students point of view, like, to help students during this time, or any workshop that planned for students, anything like that, or this is all for the faculty members?

Speaker 2:

No, what we do is that when we are planning for these sessions, we always have the student in mind because now we are not talking, not talking of justice, student centered, we are talking of learning centered. So when it is learning centered, then the learner is like at the center of everything. So we are doing all these, have the learner in mind. And that's why we now have we're catching. We have now as standardized kind of a program when it comes to student’s orientation and not the general orientation for the entity, but student’s orientation when it comes to the use of the virtual learning environment. So we take them through a 90 minute session to be able to understand their online requirements. So, so far, like from the time they year began, we've dealt with some two entities that is the ISMC students. We had an orientation with them. We've also had the orientation with the medical college students in Pakistan. Yeah. So you can see, we have a program, a standardized program that has been approved by even the registrars group, the registrars group, in terms of students orientation, it was approved. And that is now what we are using each takes 90 minutes, as I've already said. And it goes through some of those things as it is, as opposed to really expect, we will do some evaluations to be able to see that we understand them because they bring on boards like their fears. They share with us the fears also in the introductory part, they also share with us some of the hopes and how they expect even to learn at AKU. So that is what we have. We have a very, like a vibrant, now I can call it to use the word vibrant kind of orientation program that is now been able to be shared by all the entity heads. And it is now being used across.

Speaker 1:

So this is in planning. Okay. Thank you for sharing this point. Let's move to the next question. So in your view, what are the advantages of online teaching?

Speaker 2:

The advantages of online teaching. There's a lot in literature about online teaching, but what I can say is that when it comes to online teaching, it's like we're now in the 21st century, just looking for just-in-time learning. You had of that just in time teaching or just in time learning. Yes. So if we really want to push just in time learning, then online learning is the way to go right now, I'm talking to you from the comfort of my chair in the house. And we are able to like go through this particular session, which to me, I call it a learning session because I'm learning from you. And you're learning from me. And we are saying that online teaching or learning is able to support the 24 hour kind of support we require for our students and faculty. And also seven days in a week so we have no excuse. We don't have an excuse of a missed lesson. We don't have an excuse that I was traveling. I could not teach. We don't have an excuse that I was sick. I could not teach because you will still teach from your sick bed. In terms of your lesson, you can still be able to facilitate that as long as you have some little strength to be able to do that. So when it comes to online learning, it comes to teaching and learning. It comes with all the advantages. Although it's been looked at it traditional from the traditional perspective, very negatively, and it has been marked in many, many aspects, based on the challenges that people have on some of the investment that has to be done for online to take place. Like for example, investing in the infrastructure in terms of the laptops, the connectivity students might not be very comfortable loading their laptops with bandwidth, unless maybe they're provided with data bundles, but institutions providing data bundles that is not sustainable as what some of our entities have done. It will be a short-lived or short-term, but it is not a long time measure. So the question would be how would we as a nation or countries be able to invest a lot in terms of internet so that we able to also match the developing countries, in terms of the connectivity, so that when it comes to coordinate learning or online learning, it is just like the way we've treated or looked at the traditional, or the face to face kind of teaching and learning. So I would say that, even after the pandemic or a post COVID, what we will have is that we will have what we call it, blended kind of learning. And that would be the right way to go, especially when it comes to addressing quality. I look at the way things, people have been scrutinized in their classes in terms of what are you putting up? What are you going? What is going on? Like unlike what has happened in the face to face sessions, if somebody walks to the class, they can even go to class, just give stories to students. And it is like the three hour session has gone. But when it comes to online, Sir is asking you, what have you put up? Where is your course outline? Is your course outline, being able to meet, or is it in line with the learning outcomes that you've been able to come up with? How are you going to teach? What strategies are you going to use? And like what used to be there in terms of face to face. So that's why I'm saying I'm not being biased, but having had an experience of both face to face teaching, I have taught face to face for over like 15 years. having also taught online for another like 10 years, I think I can be able to confidently say if we are really to address the issue of quality teaching and learning, if we are not being purely online, we have to really do blended learning where we are blending between face-to-face and the online sessions. So that is what I can be able to say. I have a lot to be able to share on that, but maybe because of time, I stop down that question.

Speaker 1:

No, you also answered a lot of other questions as well. The advantages that I have got from you is that it's not bound to place. It's not bound to time. And a person who wants to learn can learn from anywhere and wants to teach he, or she can do it from anywhere. So, right. Sir , lets move to disadvantages other than the internet connectivity issue and power failure and all that you haven't mentioned, what are the disadvantages of online teaching and pandemic situation and how can these challenges be mitigated?

Speaker 2:

And so in terms of the disadvantages, now that we are coming from third world countries, I'm referring to Pakistan, I'm referring to Kenya. I am referring to Uganda Tanzania now that we are getting, or we are coming from third-world countries, can be sure that, we can talk of as many disadvantages or challenges as possible, when it comes to online. But as we said, when it comes to online we are tracking of any time, any place anywhere, and, you know, when you go anywhere learning and anytime learning, it has to be made with quite a number of challenges, because sometimes there are those distractions you are talking about people missing that kind of physical presence apart from the social presence that people would really want to have. Like now, I really wanted to have been seeing you and seeing how your lips are moving, how your eyes are connecting with mine so that I am able to see, are you understanding what I'm saying or not? So that's kind of a component. And the advantage we have in Aku is that we have like small classes, but in other universities, that big classes, and sometimes just having that one-on-one interaction is really missing the member. When it comes to teaching, we have what we call relationships or interactions. One is the students interacting with content and other interaction is students interacting with students that is like kind of peer interaction that has to be seen in the classroom. The other interaction is teacher-student interaction. That has to be there. When, as teachers are preparing lessons, you say in your lesson plan, by the end of the session, the learner will be able to not learners. So you are not going to that class, even if it's that class of 82, teach 80 you are going to teach one particular student in that class. So the student has to feel that I am relating to the teacher, but now are looking at what is done. It's like you only see people and images and faces on this screen, and it is like they're going through in some kind of interaction. So you find that some of those interactions are compromised. One of the interactions that is compromised is the student-student interaction. So we now don't give them, even if we do group work, even if we send them in breakout sessions, the interaction is not as we really expect in terms of it being a hundred percent. The one on one, each student is supposed to feel that I'm connected to the teacher and the teacher may be addressing to all group of students. That, to me, that interaction is also compromised in terms of content. So those kinds of interactions are compromised in a way, and as are result, then it is like, Oh, there's a lot. Or those kinds of disadvantages we talk of. So it's like they keep students miss each other. It's like, we can't even now go for, even if there is the forum or the platform, it is not reallyreally active forum. Yeah. We just trying to like, copy, copy what is in there face to face and putting it online. So, it's accompanied by quite a number of disadvantages. So, and then if I mention about the challenges, the challenges are many, some students have funny gadgets, very old kind of computers, or some lecturers even have some outdated kind of computers. And you see, there are some letters and software that need to be used. So you find that you don't have access even to some of those softwares that you really need to have. So it becomes a bit of a challenge. Rita, are you there? Yeah.

Speaker 1:

Yes. There was some problem with my headphones, but I was listening to you. So what I've gathered is that due to lack of students engagement, lack of interaction, and then what I, what I thought when you were saying about that when we write it,

Speaker 2:

I haven't said lack of, I've said inadequate kind of interaction. It's compromised interaction is compromised.

Speaker 1:

Yes. Okay. So, thank you for sharing about the disadvantages and the challenges. When we talk about these challenges. Are there any plans to improvise those or to overcome these challenges, especially from a teacher's side or from the student side, because, when we say that students are there behind the screen, we don't know what is going on and whether the student is achieving those objectives, that learning outcome is achieved or not.

Speaker 2:

Okay. Yeah. So the most important thing as I already mentioned is that we will have to continue with a teacher support or faculty support programs, because by the end of the day, there has to be the ownership of, in terms of the classes, the ownership of learning, where are we related to the faculty to really be in charge in terms of how they would really want to teach number one. And then in terms of the ownership in the, on the side of the students will expect that faculty are able to devise activities so that we have, when students are to interact, let it be a session that is interactive. So that's why we say, when it comes to the zoom sessions, the synchronous sessions, there has to be a lot of interaction. So group work is supposed to be done. People have supposed to also realize that it's not just going to class to cover content. It is going there to be able to facilitate learning. So how do we facilitate learning? And we have to really know that you are a caring faculty or a caring teacher for that particular matter. So it is just important that we have the ownnership that is taken care of on both sides in terms of faculty in terms of the students so that you are not worried that they're not doing nothing, what have you designed for them to do? Yeah. If you haven't designed, you want them to just be listening to you. That is something that Is quite boring when it comes to like, they only listen to you as the instructor, what are they bringing? What experiences are they bringing to class? That is what students want to know, because they, they are not stuck with us as they are not empty slate. They want to bring something. What, what work have you designed? Teacher support is very, very important. It will help them to be able to design instruction in a way that everybody is coming to that particular class. As a participant. I sometimes love when the faculty complain that students are not concentrating in my class. When they say that I know the faculty is the problem. The teacher is the problem, not even the students. So that is something that we really wish to work with the lecturees, the teachers, the faculty, so that they are able to know how do I design my instruction so that it is able to bring out learning and the kind of interaction that you would really still match what is done in terms of face to face. So that is something I could be able to mention, go ahead.

Speaker 1:

So it's a teacher's job to grab students' attention in the class and to help them engage in the class, right?

Speaker 2:

Yes. That's why designing of instruction is something that you cannot say that, you know, you don't know. So it's a learning kind of some experience that you need to develop with time. Even some of us who have been teaching for some time each and every session we keep planning, we keep asking ourselves questions. How can I do it differently? How will my students be able to perceive these, what we learn, where they learn out of this particular session? So those are some of the question you have to really ask yourself. But if you want to teach the way you taught yesterday, then students will not be in your session the next week they will go away. Yes.

Speaker 1:

Okay. So, because we are talking about faculty members, it's a very right question at this point that what are the competencies and skills required to teach online during the crisis?

Speaker 2:

Okay. As I said, the way of education is really changing and we can't teach the way we were taught. We'll be fooling ourselves. So we have to do it differently. So they are what we call like a 21st century kind of skills and competencies that teachers need to have as skill. Like intrepreneurship, that is something that teachers should be able to, because our students are not just coming to a class, they want to be marketed. So how are we helping them in terms of developing those skills? If a lecturer, or a teacher is not an entrepreneur in a way, then by the end of the students, the students will have the Paper and they will not have those necessary skills. The other skill is the skill of collaboration. A lecturer should be able to be equipped with the collaborative skills. How do you engage the students? How do you ensure that they collaborate? And that will address the aspects of interaction that I mentioned in terms of how they're able to use a technology in terms of tech savvy, they're supposed to keep improving themselves in terms of technology skills. So that is something that they really need to have. And the other thing is that a skill is that they need to really be able to communicate. So you don't ask students that Students are questioned that they'll go online and Google and give you an answer from Google at then Now you penalize them that they have plagiarized. Your question was wrong. So if you ask a wrong question, or if you ask it strongly, then you expect to get those wrong answers that we get. We would want the students to have innovative skills. So the other aspect is innovation. If not, you don't have to use innovation. You can call on top of creativity. Very few of our faculty could be like creative. That's why I say, don't teach the way you taught yesterday. It has to be different. What innovations are you bringing to class? As a faculty, that's innovation, students will be able to tap into it. And by the end of the day, you will be able to develop those innovative tasks that we are talking about. So those are some of the skills and competencies that would really be required, especially when it comes to people taking technology and teaching and learning for it.

Speaker 1:

So could you suggest some of the ways that can help faculty effectively engage students in the online learning environment? Some of the examples that you did in your sessions to engage the faculty members. So how can they help faculty to engage students?

Speaker 2:

Okay. For maybe Just in short. What I would say is that for every session that our faculty is supposed to do, it has to be accompanied with a lot of planning. So when it comes to planning that tools, that we've given faculty for planning, for example, a tool that we use for lesson planning, like BOPPPS, where you need to have a bridging, you need to do an outcome for your session. You need to say, to do pre-assessment, you need to set up participatory activities for your session. You need to do a post assessment. You need to give us somebody. Yeah. So that's just an acronym, but we are using, like, for example, it's called BOPPPS. And we use that, especially in terms of lesson planning. So that for every session, you look at it and reflect on it through that way. So whether it's online or face to face, the same way of lesson planning has to be done, but you see faculty at higher education would always say that lesson planning is for those ones that ECD at secondary or high school, for that matter for us, we know what we are doing. So you don't do it, but I'm happy across some entities in Aku. They use what we call lesson guides. And those lesson guides have incorporated some of the tools that I've mentioned, especially like BOPPPS planner in their lesson. That is how they can, and then I would also advise, let's say, faculty should they continue to engage in some of the trainings that we offer the workshops that will help them to as an eye-opener, especially in terms of the new techniques that they really need to use.

Speaker 1:

Yeah. Okay. So I think that's too much about how to engage students and how to grab students' attention. Now it's time to talk about the students residing in remote areas or different parts of the country. So what are your views regarding use of online teaching and learning for the students residing in a remote areas?

Speaker 2:

So in terms of remote areas, I know there are some places, especially in Pakistan. Wow. Yeah. Very, very remote. But what we did was that we had to bring in some different, other ways in which they could be able to access some of their content, for example, adding the materials and resources on our flash or flash discs, or USB drives for that particular matter. So that was something that was used. Sometimes you record the lecture and you're able to share with them the recording so that they are able to watch at their own time. But all those ways that we do to be able to demonstrate address remote teaching and learning and compromises on some of the things that I've already mentioned in terms of interactivity engagement, and even when it comes to learning, but you see, we really have to like go over some of those particular challenges. And that's why we had to put in those measures. And sometimes the students have to come on campus or to get to a center once in a while to be able to access good internet connectivity. So it's all like trickled down in terms of how flexible are their students, could they be able to get out and get to those particular test to be able to access some of the things, but, lecturers or faculty here are supposed to already be caring so that they could be also be able to take care of them because we have what we call special groups. And when it comes to special groups, in terms of teaching and learning, it's like, even if you are moving faster, that teacher, you have to move slower because of this special groups. So those are some of the considerations we had to put like a, you need to put in place to be able to get to that a remote planner, just ensuring that if there is a communication, how do you communicate with that learner? And that's why I said, you will have to handle all the students individually so that you are able to address some of their needs because that is what learning is all about. So what I can be able to say in terms of those residing in the remote areas. So at the use of the virtual learning environment, was also very important. Whenever they have some internet, they could also be able to go to the VLE and be able to access some of those recordings of their own. and that is it. I remember at one point in Pakistan, it's like, somebody was sent route to be able to deliver some of those USBs to some students in the remote areas. So it really called for a lot of sacrifice. And that is what it takes to be able to support that remote learner. Yeah.

Speaker 1:

Okay. Alright. So, What are your views regarding complete shift or migration to online modality, keeping everything in the mind that about the challenges and all. What do you still think about it? If we completely shift to online modality,

Speaker 2:

If we completely shift to online modality as an entity or Aku, I think we will be cheating ourselves. Is that too heavy? Yes. At the level at which we are, is that, even if you were to do a complete online or shift completely, it has to be gradual. It has to be after quite a while of time because our students have been faced with a number of challenges and those challenges will still persist for quite some time. And if we shift completely, then we will still just be doing it to justify our online. But in real sense, we will have compromised on teaching and learning for those students. So the appropriate way for Aku would be kind of a blended approach for now where we are mixing the online and the face to face, because shift in that is assuming that the students will be able to access the library without any challenges. They should be able to access some of the resources in terms of the labs and the, the labs that they need completely without coming to campus. So it's like you will have empower the Learner with some of those, which means we will have like a replica of Aku where they are, which is not something that, we'll take maybe 10 years. For me, we can only maybe do some short courses. The short courses are the ones we can now say, maybe we just have them fully online, but for the courses that would have full semester and we embraced the blend, the blend will be quite okay. And this will be the way like we should be able to go for.

Speaker 1:

Alright. Alright. So now we are moving to the next question and it's regarding students' assessments and grading. So what are your experiences of supporting the planning of students' assessments and grading and complete online learning environment during the pandemic?

Speaker 2:

Okay. In terms of planning for this particular assessments. Yes, it's one of the things that we did during the training is that we would work with faculty to ensure that to reflect their curriculum. They should be able to see what are some of the assessments that they will continue with. What are some of the assessments that they'll have to drop? What are some of the assessments that they need to modify? So that is in terms of the training. That is what we really asked them. And they had to design an assessment plan when it comes to their online session. In that assessment, we had now things in terms of how are they going to assess this particular student? Remember some of the assessments are supposed to be graded others are not graded because we are talking of assessment as learning, not just assessment for, or of learning. So if we are talking about assessment as learning, so when it comes to that, the guiding principle would be, what are some of the things that will be graded? They had to make that choice as they develop the assessment. The assessment plan, the assessment was to be that, say, faculty were also to look at their entity policies to see, could they be able to shift, because sometimes we give less attention to formative assessments that is the ongoing assessment in the class, and we give more weight to the subjective. So that, is there a way that they can be able to give weight to their formative assessments and maybe give less weight to the summative assessments, which is that they will only prepare, do our end of term exam. And in some of the scenarios, we will have that, like for example, it kills examinations. It's like, you really need to be in the lab. And some of those ones who are be done with the students are on campus, but shifts who are organized in some of the entities, to be able to have students being assessed in that particular area. But what I could say is that we had a lot of reviews and reflections when it came to the issue of assessment, so that we use it to be able to promote learning rather than using it, to be able to only gauge how the students have been able to really perform. But how are you fitting it in the outline so that it is able to help you achieve the desired learning outcomes.

Speaker 1:

So, just to ask another question that while having the online assessment last semester, did you people or the faculty members have communicated any challenges or anything regarding online assessments?

Speaker 2:

Yeah. When it comes to the online assessment is the same way when it comes to teaching because the same challenges still remain in terms of connectivity, because in some of the assessments, the students who are required to have like two gadgets, some of them could not afford one, to only like what be the screen you're using the other one to ensure that we are also able to monitor what you are doing, you are in your individual location. So for the ability of those kinds of ideas, it was not something that was easy for some of them. So it's like they really had to struggle to ensure that by the time they are doing the assessments, I think that those kinds of, or the examinations, and it's like how we need to, you need to have those kinds of connectivity issues. The bandwidth was also quite a big challenge when it comes to the students and us doing assessment, you'll find that during the assessment. Some of them are disconnected. So it becomes a big, big challenge, also they are also applicable to what happens when it comes to teaching. And then the way I say that we are not just assessing to gauge their performance. We are using assessments to be able to facilitate planning.

Speaker 1:

So, right. So we are left with a few more questions. So now it's time to talk about the recommendations from your side. So what are your recommendations to ensure sustainable remote teaching and learning in the future? I know you have discussed some of the recommendations in the beginning that teachers need to plan in a different faculty needs to perform better when they come up with questions that we are unable to grab students' attention or engagement. Is there any other recommendation regarding training session or anything that you would like to suggest?

Speaker 2:

Yes, my recommendation would be moving forward. There has to be continued support for faculty, especially in this, you know, that those things will trickle down to the learners. Then. the continued support in terms of what they are doing, technical skills, pedagogical kind of skills, those ones. And then we need to continue with their professional development programs, like for example, or what we have in the QTL and those rethinking teaching workshops, the teaching, learning enhancement workshops, those workshops are supposed to continue because they help you, especially in terms of like planning for your lesson, planning for your cause, revising your learning outcomes. Because, we realized even when we were doing the training, is that some of the learning outcomes were outdated, so you would even challenge the faculty that can you revise your learning outcomes. So those professional development sessions are very, very key. And then the other thing is that we will still continue to have a very, very working relationship, especially when it comes to the QTL. That is the quality teaching and learning network with the rest of the entities. And the entities have to be open, especially in terms of bringing out some of the challenges or some of the issues that need to be addressed, because the QTL is well equipped, especially in terms of some of the current trends, some of the current issues some of the current issues in as far as teaching is concerned, then the other thing is the aspect of moving to a next level where we are taking collaboration at another level where our classes are able to collaborate. That is something that a faculty should take forward. If I'm teaching, I have a class in East Africa that is on microbiology, and there is another lecture in Pakistan, teaching microbiology, even in the same, same semester, then how do we ensure that our classes are collaborating? They have even some similar assignments or tasks that they need to do. Even outside of class, some projects they can be able to do together because the way of education is project or a problem based learning. So that is something that we really have to embrace in terms of moving this kind of online teaching and learning because when students collaborate on projects, even if they were disconnected in terms of online teaching, or they were disconnected somewhere when those online sessions are on, but when they are working on a project, they still have to stay connected. So why don't we give them the projects so that they can be able to work on those projects? It can be a, between countries or between classes or between, campuses. That is something that is where online learning is taking us so that we are able to embrace collaboration.

Speaker 1:

Right. So two aspects that you have talked about. I have noted that down. So another thing is that, how do you see university support or university role in executing, remote teaching and learning program? So you did talk about the entities, which are working over at different departments who are supporting the students and the faculty members. Would you like to add on to this particular question, otherwise we can move ahead.

Speaker 2:

So maybe as QTL, what we are doing is that we are really trying to harmonize this kind of online support. Like if you look at the entities, what you've done is that each and every entity has what we call a VLE assistant, somebody on the ground who is able to help and work with faculty on their VLE. And those are the people we work with them. We do give them workshops. We have a VLE assistant workshops that we ask. We normally schedule like last year, we had about four sessions with them to just introduce to them some of the new things that they could also be able to share with faculty. We also empowered the VLE assistance to be able to lead, take the lead when it comes to the the orientation of students. So we only come in as BDL or QTL to be able to support their orientations, to be able to support them. But what we do is that we sit with them, we have those sessions and we are able to like empower them so that they have those ownership. So the way of teaching support is like on a collaborative kind of an aspect, a collaborative point of view, where we are now able to really collaborate so that we able to promote the best practice. I know entities would be working on their different agenda or things. But when that is harmonized, when it comes to support, we are able to support faculty across. So that is it. We will still continue to support them at working with those people or the divisions on the ground.

Speaker 1:

Okay. All right. So we are on the last question and it's about the SONAM. So how SONAM can be trendsetter or role model in introducing remote learning program to meet countries, nurses, demand and healthcare system.

Speaker 2:

Okay. Okay. Well, some of them, I think I've worked with the instructors in SONAM, and I know that we can tap into their abilities. One of the things that I noted is that they are quite competent in what they are doing, and they are also committed to their tasks. So to empower them is and SONAM being a change maker is how can now SONAM be able to link up with the rest of the entities, because you can't say, it's, it is independent. How are they linking up in medical college'? Are their sessions that they have with the medical college, then how are they linking with the communities? I know we have like medical centers or, or those centers in the country, but how are, is SONAM linking up with those ones in how is SONAM linking up SONAM Pakistan, linking up with the rest of the SONAM's in the world, like Uganda, Kenya, SONAM Tanzania. If they haven't developed that kind of bonding, as I said that, let them now do like team-teaching teach classes, teach some classes in East Africa, if you are teaching, like for example, health assessment in Pakistan have a session even if it is a one hour 30 minutes session for East African students, so that you are able to link that let's see how are those students bring up similar or working on a particular project with those students in Kenya. So if they are able to design those kinds of collaborative activities, then now sort of we'll be able to have taken it's right place in the area of nursing and the area of education. So that is something that, if they want to be changed, because that is how they're going to start initiating change so that we are able to see that they are not only confined in the boundaries of Pakistan, but they are taking whatever skills they have across. We would want to see some of them coming to teach here, even if it is like for one month, two months, face to face, with the Kenyan, we have the Kenyans going to Pakistan, teach for one month. Those kinds of experiences will be able to put a sort of, as a change maker, because I remember in some of my trainings, I was in charge of SONAM, Pakistan, and I train them on the use of VLE from East Africa. So, and it had a bigger impact than if they were even maybe trained by somebody from Pakistan, or they took it up very seriously and I'm so happy with the success that has been realized in SONAM. And we have a very, very good working kind of relationship, it doesn't matter this kind of a black man coming to teach us, but it is like they really respect what I really had to offer in terms of training.

Speaker 1:

Yeah. So, that's all from my side Sir , and thank you so much for taking out time for this interview. Is there anything that you want to say or ask?

Speaker 2:

I never imagined that this session would take one hour and it has taken one hour. I thought I would take 20 minutes at another. Is that all your questions, but I'm so happy. I look forward to having the recording of the same. I also look forward to share it in your research write-up. I also look forward to translating these into another project that we could also be able to do so that we, I am also able to incorporate, to be incorporated in the project maybe as one of the leads, and also be able to challenge our faculty. I look forward to also, being part of the online classes in SONAM. I can come in as an observer so that I'm able to observe some of the lessons and be able to share some of the lessons learned out of the sessions. Yes.

Speaker 1:

Inshallah and we'll soon, share the findings about this research with you. And, if there is anything that you find that you missed or anything like that, you can always write us in a form of a reflective log on the similar ID from where you received the zoom link. And thank you once again for taking out time. So take care

Speaker 1:

Thank you so much and say hi to the team. They all know me just say hi to all of them. Thank you. Thank you. Bye bye. Take care. Bye

**IIKi 4-Date**

**Interviewer: Okay. So, my first question is, what are your views about online teaching and learning?**

**Interviewee:** Are you asking me this as an educator or being the administrative head?

**Interviewer: As a Head person and dealing with all other students and teachers feedback and everything. Is that right?**

**Interviewee:** Yes. So to me, online teaching and learning or online education is a more through which one is unable to see the student, physically and are connected through any available online modality where a teacher and a student is making educational contact. So for the purpose of study and for the purpose of education, a teacher and student are connected online. It is one of the recent contemporary modes of teaching and learning where distances physical presence is not a barrier to higher education or any education. So it is a flexible approach that enables anyone to pursue education through any institution that they want to. And being in the same city, being in the same campus and being close by is not a barrier anymore.

**Interviewer: Right. So according to you, it is a flexible approach where a student and a teacher can interact and work alone. So, my next question is that what are your experiences of supporting the migration of existing curriculum to remote teaching and learning during the pandemic and how can faculty and students be supported in this transition?**

**Interviewee:** Okay. So, can you read the first part of your question again?

**Interviewer: Yes, it is. What are your experiences of supporting the migration of existing curriculum to remote teaching and learning during the pandemic?**

**Interviewee:** Okay. So, my experience in supporting was that when COVID struck and the higher education commission had strict regulations about not having physical classes ensuring student safety, we as a university abided by those rules. And first of all, when we said that we have the permission of going online, as faculty members, we were all prepared because we were already teaching on the blended learning mode and we had the skills and knowledge necessary to pursue online education in the full format.

So the faculty was ready, but the experience in terms of student access was the biggest challenge that I had to face as the head of the program, a very high percentage of our students live in the Northern areas and getting access for them and getting in contact with them was a challenge. So, we had the courses ready, our faculty was prepared, they were trained to put up the courses. We had somebody refresher courses done for the faculty members. We arranged for the resources for faculty to record their lectures, to add videos to their lectures and prepare online teaching modules. But the students did not have access. So, in terms of specially the four-year BSCN, and are you focusing on the four-year BSCN or o RN program or both?

**Interviewer: Post RN.**

**Interviewee**: Okay. So in Post-RN very few students did not have access. And what we did was that we called up every student on the telephone and we emailed and used a snowball networking to find out if the students had internet. And if they could connect online for the students who did not have internet, we offered them that, you know, the university could pay for some data bundles. If they thought that they could buy the data bundles. And if that was not an option they were assigned to an Agha Khan education services school in nearby areas where they were assigned days and timings, and they could walk to those schools where internet and a computer lab was available for students to use internet as well as the hardware, if they did not have a computer or a laptop at home. So this way we were able to connect those students post RN’s, may it was not too big an issue because most of the students had internet connection as they were belonged to Karachi and people who were in the North, but either working or employees of AKU or somewhere else where they could go and use the internet. So, that was, how we started. Then we started teaching online. The students came regularly to the classes we had, online teaching, both synchronous and asynchronous, where, we had zoom sessions where students could interact. And there were times when students had to do self-directed learning modules and answer on discussion forums and post assessments.

So, it was a new change for the students, and they enjoyed it. The teachers were learning, the students were learning. The assessments were also new. The teachers had to learn how to give complete assessments in an online modality. So we were trained on teaching in a blended format, but assessments so far were done in the traditional way where students would come to the classroom and attempt their exams and the scholarly papers and other presentations were also face to face. So, this time it was where students were on either zoom or they had to give an online exam. And, they could still submit their scholarly papers through email. So, these were some of the experiences. And, and when we started to do these things, there were hitches where students could not connect continuously. They had interruptions, they were not happy with time bound exams because each MCQ that they had to attempt was timed and they had to speed up, whereas in face to face, they are kind of, you know, used to having a time where you, when you distribute papers to the whole class, they get extra time to read. When you are submitting the papers back, they get extra time to read, but in a virtual environment, the computer shuts and goes off. And this was a kind of anxiety provoking for the student and they were kind of getting used to all this.

And we also had backup systems where we could check if the student did face genuine technical issues, we supported them. if they were technical reasons, we could offer them another exam, another paper, or we also allowed them to come to the campus and use internet services for their assessments, because they were a small group until then HEC had allowed people to come in very small groups. So, these are some of the important areas that we were able to work on.

**Interviewer: Okay. So, you have very well explained about how you supported. I just want to ask that, uh, how can, like, is there any better way to support this transition or are there any further plans, if God forbid, if we go back to the same thing again, or if any, such other pandemic happens. So any other planning?**

**Interviewee:** The planning would be that this time we have, we would tell the students that they have to make a conscious arrangement of where they are going to study and how they are going to study. So, they need to be prepared with their own device, with their own internet. And if they need any financial or moral support in determining that we are there to help them. But as university students, I think they also need to be cognizant that this is their learning and they need to be responsible.

If the university's allowing them to come in smaller groups, people who really need internet support and do not have it at home should come ahead of time, placed themselves in an environment where they are safe, but they can get good internet connection and offer their exams and tests. So, it has to be a pre prepared thing because the students have been through it. They know what kind of issues they can encounter. As a faculty, we know what kind of issues we can encounter. So, everybody needs to have a better backup plan this time.

**Interviewer: Right. So, let's move to the next question. And the next question is that what are the advantages of online teaching?**

**Interviewee:** So, the advantages are, like we said, it's flexible. You can be anywhere and get connected. You can work and get connected and do online learning and reading in your own time because it's on the web. You can always access it based on your convenience. On the other hand, I think it is little easy on cost also. Where you are spending on Wi-Fi, on the other side you are saving on transport money because you are doing all the working from home. 24 seven time schedule available here for you to respond. And even for the faculty, for suppose if they are busy in something during the day time, so they can respond in the evening if they are free. maybe after hours they can connect with the students, the weekends are also available, sometimes it is difficult for students to connect so they have time flexibility, resources flexibility, learning flexibility are some of the advantages of online learning.

**Interviewer: Okay. anything about balance with work? Family? anything regarding that?**

**Interviewee:** So, you know, like I said, you can balance your job and your study, you can balance your home and your study and if you have children and family responsibilities during the day, you can handle those and do your studies in the night. That could be your preference time.

**Interviewer: Okay.**

**Interviewee:** So, for the mothers especially when the kids are in school, they want to study, so they could utilize those time slots for studying purpose.

**Interviewer: And what are your thoughts about disadvantages of online teaching and how can these disadvantages or challenges be mitigated? So first you can talk about the disadvantages and then we can move forward.**

**Interviewee:** So, disadvantages are few, so a physical and face to face eye contact is always missed by a teacher and learner. When you look at each other's in the eye and you do nonverbal gestures like nodding and smiling, it is fruitful for a student to see that the teacher is engaged and it's fruitful for a teacher to know that the students are learning and, are kind of connected, non-verbally with the teacher, which is not available online. Even in zoom sessions, you can't keep an eye on the whole class. So, that's there. One is not even sure that they in any session the student has logged on and is there or has gotten busy with other things that are there.

So, keeping the student engaged is a challenge and a disadvantage, and that needs the teacher's preparation to prepare the class in a manner that student engagement is ensured and learning outcomes are met. The strategies are placed in a way that there is a, you know, there is online student engagement that are small learning outcomes spread in a bigger time, so that you keep assessing the student.

As if like if there has been a session of two hours, then as a teacher you would have to include some strategies to enable you to know as how much the student has grasped and as how much the student could study. The pre reading and the reading material has to be, user-friendly not very long, not very boring. So that when the student is reading it from the screen or when the teacher is not there. So, it is easy for them to read and understand as to what is expected of them. The guidelines have to be clear, because there is an opportunity in the class that you can raise your hand and ask a question on the campus with some other student or teacher. So, online many possible questions that the student may have, should be clearly put-in, as clear as possible guidelines for the student goal in the class participation, for assignments, for the assessments and everything. The grading has to be prompt. The time schedule has to be maintained and as where and at what time, with which pass-word it has to be logged -in. And till when and what duration the exams would be done. There needs to be a to-do list of everything as which you do in the face-to-face interaction. It has to be written down in the websites for students to use.

**Interviewer: Okay. You have answered my next question as well, but I will repeat that question. If there is anything that comes in your mind, you can talk about that. So, what are the competencies or skills faculty members required to teach online during the crisis? Earlier you said that your faculty members were prepared to go online, and then now you said that they should know how to engage students as what the interactive way should be. So, are there any other skills or competencies that a faculty member should require?**

**Interviewee:** So, yes. the capacity building around online teaching. So blended, we were well-versed but how to do the complete the things online without meeting the students at all. So, teaching online is one, assessing online is one, and then grading and sharing those grading and feedback with the student is one thing. So, our faculty has to be very versed and expert in doing all the three phases. The queries, concerns of the students as how and when are they going to be available, is another thing. So, to ensure online engagement is an area that all faculty members will need to be well-versed with. And, then to see as how to manage each and every strategy as how to have the pre reading done or as how to give the guidelines as it comes in the literature as how do you prepare a valid and reliable and a non-Google able assessment strategy in your online teaching? So, the teaching has to be done in a manner that the learning outcomes are met, and the assessment has to be done in a manner that you are able to assess as how the student has read and understood it. And as how to grade them by making sure that the objective has been met.

**Interviewer: Alright. And as you also mentioned the effective ways. Are there any ways that are in your mind, or like how can a faculty effectively engage students in the online learning environment?**

**Interviewee:** So, the faculty has to be aware of the various strategies like as in what kind of strategies can be in that sort of topic, and how the student can be engaged. For example, as we give a break in between a session and when after the break we recontinue the session and the students are seen online in the list, there should be a small check done to see that all the students have returned by having them to raise their hands. So if someone has just logged in and gone he / she will not know that you have asked them to raise their hand, so you have to be prompt and energetic yourself to be able to see as what the students are doing by asking random questions and then once or twice asking the students to open their cameras, to see if they're there or not, and they are enjoying or not, invite any questions or queries, give simple instructions, give and teach, not for very long hours, but ensure that the students concentration is maintained for 20 to 40 minutes, and then an exercise is done to keep them busy and not let them get bored.

**Interviewer: And as also using the features effectively as you had spoken of the raising of the hands. You should know all those features. Okay. So, now moving to the next question and it's regarding the remote areas. So, what are your views regarding use of online teaching and learning for the students residing in remote areas?**

**Interviewee:** So, for the remote areas remote strategies would be used. Where study packs are sent to the student or the study packs can be sent to a centralized place from where the student can pick and then submit it there too. So just as there are strategies for online and blended learning, same way there are strategies of remote teachings, also. So again, the principles are the same that as your teaching material has to be precise, clear, readable, downloadable, printable. So, all these options should be with the student. The assessment should be made in a form which would not be directly available on Google. And the application or comprehension should be on the level of the synthesis and analysis which ensures that if the student has not pre-read and understood then he/ she would not be able to answer it. The way as how to answer and how the guidelines shall be done, then the marking criteria, rubric. Everything has to be very clear for the student to understand and attempt, and then submit on a point where from where a teacher can receive and correct it.

**Interviewer: Okay. So, can you please share some of the experiences that happened with the students who were residing in the remote areas in pandemic times?**

**Interviewee:** So, there was an experience of this in the pandemic times but not with the post-RN.

**Interviewer: Okay.**

**Interviewee:** The post RN were either able to connect online directly or indirectly but the study packs were not sent to the post RN.

**Interviewer: Alright. So, were all residing here in the city?**

**Interviewee:** Most of them were in Karachi. Or they were able to get access on internet through schools or through neighbors or somewhere through WhatsApp or their own phones.

**Interviewer: Okay, okay. Okay. So, the next question is that what are your views regarding complete shift or migration to online modality, even if the pandemic gets over and after that?**

**Interviewee:** So, it is a good idea, especially in the post RN BSCN program where most of the students who want to join post RN are working somewhere or the other or are raising families. So it will be a very good idea to go online completely, but they really have to be strategies planned to make sure that because this is a healthcare degree, it will require a lot of clinical hands-on work. So, somehow how this online work will be adjusted, will have to be sorted because it's an important component of higher education in nursing. So strategies like simulation and strategies like as if I am working in Chitral or Gilgit somewhere, Or if I am in America or any other place, and I want to pursue online then I should have one such option where then an hospital can be identified as where there is availability of preceptors. So that my teacher could be online facilitating me for a clinical component, and I have a preceptor on ground as from which country or city, I am working from. So, working with those connections and working with that preceptor, and that I can work as a trio model of faculty, preceptor, and a student.

**Interviewer: Okay. And what about the resources? Do you think that, the Institute is prepared enough to further this idea?**

**Interviewee:** For post-term BS CN? Yes, we are prepared, and we just need to finalize the processes because we have already piloted it on a few students who were not able to come back and work here. So, there is a case study where a student was in Canada and she had a preceptor there and we had facilitated her online and had her practical done. So, for post RN, I think we are ready. The student needs to be ready and be available and willing and to take these flexible options.

**Interviewer: Okay. Thank you for the answer. Okay. The next question is regarding assessments and grading, you discussed about some of the experience, but I would like to repeat if there is anything left, or any other thoughts, please share. So, what are your experiences of supporting the planning of students assessment and grading in a complete online learning environment during pandemic?**

**Interviewee:** So, I think I've covered most of the points where I said that, you know, the guidelines have to be such the question construction has to be valid and reliable. The timings, the guidelines, as briefing to students about what to expect. I think these are some of the major areas that I've already talked about.

**Interviewer: Okay. And were there any issues that were reported during the pandemic time regarding assessment and grading from students or faculty site? Any sort of challenges?**

**Interviewee:** Yeah, there were challenges, we needed a system building. So, they were, first of all, the students were, some of them were really surprised at how they were not able to gain good marks. And when they looked at the questions, they realized that, you know, probably they didn't give attention to what readings were given. Some of them faced technical issues. And if we were able to trace technical issues, we gave them another option of doing a retest or the re exam to be fair. Some of them did not report technical issues, but when the results were shared, they came up saying that they had technical issues. And, but we did not entertain them because if they were told to report it then and there, and they did not do it, then it was not taken care of.

**Interviewer: Okay. Right. This is a general question regarding the challenges that you faced in implementing online teaching. So, keeping in mind assessment or grading or any other like, well teaching and learning process, were there any challenges that you people faced in implementation?**

**Interviewee:** So, connectivity issues, electricity issues, which are part of our context, were at the top. So because the students, when they started the program, they were not ready. They did not know, the teachers did not know that COVID is going to come and we will have such issues. So, on the first go preparing to teach online was a challenge. But I think as it went ahead, it became better and systems were kept in place. And the virtual learning environment was strengthened. The resources were provided. The student got used to connecting to the teacher without meeting them. And I think with each experience it's getting better.

**Interviewer: Okay. So, we are left with a few more questions. So, what are your recommendations to ensure sustainable remote teaching and learning in the future?**

**Interviewee:** Like?

**Interviewer: The guidelines and all, any other thing that you think should be recommended?**

**Interviewee:** I think our whole guideline at the university level is going to be helpful and we are making it too. There had been a group made for remote and online teaching. For which we have guidelines and for which a manual has also come which guides the faculty online teaching strategies or choosing use on each idea. So, I think when such resources are on a university level then avenues would be made where the students will be able to borrow hardware and internet from the university. So, I think the systems are made then all these things would be more sustainable.

**Interviewer: Okay. And was there any model that you people followed for online teaching and learning?**

**Interviewee:** No. It was either a normal curriculum model, which was, which is on the paper was translated into an online format.

**Interviewer:** **Okay. So, the next question is how do you see university support or role in executing, remote teaching and learning program?**

**Interviewee:** I think I have answered that too, a bit.

**Interviewer: Right. Okay. And the last question is how Sonam can be a trendsetter or a role model in introducing remote learning program to meet countries, nurses demand in healthcare system?**

**Interviewee:** I think the answer to post-RN would be that we go completely online, and we invite people at the time of their admissions to identify a placement and a preceptor to whom they will work from. And so, this program could be strengthened so that wherever you are in the world and if you have a clinical placement available with you and that you have an eligible preceptor which we can make as your criteria then the practical and theory can be completely taken care of as where you are located in, you need not come to the campus online or need to campus at all.

**Interviewer: So, like, do you really see Sonam as a trendsetter or like it takes, it will take some time, some years to become a role model?**

**Interviewee:** It said, I think the ground is set and we have been marketing it. And we do it for our students too that integrated clinical objectives can be made and if one is an employee and he / she wants to work from their workplace then we do have the flexibility. We have tested different models where the students keep working as staff nurses, and we give them additional objectives and additional hours to work on a few things and produce assignments and discussions where their competency could be increased and they could do a work based program online.

**Interviewer: Okay. I think that's all from my side. And thank you so much for taking out time. Are there any questions from your side?**

**Interviewee:** No, I think I'm good.

**Interviewer: Okay. Thank you so much. Again, take care. Nice talking to you. Bye.**

**Interviewee:** Likewise. Thanks. Bye. Bye.
