# Supplementary material for: Beyond the classroom walls: Stakeholder experiences with remote instruction in Post RN baccalaureate nursing program during the COVID-19 pandemic: A qualitative inquiry
Source: PLoS One. 2024 Apr 4;19(4):e0300007. doi: 10.1371/journal.pone.0300007 (PMC10994296; doi:10.1371/journal.pone.0300007)
Supplement: S4 File — (DOCX) [file pone.0300007.s004.docx]

**IDI – January 11, 2021**

**Interviewer – Ms. 1**

**Interviewee- Ms. 2**

**Speaker 1:**

Okay. So my first question is, what are your views about online teaching learning?

**Speaker 2:**

So online teaching and learning was perceived to be difficult before the COVID-19. And there were opportunities for online teaching and learning on the campus. The QTLNet was doing a lot of capacity building workshops in order to train the faculty members for online teaching and in their bootcamp. And they were basically aiming to train our faculty to equip with new technology to use the new technology which is associated with teaching and learning in their classes. But it was observed that most of the faculty members were also not accustomed with the new technology. And we were, all of us were actually dependent on the traditional methods of teaching and learning. So no one has thought about that actually whatever we are doing face to face right now can be done online as well. So when you talk about online teaching and learning, I would say that it was perceived to be difficult. People used to think that it is not my cup of tea or, people used to think, okay, we are out of it. I mean, we don't have to do it because we are blessed that our students are there face to face, but when COVID came, it became a necessity, it became a must do thing that everybody has to do. So at that time, many of us were not confident that how it will happen. So this was a challenge when, when we talk about online teaching and learning. So, whenever the term online comes so, it will be a difficult thing, but now with time, people are adjusting with this. And, I must say that university has tried to give a lot of support in order to make a strain and make us comfortable in using and teaching online.

**Speaker 1:**

All right. So as you shared that earlier, it was assumed that it might not be required or it is always seen as a difficult thing to do, but after COVID-19 it has become a necessity. So my next question is that, what are your experiences of supporting the migration of existing curriculum to remote teaching and learning during the pandemic, and also how can faculty and students be better supported in this transition? So you can first share your experiences, and then we can switch to how can you better support this transition.

**Speaker 2:**

Okay. So the first question you asked that what was my experience regarding online migration? Right,

**Speaker 1:**

Right. Okay. Existing curriculum to remote teaching. Yes.

**Speaker 2:**

So, actually I am representing the curriculum committee at SONAM what happened that as the COVID started, our students were sent back to their homes and they were living in remote areas across Pakistan, almost 60% of those students were not accessible to us. And they were in the mountainous region and remote regions where internet connectivity was not there even some of them were not able to connect through WhatsApp or Microsoft teams which can easily be used on 2G as well. But our students, some of them belong to such a disadvantaged group that such a simple 2G device with the connection was not available to them. So what university did was that, first priority became, we were in the middle of the semester, right? So there were plenty of assessments that were remaining and the curriculum also had to be taught. So what the university did was that they tried to establish the hub and spoke model, in those regions where connectivity could be established with the help of the Aga Khan education services schools. So one thing which the university tried to do, and students were asked to access the internet through those hubs which were AKESPs schools. So one thing was taken care of like this now, but with regard to the curriculum. So being here, our responsibility was to migrate everything, including the teaching, as well as the assessments to the online modality. So that was quite when it came to us, it was quite hazy. We were also not familiar with that, how this transition will happen. So what we did was that we connected with the QTL net first. And then we try to understand that how, and what are the best practices for online assessments and online teaching. So that meetings, a couple of meetings took place. And then we come up with our faculty needs assessment and their apprehensions for online migration. So we conducted a need assessment first in which we received the responses that majority of them were apprehensive with the online migration. And most of them were fearful that probably they are not having the skill and not the appropriate devices to actually move in the new direction. So that those were fears at that time. and this was conducted in August 2020 at that time, that quick survey gave us an idea that majority of them felt that they were fearful, but almost 40 to 50% were also thinking that it is a way of opportunity. And we should, it is now on all of us to acquire new skills and to become accustomed with a new learning and cope with this, because this has to be done. So, we also found that they had, they were positive to learn, and that actually, their positive attitude actually helped us to conduct an online assessment, a workshop. So with the help of QTL net, we designed an online assessment workshop for the entire faculty, SONAM. And then that, what we did was that we had invited several facilitators. One of a few of them were also from East Africa. And one of them was our visiting professor from university of Saskatchewan. And we had facilitators from QTL net and our SONAM facilitators. So it was a multi disciplinary sort of a group that was conducting this workshop. And then we delivered this workshop in order to sensitize and to share the best practices for online assessments. So this was a readiness exercise and preparation exercise for the entire faculty members. And then we also received a lot of feedback on this step that what were the fears and now how they are feeling about their own competency and skill regarding the online assessments. Then we give time to the faculty members and then the revised guidelines for the assessment and planning in line with the HEC guidelines. So we also looked at the HEC guideline and we also looked at the best international best practices guideline that can actually help our context, which was no bandwidth and poor connections. So that could take care of both the things. And then we developed a new guideline and we gave them the new guidelines set to all the faculty members to revise their assessment plans in the current situation. So the faculty members revised it and they brought to us, they submitted the revised assessment to us. And then in the curriculum committee, based on their judgment, based on our context student need and students access, we gave approvals to the modified assessment plan. Great. So this was the entire story that how we migrated what principles we thought about. and definitely in our mind there was always the principle of access. equity and quality. We were completely thinking about all these principles. We have never tried to compromise on any of these principles while we we were doing the online migration.

**Speaker 1:**

Right. So, like the workshops you did with the help of QTL net and, and other like departments in order to support your faculty members and students, is there anything in your mind or within your team that is going on that, how will you be better supporting for suppose if this pandemic remains that like, unusual circumstances remains like this, and we need to be still teaching students online, then how will you be better supporting your faculty members and students, especially those students who are living in remote area, as you discussed in the beginning that I think more than half of the class or 60% of them are in the remote areas where internet issues are there, power failure is very common, even in Karachi. So is there anything that you, people are planning to better support?

**Speaker 2:**

Yes. yes. So, when we were trying to migrate on these so we had developed certain principles that we will not keep any of the assessment that requires, which our students cannot do actually. And we will not give the strict timelines. So, for example, if previously, when students used to be in hostel and when they used to be in face-to-face time, then we used to have very strict deadlines that, okay, if 15 is the deadline, so deadline means deadline, but when we were doing this migration and considering our students access and their remote situation. So we had also asked them that you can accept a few late submissions if it is within three days. And then what we also did was that few of the assessments had to be done through our face to face viva exam. So, those were the challenges and in that face to face there were certain examinations that in which we had to see what they are doing actually. So if they are checking the eyes, if they are checking the nose, so I have to actually see them how they are doing it. So those were our challenges and problem was that we couldn't say them that, okay, make a video and send me because the bandwidth was issue. So how could we support them? So, we had asked our faculty members to be creative as much as you can, in order to complete both of the things that their objectives must be complete and the assessment also be complete. So we had kept our faculty members open, and sometimes we thought that probably to me be impossible that due to the poor bandwidth, we may not be seeing everything, but our faculty members were so creative that they themselves extracted a solution that instead of asking them to submit their video, we are going to ask them to share their photographs powerpoint photographs, which is limited to these number of slides. So we are not exceeding the MBs requirement. And simultaneously we are also looking at their skills and, and assessing their skills as well. So this was our principle that we are not going to have a very strict timelines. And we will give flexibility to the students who are having difficulty accessing internet. Then we also were flexible that if you have any creative ideas, so please don't keep it to yourself and share and share in the curriculum committee. Then we had also discussed how we are going to Mark on the assessments, which are actually time bound assessments. And in that we have to assess their recall knowledge. So how we are going to do that. So for that as well, we allow the students to have password protected documents, and then you can submit the short question answers to us, and then we are going to accept that. So we have given our students and also that some of the students after doing everything, they were unable to give us tape recorded, video recorded in some of the assessments when they were not able to give us the video recorded, then we also became flexible to take their, for some of the courses we have also taken there telephonic viva. So, flexibility throughout was a key keeping in mind that we are not losing any course outcome or the learning outcome. And we are not losing any of our quality of our assessment. We are not compromising any of the rigor of the assessment, managing both the things were very, very difficult, but we have ensured that when we are flexible, we are also ensuring that we are not compromising the reliability and quality of the assessment as well, and teaching as well,

**Speaker 1:**

Right. So, good to hear that keeping quality in mind, and also a student's perspective, you people remain flexible so that this unusual times become easy for everyone, especially for students. So moving to the next question, I would like you to share your views regarding advantages of online teaching. So, over to you.

**Speaker 2:**

So, if internet is available, if it is completely an innovation of teaching/ a revolution of teaching, I would say. So, many of the aspects that I have noticed regarding the online is mostly face to face. and they were too much dependent on their faculty and they used to depend on the PowerPoint and slides and everything. But now when all the resources are with them, the repository is with them. So they know that they have to do it. So they are coming in classes prepared that I have seen they're coming in, classes prepared, they're coming for clinical prepared. They're coming for their skills, prepared, everything is prepared. And they know that if I miss anything, if I will miss that class, I will not be able to apply theory into practice. So it is making them more responsible. Number one is that I think if we follow all the procedures, then it enhances the rigor of your teaching, because then now what previously, what used to do, I used to deliver a face-to-face lecture and then students used to forget everything, but now what is happening that students are requesting, okay, ma'am, can you please record the session for us? So if we don't understand, at least we can go back and we can have this. So this is also benefiting our students that they can, they can rewind, and they can at the time of examination, they have too many queries as they used to have before.

**Speaker 1:**

They can revisit it.

**Speaker 2:**

Yes, exactly. So, what online education is also giving us an advantage of having lots of activities. So we are able to.. okay. So, once we are done with the class, okay, so let's have a post-test or let's have a review, so I can give them quizzes and they can keep playing with the quizzes and, and they can review. Okay. So how I did, so it is also helping them to become prepared for their summative assessment. So the opportunity to, for the formative assessment has also increased, which due to that time bound of the face to face class, we were not able to do as much as we could. but online is giving us an opportunity to, to play with the lot, many formative activities. so sometimes even I can say that, okay, so today I am not teaching it directly, but there will be five activities that you have to do. And that will allow me to complete your hours and the objectives will be achieved if you do these activities. So it is actually engaging the learners more as compared to the class face-to-face. So , when I was not able to engage all 70 of them, but sometimes it is, if I do this way, so everybody's, if everybody's doing the activity so they can get the opportunity for them, for, for the engagement becomes more. So if everybody's doing and everybody's reading and then doing their activities, so I will Mark their attendance on the basis of their participation and activities done. So, so this is also the advantage. And what else? I think this is, this is all for my side.

**Speaker 1:**

Okay. So what I gathered from you is like, online teaching is more engaging because, even as a faculty member, she can use various tools to have formative and summative assessments can be done online. And then you said if internet is not a problem, then, online teaching is possible any time. what, I would like to talk about like according to the advantages, does it have any relation to study routine or place of study? Would you like to talk about these areas?

**Speaker 2:**

Okay. yes. study routines, if you completely asked them to, to move to asynchronous, then I think learner become sort of careless in, meeting the timelines. And so...

**Speaker 1:**

So, you think it's a disadvantage.

**Speaker 2:**

Ya, it shouldn't be completely asynchronous for our students. It doesn't work actually the level of learners that we have. So, yes, they will be able to, but, but you have to really grade it, if you want to have the task done, then you have to grade it, then they will do. But if you say that, okay, this is my class today's class, and you have to complete all these activities, and this will be counted as your class participation. And the attendance will be counted based on that then five out of 70, will do it. So that's disadvantage. So therefore we encourage synchronous mode.

**Speaker 1:**

Okay.

**Speaker 2:**

Yes, please. Go ahead.

**Speaker 1:**

I was saying, we have started discussing about the disadvantages. I think it's the right time to ask you about what are the disadvantages of online teaching in pandemic situation and how can these challenges be mitigated? So if we can talk about these.

**Speaker 2:**

Okay. So the disadvantage number one is poor access students are disadvantaged a lot. And sometimes those who are having difficulty with the technology use, they also face challenges with the online teaching and learning. So for example, when I was teaching, and then it is also good that everyone has their own devices. So, our learners also have the habit of having a peer sitting besides them. And if they don't understand anything, then they slightly go and whisper to that colleague instead of asking the faculty member. So if the COVID has brought online modality, it's fine, we have all migrated, but our student mentality has not migrated. And if during that time, if that, learner who is a shy student who is struggling and who had the habit of whispering and the peers’ ear next to so that student is still struggling and, and how we encounter that learner when they come for a brief encounter during clinical. And then they say, okay, miss I missed this concept during online, and online is not good. I couldn't study online. I don't want to study online. So it has brought a lot of challenges when some, for those learners who were too much dependent on the face to face mode. So, it depends on the learner motivation actually. What I feel is, and there are some learners who don't want to change, but they need a constant reinforcement that there is no solution. If you will not cope with it, then you will lose everything. So I thought that this is one of the major disadvantage that I have encountered. You already talked about place of studying. So, yes, this is a challenge that our students encountered because although they were living in the urban areas, but, in our difficult living situation, so in one or two rooms, maybe seven or eight, family members are living. So, and when for, for one or two seconds of their mic is unmuted so entire, you will feel that something at the back lot of people are there. So it made me used to think that how that learner will be focusing and concentrating on the lectures. So that was a real concern.

**Speaker 1:**

So balancing the work family and responsibility was, um, like a disadvantage for a lot of people who think that home is not a conducive environment to study.

**Speaker 2:**

Exactly. And, then we had poor access issue, which I already talked about that, the load shedding, and poor connectivity. So they requested me that if you record your sessions, then even if we get disconnected, it will be easier for us to go back and review. but for example, if I'm doing an activity in the breakout room and the learner is actually going through a process of learning and what we are doing in the breakout room was an important objective which was important for the learner to understand that at that time, so that, that cannot be learned through recording that could be done through practicing. So that was also a problem that connectivity, if it is lost, then important objectives can also be compromised for that particular learner. Then, all the objectives couldn't be taught online that I realized, during my last few classes that previously I used to teach that face to face. And in that particular class, I used to Mark, take the scenario and Mark on my examination sheet. So, and then I use my learners to save to I wanted my learner to show me where they are marking. So I used to have a round in my class and I used to, Oh my God, I pick this up. Now you have marked it incorrectly. No, no, no, not here, go here. So it was easier for me to, to see where they are going wrong, but now when they are remote and most of them do not have the webcams. And if I allow all of them all 70 to open their webcams, my connectivity is gone. So, and the bandwidth is, becomes affected. So all the objectives cannot be done online. And it is very difficult to at times, ensure that all 70 are active and alert and they are engaged in the process of learning. It is at times very difficult to say that, out of 70 we only hear the voices of 20, but it is really, we don't have any idea of where those 50 are...

**Speaker 1:**

Is it like having large class size on online platforms and handling them is not easy or not possible?

**Speaker 2:**

Yes. And you can't have lot many activities then as well, because if 70 are there, I tried Kahoot that day and it was very difficult, 70.. how can I manage 70 on a Kahoot and out of 35, only, only 20 joined Kahoot. So 15, were again, saying, okay, ma'am my mobile is not working, or I have a connectivity issue. If I will do this, then I would not be able to, Oh ma'am, I am joining the class from mobile, then how can I go to Kahoot now? So, so device availability was also a problem. So I think they ensuring that all 70 are learning was difficult with the online classes and engaging the large class in too many online activities was a challenge from where I come from it, from where my context is, because all the students are not having all the devices with them. And, most of the time they are joining classes from mobiles. And if I see, okay, share the screen. So they say, Oh, well, sorry, I cannot share it because, and then show me, where are you? So, so from learner point of view, you get sometimes, it is difficult to engage them fully in the process of learning.

**Speaker 1:**

Right? So, thank you for sharing deep insight about advantages and disadvantages of online teaching. I think it's time to move to the next question where as in the beginning you discussed about the fears of faculty members. So now I would like to know that what are the competencies and skills that are required by faculty members to teach online during the crisis.

**Speaker 2:**

Okay. So number one is, yes, command over a VLE, okay. Having, command over other tools as well. Other, for example, we can have Mentimeter, we can use kahoot. We can be, we now have other mechanisms such as quizzes so that we can engage the learners if it is difficult to engage them in the synchronous mode, then at least they are engaged through asynchronous mode because learner engagement is the key all the time, whether it is face to face or online. So if you are in the class of 70, if you are not able to hear the voices of 50, then it is our responsibility to ensure that they are learning. So we should be having certain strategies that help us, know that they are engaged.

**Speaker 2:**

So, yes, what the tools that I have used so far is a Mentimeter. I have used Kahoot. I have used, the quizzes and, what else I have used... I have used several tools from my own, VLE, which is H five P we also use the feedback form for taking certain evaluations at that, how they are going through, besides that, we also should know, because certain skills also have to be transferred now to the online modality. So we should know how to plan it effectively time wise, so that we don't face to face it was okay, but for online, we should be extra cautious with the planning part because all 70 will not be face to face with you. So if something went wrong, it will be very difficult because students are not seeing you what you are encountering at that time and what is going on. So, your planning should be thorough. Your team, should be on the same page, while, you are in the mode of the online teaching, then definitely using the Microsoft teams or zoom is important. And, using that tool is that these two programs are also one of the competency, which is required by all the faculty members.

**Speaker 1:**

So, need to know how to operate various features of zoom and various tools or educational tools. We can say, like you mentioned a lot of them, and you have even answered my next question, that, how do you have effectively engaged students? Like you said, you used Kahoot and then quizzes and Mentimeter. So I would, again ask you that, how can the faculty effectively engage if there is anything left you can add. So how can faculty effectively engage students in online learning environment? So one of them that test tools using educational tools, any other,

**Speaker 2:**

The slides that we develop, sometimes become wordy. So, from now, what I do that I have turned the shape of my slides, that they are colorful slides, and instead of writing content on it, I used to give them picture or reflective question to think about it and answers. So my every slide has some thought provoking question or an activity. So, it depends how you develop the slides and how you... so let them think, and then there, they will respond. Sometimes I also ask them that, okay, so we are playing now fastest round. And then, I will take the, I will ask the names, but then I will ask the question with the names. So everyone has to be very, very alert. And so everyone becomes alert, and if somebody is not there, then they respond, Oh, ma'am, I'm there. Actually, my mic was muted or not working. So they, the students know that, I can be called anytime during the online class, even, if I'm not seeing them, they should know that faculty will ask a question and I can be asked to answer. So this was one of the strategy which, helped me to make my learners engaged.

**Speaker 1:**

Right.

**Speaker 2:**

And then annotations tool that I use, so that also help us and white board tool also help us to know that how many participants are writing actually, and they are engaged.

**Speaker 1:**

Hmm. So using of educational tools and then using of various features on zoom, like annotation and whiteboard, and asking random questions from the students, make them alert and keep them engaged during the online learning. Right. So let's move to the next question. And it's regarding the students residing in remote areas. So what are your views regarding the use of online teaching and learning for the students residing in the remote areas or different parts of the country? So you have discussed about internet connectivity issue. Any other thing that you would like to talk about?

**Speaker 2:**

This is actually the sore point of when students do not have internet connectivity, how will you connect with them? And some of them are even not having the smart phones. The video calls cannot be done with them, or so, and some of them are not having the access to WhatsApp even. So that actually make learning difficult that this is a sore point that you have right now in ensuring an equally engaging them becomes difficult. So, for that, a yes, it was for us, it was completely a remote experience in which what we did was that even we couldn't send them the material through the .. online.. or VLE was even not accessible to them. So what we did was that we provided them all the content, all the PowerPoints in the USBs and USB was provided to them. And we also shared with them the schedules of everyday starting, but that the students anxious that made the students what we would say learning fatigue. It was like learning fatigue, learning by their own or reading by their own. And if they don't understand anything, they were just, they were just struggling with every concept that how, if they're not understanding how we, to whom to ask and how should we attempt those questions, even, even the assessments exams, we sent them through USBs and they were password protected. And, when they received that they had to study simultaneously. They had to do the assessment and SAQ questions as well. So, so that, that went like this, but whatever, I heard from my students, they said that it was very difficult. We were having, if we don't understand anything, then, then how many things should we ask? Some of them were not having WhatsApp. They were not having access to WhatsApp group as well. So they were not very attentive on the discussion going on through the WhatsApp. So they were sort of, they suffered a lot and they are suffering a lot. So this time what strategy our teaching and learning office took was that HEC announced that only 30% of the students can reside in the hostel and remaining has to leave. So, it was decided. It was a very difficult decision that was taken by our DEAN’s office that year 1 and year 2 will be sent back and only year 3 and year 4 can stay. So only year 1 and year 2 before they were sent back they knew that the material will be inaccessible after they leave. So what facility we give to them that you read everything, you complete everything. You complete all the assessments and then you go. So this was the support, which was given by the teaching learning office so that they, they are not lost and they do not face that learning fatigue, again, reading by themselves and sitting by themselves. And there is no teacher, there is no peer, there is no interaction. So that is a sore point that when the internet connectivity is not there, yes, learning is going to suffer. And the hub and spoke model was created. However, our students shared with us that it was so far from our residents and accessing those hubs was actually, they were, yeah, they are not part of those hubs. And those hubs were the property of other people, other students. So they were not getting... they were like aliens for those places. And they were, they were accessing it as a favor. It was not their right that they can access it, but it was a favor from the community given to them not as a right. So, so they had difficulties accessing it if, and they had to follow their time in the, when they were in the university, they were on their own. And they, they, they used to study at whatever the time they wanted to, but now they had to follow their time strictly because then the school will be closed. They will be sent back, even if their work is not done. So the quality of assignment that I received from those who were, those remote areas, it reflected that what type of difficulty they went through. And, fortunately the semester was pass and fail. If they were graded, they must have been at a very bad disadvantage.

**Speaker 1:**

Hmm. Thank you for sharing. Very important point. And I think you said it right, that this is something that you people have faced during this crisis, especially the students who suffered and who are suffering right now due to what we can say, the lack of facilities. Well, the next thing that I need to ask you, it's regarding the complete shift or the migration to online modality. So what are your views regarding it? Are we prepared or there are still we have some fears or anxiety...over to you?

**Speaker 2:**

I think Most of us are adjusted to it. but, some of those actually in our school, there is a shift actually. So in one of the program was 70, 80% online before COVID. So they were like on their own, they, they were, the students were given a good educational technology courses. Students were quite happy, and they were quite used to off using the online modality. So that was our post RN BScN program. Master students Yes, most of them are accustomed with, but this batch was also from, most of them were from Gilgit. So due to lack of access to internet, their classes were also hindered. So, if they are also saying back then again, master's program also face difficulty of continuing the classes. So this is one. Now we have the four year BScN program, which is a majority of the students are in this program and almost 150 per year. So you can say, we can say almost 600 students with, we have in the four year BSN program. Okay. So now the year one and year two are the one who are the most disadvantaged because they, they, they have not got all the opportunity to, to feel that migration. They are, they have mostly seen the remote teaching and learning. So they came here for, for a month or so, and they were trying to adjust, but they couldn't feel the full scope/full flavor of the online classes and online teaching. So, at that time priority was that quickly let them do their, those objectives, which are required to be done face to face. So they, they quickly completed that, those chapters. And then again, they were sent back due to HECs notification. So year one and year two have not experienced that the faculty members have also not experienced the full scope of online teaching and learning, those who are in year one and two now coming to year three and year four, they are here. And faculty members have experienced, the students have also experienced the full scope of online teaching and learning. And we are implementing every new things that we can do. and I already told you how we are improving day by day. And so, at last, what I internalized through this experience was that when we started, we considered ourselves that we are not prepared and how this will happen. There were lot many apprehensions. When we started and gradually, there was a hope of light that the online assessment, training and then various sessions from QTL net, and then the support from teaching learning office was that our IT experts were always there if any issue encountered, they were there to resolve. And there, it was overwhelming for them as well, but they were not saying no to anybody. That was a big, big blessing. So that helped us to actually dive in the pool of online teaching. We started delivering our classes. We started delivering, we didn't know how it will go, but when we started with then we came to the, okay, the problem is that students are not engaging. Students are not answering. We had strong apprehensions that some of them have really opened, they have really signed in and they are not physically there and they are not listening anything. But one thing that we had ensured was that our summative assessment was made in such a way that we prepared our students earlier in the beginning that look, if you are not going to learn, or if you are not going to take classes, then you are going to lose your many of the things in your summative assessments, you are not going to. So they were adult learners, they understood and then we started and then we started giving them classes. Many a times students also shared that you don't use other modality other than the PowerPoints. So you should also use, so this was one of the feedback, which was shared by students. So we tried, okay. we also should have other modality so that they feel engaged. So then we gone through that experience now where the turning point was that after completing all the theory there was a clinical rotation, and there was a skill rotation where we were going to see our students for the first time. So when I went to them, they said, okay, who are you? And then, when I saw them, I said, who I knew, so where are my students? So, so we were completely new to each other. We had never seen each other, but what was making us recognize was our voices. They were knowing us. And I was knowing them with their voices, not with their faces. That was completely shift. That was a complete shift. And that was sometimes also disheartening that, and passing by, but my students are not greeting. So that was one worry. One of my faculty also told me that what happened, you know yesterday a student knocked my door and when I opened it, I was not wearing the mask because I was alone in my office. And when I opened the office, she said, where is Ms. Sadia? So, can I speak to Ms. Sadia? So she said, I am Ms. Sadia. Oh ma'am, sorry, I couldn't recognize you. You are there. So that was a complete shift for us. It was also not what I would say. It was something which was, we were not expecting that our students were not going to recognize us, but the only connection that we could have was our voices. Then when we took the class face to face the first class, because it was a skill that couldn't be done online after a day or two, when we spent with them, they realized that ma'am, trust us, nothing can take the, nothing can replace the face to face teaching nothing.

**Speaker 1:**

So what I am gathering here is first thing that there are two aspects of it. Like if you talk about the demand of this particular nursing course, it has some theory courses and some clinical courses, right? So for theory, it can be shifted completely to online modality, but when it comes to clinical aspect, there are some restrictions because students need hands-on practice. And, the other thing that you shared is the interaction, which is missing. And, the one example you shared that students are unable to recognize, or it seems like that, the bond has not been created due to online mode, which is possible in traditional learning is what you are trying to say is, am I making sense to it?

**Speaker 2:**

You are right. You're completely right. Because when we write exact connotation, but it doesn't communicate to them that I'm being polite or I'm being humble. And sometimes they take me that, okay, I'm, I'm bit rude with them, but when they, when they came face to face. They gave me a feedback. Ma'am you appeared, you sound so rude on the zoom, but we never knew you are so much humble. And you are so much concerned about students learning. You take students' concern so seriously. So yes, it is a barrier bonding doesn't develop when we are online, especially, especially, and when the, all the learners are not at my level, they are actually kids actually 20, 22 years old adults set group, I deal with, so yes for going to their mentality, the bonding has to be there, which is actually not, not happening in the online modality. Yeah.

**Speaker 1:**

Right. So, so thank you for your response. We are left with few more questions. So, the next one is what have your experiences of supporting the planning of students' assessments and grading in complete online learning environment during pandemic? So you were as a part of the team, like you were supporting the student assessment and grading, right. So what were your experiences?

**Speaker 2:**

So this is our norm that for every assessment there has to be a rubric. So we ensure that, whatever the assessments are proposed, they must have their rubrics. And when the students are evaluated, they are not evaluated on a criteria, which is actually assessing their use of the technology, but it is assessing their conceptual clarity. So we also tried to based in these types of principles, when we were approving the assessments, what we did was that those students who have a complete, full access of online modality, we ensured that our bank questions, which are available in department of educational development, they can be used so that the reliability and credibility of the assessments can be maintained. And we did this the same with the year three and year four, BScN and postRN BScN, so that those who are having full access, complete access with good bandwidth can have at least the reliable assessment. The problem we faced was with year one and year two, to whom we were compelled to provide short question and answers, this increased faculty members' workload for too much checking. But unfortunately those who were in the mood destinations, we couldn't have, we couldn't give them the closed book exam. So the open book exam was one choice that could be given. And we didn't had any other option. So for them, year one and year two we had limited options for giving different sorts of assessments, but for year three year four and postRN BScN we applied all sorts of technology can be any pause, the recording, or shall we, shall we wait for a while? There is somebody at the door

**Speaker 1:**

Okay, I can pause it so you can continue. So you shared about the year one and year two, and then you were talking about more. Yeah.

**Speaker 1:**

Can you repeat the question again?

**Speaker 1:**

I was asking you how you worked for your experiences when you support the planning of students, assessment and grading in complete, online learning environment during pandemic.

**Speaker 2:**

I covered that, that we ensured that the grading is done on the basis of fair rubrics are reliable. They are, they are approved from the curriculum committee prior. We also ensure that all the assessments are approved prior to administration from the curriculum committee. And, no unplanned assessments are administered to the students. We ensured that the questions are available from the bank. They can be used because which are already tested, which are already being tested and they are reliable questions. So this way we ensured that quality assessments are given. One challenge we encountered was that in medical college, the assessment, the close book assessments were conducted with the help of proctoring. Okay. But, so Proctoring is a technology which allows, online invigilation. And if a student is cheating or something, so, the faculty members can actually detect the plagiarism. So, for that we had a limitation because our students didn't have all the devices required or for that particular sort of invigilation. So what we did was that we developed our own guideline for the online examination. We didn't give any closed book examination, but we ensured that those questions are given, which cannot be easily Googled. And, we ensured that students we gave all the examinations which were open book and which doesn't require any invigilation. So, but they were time bound. So for example, if at nine the exam starts, there will be 60 questions. So if nine at nine, if they start, so at 10 30, they should end, and there are 60 questions. They will be long scenario-based questions. And if you go and cheat, so definitely you are going to lose four or five questions, which are an attempted, so students were aware. So what we did was that we administered wisely all the assessments that ensure that our students are able to do it. And if they cheat, they will be losing… they will be conducting one question through cheating, but the time will be so less that they will be missing four or five. So it was designed in such a way. So, due to that, when the results came, we compared those results with the print of the previous, closed book face-to-face examination. And fortunately in at least my course, I'm confident to say that there was no significant difference in this open book and close book examination that we conducted. So, so we were able to identify bottom scorers and in all the other courses of my year that I teach in year three. So others were also saying that we also encountered that those who, those students who were found to be slow learners in other courses, they were also found to be having a bottom scores in other different courses. So, our guidelines were developed in such a way that the slow learners or the bottom scorers were identified and high achievers were identified as well.

**Speaker 1:**

Right. So as you were discussing about the challenges that you faced while using that particular, software or application, I would like you to talk about some more challenges that you faced in implementing online teaching. So not only assessment, if you talk about online teaching or while supporting the students and faculty members, were there any challenges that you faced?

**Speaker 2:**

Yes. So students competency in using the different tools of the computer. So one was that their availability of the devices, this was also a challenge and gradually our faculty members become accustomed with all the technology and the skills required gradually. They are learning, and I would send, at least none of them are there who are completely dependent. All of them has tried their best to meet, uh, the requirements for online teaching and online teaching also helped us to make all everything electronic, actually. So now everything is electronic, everything is in writing. So that also, uh, was a benefit, uh, for online teaching and learning. Uh, so, uh, other challenges, I think the major that I felt, uh, I,, faced was from student point of view. And one thing I would like to add here was that, uh, yes. Oh, I also told you about. It was difficult to know that how many of them are actually attentive and how many of them have yeah. That was also a problem.

**Speaker 1:**

A large class size was a challenge.

**Speaker 2:**

Yes. Yes. Large class size was a challenge. Marking attendance was a challenge at times. Microsoft teams was a challenge. Yeah. Yes, yes. So Microsoft teams itself was a challenge because everyone cannot have, uh, every week for every class, we cannot have zoom. So for, for, uh, in our, in our, uh, school, what was that, that, uh, all the undergraduate classes used to be conducted on Microsoft teams. So it is a free software, but, it has several issues that you cannot use the full fully scope of all the tools you cannot, because it is slow. It, it actually make your system slow and it hangs many of the times, or you cannot see the full participant list, uh, because, because your students are coming as a guest that you cannot see full participant list. So marking the attendance was, was a challenge on my Microsoft teams.

**Speaker 1:**

Okay

**Speaker 2:**

yes, Microsoft teams was difficult for managing the large classes for some, some of the classes where I thought that Oh my God, this cannot be done on Microsoft teams. Then we used to ask for a zoom link, which our program coordinator used to provide us, but not on the short notice.

**Speaker 1:**

So what are those things that cannot be done on Microsoft Teams.

**Speaker 2:**

for example, just, I shared with you that we couldn't see the full participant list, so some of the, because our students are not AKU employees. So all the guests, you cannot see all the guests, then everyone has the host right. It was the problem. Yeah. So everyone, when everybody has a host, right. So if I don't want a student to admit, so other can admit, then in the chat box, sometimes all of the students do not have access to the chat box. Okay. This was a challenge then, some of the students also said that we do not have access to raise hand option because we are guests. So this was a challenge, right. Then, one more thing was, let me think about it, which was a very important issue that I encountered with teams. Pause..... Yes. Breakout, breakout group. During teaching, you cannot make a breakout group. If you want to make a breakout group, then you have to inform the coordinator prior to the session, at least two to three... These are my groups. So now divide them and make separate teams for them. And then in zoom, it is easier for you to see all the breakout groups, and then you can switch going to students to facilitate them what they are doing. Right. But for teams, it is not possible.

**Speaker 1:**

Okay. Yeah. Right. Thank you for sharing a lot of challenges. Uh, I would like you to, uh, tell me about recommendations. So what are your recommendations to ensure sustainable remote teaching and learning in the future? So anything regarding resources or anything, which was, uh, lacking. So any recommendations

**Speaker 2:**

Yeah. Something has to be done in a longer run. something has to be done for those who are disadvantaged.

**Speaker 1:**

Yeah.

**Speaker 2:**

Yes. Because they are disadvantaged. They just need support. They are having every sort of, I mean, in terms of poverty.. So yes, they are not having devices that yes, they are not getting every sort of support they need. So something has to be done for them because ultimately they will be the sustainable members of the workforce. Those who will be powerful enough, they will go away leaving the country and the profession behind anywhere. But, those who choose nursing, they are actually willing to change the fate of their families. And they are mostly from disadvantaged background. And, the university should identify them and, and do something for them that at least to sustain connectivity for them to sustain access for them, so that their learning is not hindered. One thing is that and continuous faculty development for online teaching and learning, and, then also forum, where faculty members can share their challenges, this has to be there so that, proper recommendations can be brought into, the refresher for online modality is also required. Yes. , and one thing that yes, I understand that for year one and year two we had to test all the modalities that we could, we could ensure their learning is not hindered and they are progressing well, but I still feel that, , they're teaching learning, and when they will come to year three because their clinical has not been done yet for year one and year two, year three, and year four. We have ensured that when they are entering the workforce, so they are having ample clinical exposure before they are moving to year four. And before they are year four, before they are going to the workforce, but for year one and year two, and just having an apprehension that when the year two will be coming to your three, they will be completely blank. So the school has to think that, how we are going to manage those lost clinical hours because nursing education cannot be completely online. So that is also important. And, when we are saying that yes the opportunity for virtual reality simulation need to be explored more so that our students are more engaged and, not only the virtual reality simulation, but how faculty members can transform their previously developed classes into online modality not compromising the principle of engagement.. Learner engagement. Yes.

**Speaker 1:**

Okay. So, uh, thank you for a lot of recommendations that you have shared, uh, last two questions. The second last is that, how do you see university support or role in executing, remote teaching and learning program?

**Speaker 2:**

I see the role of QTL net has been very important in doing that in sort of capacity building of faculty members. And, mostly it is nomination based or wish based, that sometimes if I want to go, if I have time to attend that tech lounge, if I had time to attend boot camp, then only I will go. But certain workshops that QTL net can do, compulsory entity wise, for example, one day QTL net is coming to SONAM. And this session is mandatory for all. Everyone has to do it. And one day it is going to DED one day It is going to medical college. So this sort of Mandatory capacity building has to bepart and parcel. one thing that university can do and university has also ensured that those faculty members who were not having access to proper devices, they are supporting those, but there is no announcement, or there is no email open that those who are facing difficulty, so they can talk to this person, if somebody do not have the device, or if they are working from home and they do not have the proper device to work from home, then I don't think so that there is any particular announcement or email from the leadership that if you are facing difficulty, you can have, you can contact this person and you can get your devices or so, so I think yes, this is the issue. So protection is important. And one more thing is that, we have been told that 50% of the faculty can work from home. So remaining 50 can come, but sometimes if I have to take a large class, I cannot risk of connecting from home, because internet connectivity is uncertain. And if I get disconnected, my 70 students will suffer. So, if I decide to come to office and if at the same time, my roommate is also having the class, then it was used to be a problem because, when I used to speak, she also used to speak and everything used to get chaos. So yes, space is a problem. This is also a problem which a university can be revising the sort of layouts existing layouts. We need to revise that. For example, if this class is going on. So previously we used to give venues for classes, but now there is no infrastructure. So in the online modality, if this class is going on, so all the venues are vacant And how should I go for the booking? There is no support from the program office that, okay, this class and this venue is booked for you, so the students will be online, but you have to go in the class and you have to conduct the class over there. So, this was not happened. I should sit in my office and I start taking the class no matter whatever the background is, whatever my colleague is speaking. So that infrastructure needs to be developed.

**Speaker 1:**

Right. Uh, so this, I have noted down in the recommendations as well. Um, so moving to the last question that, uh, how SONAM, uh, can be a trendsetter or a role model in introducing remote learning program to meet country's nurses, demand and healthcare system.

**Speaker 2:**

I think capacity building, if we are doing so much, then, we should publish this and write these papers and not only publication but also we should impart this knowledge to the other universities and we should develop their capacity and SONAM has several grants which can be used to do so, we should teach, we should train other institutions across Pakistan, that how we have gone through the online migration and how we have sustained the teaching learning. So the similar workshops can be conducted by our faculty members. So we can show the institutions across Pakistan that how we have reached out to them, to our audiences and to our learners. So one thing can be that we should follow the best practices all the time before applying any, any new concept in our practices. So, that curriculum committee ensures, and from curriculum committee our processes are quite transparent that, once the faculty writes the proposal, they come to curriculum committee and then from curriculum committee, it goes to RWG. And from RWG, it went to HEC. So our processes were quite, Stringent and streamlined prior to whatever we applied and implemented. So that can be sustained in future as well.

**Speaker 1:**

Right. So following the best practices in showing what you are doing through the publications, or sharing it with other departments, I think this can, uh, help SONAM to be a trendsetter and a role model,

**Speaker 2:**

Not only the departments, but other, uh, nursing universities and other competitors.

**Speaker 1:**

Right. Um, any other thing that you would like to share?

**Speaker 2:**

I think I have shared everything.

**Speaker 1:**

So, yes, your ideas and answers are valuable to us. And thank you for taking out time. Uh, last thing that I need to tell you is that still, if you feel like, uh, sharing anything, or if you missed out, or if you think that, uh, it's right now, it's not a comfortable place to share in if you want to write. So there is an option of writing a reflective log, and you can email that on the same address that from where you received the zoom link. So that, that is another sharing.

**Speaker 2:**

If I feel that something very important I have missed out.

**Speaker 1:**

Okay. Right. so I think that's it. And, thanks once again for taking out time. Is there anything that you want to clarify or you want to ask otherwise we can end the meeting?

**Speaker 2:**

Okay. So for postRN BScN, fortunately whatever we designed was implemented, in the same way, because our students had most of them, almost all of them had access to the internet and they were available to take classes online and, but also available to perform the time bound, synchronous examination as well. So for the challenges that I talked about earlier regarding limited access Post RN program was exempted from all these challenges. but yes, for postRN program, what we encountered was what I, as a faculty encountered was that, since the students are, coming and most of them are part-timers or they are doing their studies with their job. So their engagement used to be there much more when they were face to face. But when the modality has changed to online, we faced that, that they were slow slight, their engagement, engaging them was a problem initially when we were doing the online migration gradually, but, with them, it is a problem always that yes, because they are balancing two things. So their performance is not the same as we see other programs or other students doing who are the full-timers. So it is a challenge for postRN students always. but from students' point of view, it was at the student's level, students found it doing it whenever it is feasible for them, they didn't required to travel to attend their classes. So, so yes, it went into their favor. but, I being a faculty felt that maybe face to face learning would have benefited them more.

**Speaker 1:**

Right. I think, uh, this is, uh, specifically to PRN, group, so that will also help in enriching the data. So, uh, thank you, Ms. for your time and, uh, uh, talking about all what you felt and what you experienced. So that's it from my side. Is there anything that you would like to share further? You can write a reflective log.

**Speaker 2:**

Sure

**Speaker 1:**

Thank you once again. Take care. Allah Hafiz

**Speaker 2:**

Take care Allah Hafiz
