# Supplementary material for: Beyond the classroom walls: Stakeholder experiences with remote instruction in Post RN baccalaureate nursing program during the COVID-19 pandemic: A qualitative inquiry
Source: PLoS One. 2024 Apr 4;19(4):e0300007. doi: 10.1371/journal.pone.0300007 (PMC10994296; doi:10.1371/journal.pone.0300007)
Supplement: S5 File — (DOCX) [file pone.0300007.s005.docx]

**Speaker 1: Ms 1**

**Speaker 2: Mr 2**

**Date: January 22, 2021**

Speaker 1:

Thank you so much, Sir . So, is there anything that you need to ask me otherwise I will begin with my questions?

Speaker 2:

Yeah. Just go ahead. No problem.

Speaker 1:

Okay. Okay. So the first thing that I need to ask you is what are your views about online teaching learning?

Speaker 2:

I would say first that it's the way of the future, is the way of education. The online component, especially in teaching and learning. It's something that has been ignored for quite some time. At the time we had the pandemic and it was .. People had to just go online. Some went into online to be able to survive and remain relevant others were like this is the way that we really wish to teach. And it's like they want to know more, especially in terms of how teaching and learning should be done online. So what I want to say is that online teaching and online learning is the way of education and it is something that we really need to embrace. Thank you.

Speaker 1:

Right. So, what I gathered is that this Covid has become a window for opportunity for us in order to experience this online teaching and learning, which was neglected before. Right. So let's move to the next question. And it's regarding your experiences of supporting the migration of existing curriculum to remote teaching and learning during the pandemic and how can faculty and students be better supported in this transition.

Speaker 1:

So shall I repeat the question?

Speaker 2:

Yes, please do repeat the question.

Speaker 1:

So what are your experiences of supporting the migration of existing curriculum to remote teaching and learning during the pandemic?

Speaker 2:

Okay. So maybe just, I start with that and then when it, whatever, whatever I miss out, maybe you can just be able to remind me in terms of the experience, especially for the migration of the curriculum. I think it was met with quite a number of challenges depending on where faculty were in terms of use of technology. I know that it was quite difficult because people wanted to do what they've been doing in face to face to online, somewhat running a three hour session, maybe on face to face and they wanted to do the same. And they found that by the end of it all, it's like, you don't have all the students, the students have faced with a number of challenges and it is like this class is not as smooth as you really expected. That's why in supporting faculty, what we did is that we asked faculty to be able to look at what they're going to teach plan very careful so that there are some things that they should be able to cover asynchronously and others can be covered synchronously. So we really advocated for those two months in terms of online learning, that is the by chronous kind of blending, that is a synchronous and asynchronous, where now we worked with faculty without those ones, we would even meet every week to be able to see to it that whatever they want to use asynchronously that is on the VLE Moodle was done, especially in terms of how they were able to package or their content on the VLE class, the activities that were associated with it. The VLE that is used in Aku that is the Moodle. So we had someone who had already started teaching, using the VLE during face to face sessions before COVID. And those ones had quite some easy time, a in terms of having their materials uploaded and making a few adjustments here and there, but the ones who had not, then they had to struggle to be able to fast learn how to use the virtual learning environment, the VLE before now, you are now able to have them packaged or have the information, the content package for teaching and learning. So it was a bit of a getting people from where they are, those who already had an experience with VLE and those ones who had not. And we worked with the, both the groups. We had quite a number of sessions across the schools including across East Africa, across Pakistan. So that was quite a bit heavy for us, but we work tirelessly to ensure that we are supporting them across, because we had our support required across SONAM across IDE across GSMC. So that was quite a bit heavy for us, but I think we were able to manage since we've been working with faculty for quite some time. So that is what I can be able to say in terms of the curriculum. It was met with quite a number of challenges as I could be able to point out challenges in terms of connectivity, even for faculty, because you could be handling them with a session in terms of one-on-one consultation. and it is like by the end of it, or they are, some of them are disconnected during the sessions, but what we learned is that it's the whole idea of being patient.

Speaker 2:

And you have to be patient, especially with the technology, be patient with them so that you are able to bring them to speed. By the end of the day, I think it was a, it was really, really appreciated. So some of the things that we took them through act points, we would have like all group discussion or whole group sessions where it's like an entity. We are able to meet all of them and be able to reflect on their experiences in terms of teaching what challenges and how we can even support them better. That is a whole group. At one point, we even had small group sessions. We have maybe faculty-wise sharing sessions or lessons units could now be able to come on board. And we are able to check them through certain areas that they find difficult in, and also be able to introduce to them some of the things, the new pedagogies that they can be able to use even during this particular pandemic. So we had this small group sessions. We also now apart from this small group sessions, had what we call the one-on-one consultation clinics. And those of us who are able to meet with a particular faculty, one on one, and you're able to address some of the issues that they are facing, especially in terms of the technical needs, the pedagogical aspects, some would even come and tell me next week, I'm teaching this particular lesson. This is how I'm going to present my session and the comments that you have be able to give me an input would help me. So those are the things that we would really take them through. And it was quite a very kind of interactive session. So depending on the entity and whatever people were interested in, at one point we name and have had what's, now we could call reflective sessions with ease some of the entities. So that to be able to just also be able to look at, if they are able to teach the whole semester, what are some of the things or lessons that we can be able to learn from the experiences, and then how do we support them better in the next semester? So that is a, those are some of the things that we can be able to share, especially in terms of moving that curricula to online. So it wasn't, it wasn't like it's too, just to be like uniform one, fit for one size fit for all. But it was that you really needed to understand the needs of the faculty so that they are able to support the students better. Yes. Thank you.

Speaker 1:

All right. So, what I gathered is you, people planned group sessions, you people planned reflective sessions, one-on-one consultation and need based sessions that were effective for the teachers. Right? So, I just want to ask you, we don't know how long this pandemic will take and might be transition will be for one more year or whatsoever, six more months. So is there anything in pipeline to better support this transition or you will be carry on with the same practices that you were doing.

Speaker 2:

Okay. The same practices will still continue. We always in our entity QTL advocate for one-on-one sessions. So if faculty have some needs, we expect them to write an email and then we set up a session for them. We also are open to all group sessions. If one entity had faced that we need to reflect with their teams, we are also open to that. We also advocate for small groups. So those sessions will still be able to push them in terms of support, whether the pandemic is there or not. Because a teacher, you are not really the owner of it all, you are not an expert in everything. And that's why we would also want to take those programs for one, especially in supporting the curriculum or offering curriculum support to our faculty, remember they are not like they're trained instructors or teachers. So the support is supposed to be like an ongoing kind of a thing. And remember, curriculum is now not like a fixed kind of an entity. There are new things that I imagined out of it. So that's why those programs are supposed to really continue. But alongside that, we now expect them because as a team BDL and QTL, we are putting programs in place that should be able to support faculty throughout the year. So we are bounding workshops. Those ones will also be able to out of those particular workshops, there are new things that will emerge, faculty will need some more support in the same, so that support will still continue even after the pandemic.

Speaker 1:

All right. So, anything regarding students point of view, like, to help students during this time, or any workshop that planned for students, anything like that, or this is all for the faculty members?

Speaker 2:

No, what we do is that when we are planning for these sessions, we always have the student in mind because now we are not talking, not talking of justice, student centered, we are talking of learning centered. So when it is learning centered, then the learner is like at the center of everything. So we are doing all these, have the learner in mind. And that's why we now have we're catching. We have now as standardized kind of a program when it comes to student’s orientation and not the general orientation for the entity, but student’s orientation when it comes to the use of the virtual learning environment. So we take them through a 90 minute session to be able to understand their online requirements. So, so far, like from the time they year began, we've dealt with some two entities that is the ISMC students. We had an orientation with them. We've also had the orientation with the medical college students in Pakistan. Yeah. So you can see, we have a program, a standardized program that has been approved by even the registrars group, the registrars group, in terms of students orientation, it was approved. And that is now what we are using each takes 90 minutes, as I've already said. And it goes through some of those things as it is, as opposed to really expect, we will do some evaluations to be able to see that we understand them because they bring on boards like their fears. They share with us the fears also in the introductory part, they also share with us some of the hopes and how they expect even to learn at AKU. So that is what we have. We have a very, like a vibrant, now I can call it to use the word vibrant kind of orientation program that is now been able to be shared by all the entity heads. And it is now being used across.

Speaker 1:

So this is in planning. Okay. Thank you for sharing this point. Let's move to the next question. So in your view, what are the advantages of online teaching?

Speaker 2:

The advantages of online teaching. There's a lot in literature about online teaching, but what I can say is that when it comes to online teaching, it's like we're now in the 21st century, just looking for just-in-time learning. You had of that just in time teaching or just in time learning. Yes. So if we really want to push just in time learning, then online learning is the way to go right now, I'm talking to you from the comfort of my chair in the house. And we are able to like go through this particular session, which to me, I call it a learning session because I'm learning from you. And you're learning from me. And we are saying that online teaching or learning is able to support the 24 hour kind of support we require for our students and faculty. And also seven days in a week so we have no excuse. We don't have an excuse of a missed lesson. We don't have an excuse that I was traveling. I could not teach. We don't have an excuse that I was sick. I could not teach because you will still teach from your sick bed. In terms of your lesson, you can still be able to facilitate that as long as you have some little strength to be able to do that. So when it comes to online learning, it comes to teaching and learning. It comes with all the advantages. Although it's been looked at it traditional from the traditional perspective, very negatively, and it has been marked in many, many aspects, based on the challenges that people have on some of the investment that has to be done for online to take place. Like for example, investing in the infrastructure in terms of the laptops, the connectivity students might not be very comfortable loading their laptops with bandwidth, unless maybe they're provided with data bundles, but institutions providing data bundles that is not sustainable as what some of our entities have done. It will be a short-lived or short-term, but it is not a long time measure. So the question would be how would we as a nation or countries be able to invest a lot in terms of internet so that we able to also match the developing countries, in terms of the connectivity, so that when it comes to coordinate learning or online learning, it is just like the way we've treated or looked at the traditional, or the face to face kind of teaching and learning. So I would say that, even after the pandemic or a post COVID, what we will have is that we will have what we call it, blended kind of learning. And that would be the right way to go, especially when it comes to addressing quality. I look at the way things, people have been scrutinized in their classes in terms of what are you putting up? What are you going? What is going on? Like unlike what has happened in the face to face sessions, if somebody walks to the class, they can even go to class, just give stories to students. And it is like the three hour session has gone. But when it comes to online, Sir is asking you, what have you put up? Where is your course outline? Is your course outline, being able to meet, or is it in line with the learning outcomes that you've been able to come up with? How are you going to teach? What strategies are you going to use? And like what used to be there in terms of face to face. So that's why I'm saying I'm not being biased, but having had an experience of both face to face teaching, I have taught face to face for over like 15 years. having also taught online for another like 10 years, I think I can be able to confidently say if we are really to address the issue of quality teaching and learning, if we are not being purely online, we have to really do blended learning where we are blending between face-to-face and the online sessions. So that is what I can be able to say. I have a lot to be able to share on that, but maybe because of time, I stop down that question.

Speaker 1:

No, you also answered a lot of other questions as well. The advantages that I have got from you is that it's not bound to place. It's not bound to time. And a person who wants to learn can learn from anywhere and wants to teach he, or she can do it from anywhere. So, right. Sir , lets move to disadvantages other than the internet connectivity issue and power failure and all that you haven't mentioned, what are the disadvantages of online teaching and pandemic situation and how can these challenges be mitigated?

Speaker 2:

And so in terms of the disadvantages, now that we are coming from third world countries, I'm referring to Pakistan, I'm referring to Kenya. I am referring to Uganda Tanzania now that we are getting, or we are coming from third-world countries, can be sure that, we can talk of as many disadvantages or challenges as possible, when it comes to online. But as we said, when it comes to online we are tracking of any time, any place anywhere, and, you know, when you go anywhere learning and anytime learning, it has to be made with quite a number of challenges, because sometimes there are those distractions you are talking about people missing that kind of physical presence apart from the social presence that people would really want to have. Like now, I really wanted to have been seeing you and seeing how your lips are moving, how your eyes are connecting with mine so that I am able to see, are you understanding what I'm saying or not? So that's kind of a component. And the advantage we have in Aku is that we have like small classes, but in other universities, that big classes, and sometimes just having that one-on-one interaction is really missing the member. When it comes to teaching, we have what we call relationships or interactions. One is the students interacting with content and other interaction is students interacting with students that is like kind of peer interaction that has to be seen in the classroom. The other interaction is teacher-student interaction. That has to be there. When, as teachers are preparing lessons, you say in your lesson plan, by the end of the session, the learner will be able to not learners. So you are not going to that class, even if it's that class of 82, teach 80 you are going to teach one particular student in that class. So the student has to feel that I am relating to the teacher, but now are looking at what is done. It's like you only see people and images and faces on this screen, and it is like they're going through in some kind of interaction. So you find that some of those interactions are compromised. One of the interactions that is compromised is the student-student interaction. So we now don't give them, even if we do group work, even if we send them in breakout sessions, the interaction is not as we really expect in terms of it being a hundred percent. The one on one, each student is supposed to feel that I'm connected to the teacher and the teacher may be addressing to all group of students. That, to me, that interaction is also compromised in terms of content. So those kinds of interactions are compromised in a way, and as are result, then it is like, Oh, there's a lot. Or those kinds of disadvantages we talk of. So it's like they keep students miss each other. It's like, we can't even now go for, even if there is the forum or the platform, it is not reallyreally active forum. Yeah. We just trying to like, copy, copy what is in there face to face and putting it online. So, it's accompanied by quite a number of disadvantages. So, and then if I mention about the challenges, the challenges are many, some students have funny gadgets, very old kind of computers, or some lecturers even have some outdated kind of computers. And you see, there are some letters and software that need to be used. So you find that you don't have access even to some of those softwares that you really need to have. So it becomes a bit of a challenge. Rita, are you there? Yeah.

Speaker 1:

Yes. There was some problem with my headphones, but I was listening to you. So what I've gathered is that due to lack of students engagement, lack of interaction, and then what I, what I thought when you were saying about that when we write it,

Speaker 2:

I haven't said lack of, I've said inadequate kind of interaction. It's compromised interaction is compromised.

Speaker 1:

Yes. Okay. So, thank you for sharing about the disadvantages and the challenges. When we talk about these challenges. Are there any plans to improvise those or to overcome these challenges, especially from a teacher's side or from the student side, because, when we say that students are there behind the screen, we don't know what is going on and whether the student is achieving those objectives, that learning outcome is achieved or not.

Speaker 2:

Okay. Yeah. So the most important thing as I already mentioned is that we will have to continue with a teacher support or faculty support programs, because by the end of the day, there has to be the ownership of, in terms of the classes, the ownership of learning, where are we related to the faculty to really be in charge in terms of how they would really want to teach number one. And then in terms of the ownership in the, on the side of the students will expect that faculty are able to devise activities so that we have, when students are to interact, let it be a session that is interactive. So that's why we say, when it comes to the zoom sessions, the synchronous sessions, there has to be a lot of interaction. So group work is supposed to be done. People have supposed to also realize that it's not just going to class to cover content. It is going there to be able to facilitate learning. So how do we facilitate learning? And we have to really know that you are a caring faculty or a caring teacher for that particular matter. So it is just important that we have the ownnership that is taken care of on both sides in terms of faculty in terms of the students so that you are not worried that they're not doing nothing, what have you designed for them to do? Yeah. If you haven't designed, you want them to just be listening to you. That is something that Is quite boring when it comes to like, they only listen to you as the instructor, what are they bringing? What experiences are they bringing to class? That is what students want to know, because they, they are not stuck with us as they are not empty slate. They want to bring something. What, what work have you designed? Teacher support is very, very important. It will help them to be able to design instruction in a way that everybody is coming to that particular class. As a participant. I sometimes love when the faculty complain that students are not concentrating in my class. When they say that I know the faculty is the problem. The teacher is the problem, not even the students. So that is something that we really wish to work with the lecturees, the teachers, the faculty, so that they are able to know how do I design my instruction so that it is able to bring out learning and the kind of interaction that you would really still match what is done in terms of face to face. So that is something I could be able to mention, go ahead.

Speaker 1:

So it's a teacher's job to grab students' attention in the class and to help them engage in the class, right?

Speaker 2:

Yes. That's why designing of instruction is something that you cannot say that, you know, you don't know. So it's a learning kind of some experience that you need to develop with time. Even some of us who have been teaching for some time each and every session we keep planning, we keep asking ourselves questions. How can I do it differently? How will my students be able to perceive these, what we learn, where they learn out of this particular session? So those are some of the question you have to really ask yourself. But if you want to teach the way you taught yesterday, then students will not be in your session the next week they will go away. Yes.

Speaker 1:

Okay. So, because we are talking about faculty members, it's a very right question at this point that what are the competencies and skills required to teach online during the crisis?

Speaker 2:

Okay. As I said, the way of education is really changing and we can't teach the way we were taught. We'll be fooling ourselves. So we have to do it differently. So they are what we call like a 21st century kind of skills and competencies that teachers need to have as skill. Like intrepreneurship, that is something that teachers should be able to, because our students are not just coming to a class, they want to be marketed. So how are we helping them in terms of developing those skills? If a lecturer, or a teacher is not an entrepreneur in a way, then by the end of the students, the students will have the Paper and they will not have those necessary skills. The other skill is the skill of collaboration. A lecturer should be able to be equipped with the collaborative skills. How do you engage the students? How do you ensure that they collaborate? And that will address the aspects of interaction that I mentioned in terms of how they're able to use a technology in terms of tech savvy, they're supposed to keep improving themselves in terms of technology skills. So that is something that they really need to have. And the other thing is that a skill is that they need to really be able to communicate. So you don't ask students that Students are questioned that they'll go online and Google and give you an answer from Google at then Now you penalize them that they have plagiarized. Your question was wrong. So if you ask a wrong question, or if you ask it strongly, then you expect to get those wrong answers that we get. We would want the students to have innovative skills. So the other aspect is innovation. If not, you don't have to use innovation. You can call on top of creativity. Very few of our faculty could be like creative. That's why I say, don't teach the way you taught yesterday. It has to be different. What innovations are you bringing to class? As a faculty, that's innovation, students will be able to tap into it. And by the end of the day, you will be able to develop those innovative tasks that we are talking about. So those are some of the skills and competencies that would really be required, especially when it comes to people taking technology and teaching and learning for it.

Speaker 1:

So could you suggest some of the ways that can help faculty effectively engage students in the online learning environment? Some of the examples that you did in your sessions to engage the faculty members. So how can they help faculty to engage students?

Speaker 2:

Okay. For maybe Just in short. What I would say is that for every session that our faculty is supposed to do, it has to be accompanied with a lot of planning. So when it comes to planning that tools, that we've given faculty for planning, for example, a tool that we use for lesson planning, like BOPPPS, where you need to have a bridging, you need to do an outcome for your session. You need to say, to do pre-assessment, you need to set up participatory activities for your session. You need to do a post assessment. You need to give us somebody. Yeah. So that's just an acronym, but we are using, like, for example, it's called BOPPPS. And we use that, especially in terms of lesson planning. So that for every session, you look at it and reflect on it through that way. So whether it's online or face to face, the same way of lesson planning has to be done, but you see faculty at higher education would always say that lesson planning is for those ones that ECD at secondary or high school, for that matter for us, we know what we are doing. So you don't do it, but I'm happy across some entities in Aku. They use what we call lesson guides. And those lesson guides have incorporated some of the tools that I've mentioned, especially like BOPPPS planner in their lesson. That is how they can, and then I would also advise, let's say, faculty should they continue to engage in some of the trainings that we offer the workshops that will help them to as an eye-opener, especially in terms of the new techniques that they really need to use.

Speaker 1:

Yeah. Okay. So I think that's too much about how to engage students and how to grab students' attention. Now it's time to talk about the students residing in remote areas or different parts of the country. So what are your views regarding use of online teaching and learning for the students residing in a remote areas?

Speaker 2:

So in terms of remote areas, I know there are some places, especially in Pakistan. Wow. Yeah. Very, very remote. But what we did was that we had to bring in some different, other ways in which they could be able to access some of their content, for example, adding the materials and resources on our flash or flash discs, or USB drives for that particular matter. So that was something that was used. Sometimes you record the lecture and you're able to share with them the recording so that they are able to watch at their own time. But all those ways that we do to be able to demonstrate address remote teaching and learning and compromises on some of the things that I've already mentioned in terms of interactivity engagement, and even when it comes to learning, but you see, we really have to like go over some of those particular challenges. And that's why we had to put in those measures. And sometimes the students have to come on campus or to get to a center once in a while to be able to access good internet connectivity. So it's all like trickled down in terms of how flexible are their students, could they be able to get out and get to those particular test to be able to access some of the things, but, lecturers or faculty here are supposed to already be caring so that they could be also be able to take care of them because we have what we call special groups. And when it comes to special groups, in terms of teaching and learning, it's like, even if you are moving faster, that teacher, you have to move slower because of this special groups. So those are some of the considerations we had to put like a, you need to put in place to be able to get to that a remote planner, just ensuring that if there is a communication, how do you communicate with that learner? And that's why I said, you will have to handle all the students individually so that you are able to address some of their needs because that is what learning is all about. So what I can be able to say in terms of those residing in the remote areas. So at the use of the virtual learning environment, was also very important. Whenever they have some internet, they could also be able to go to the VLE and be able to access some of those recordings of their own. and that is it. I remember at one point in Pakistan, it's like, somebody was sent route to be able to deliver some of those USBs to some students in the remote areas. So it really called for a lot of sacrifice. And that is what it takes to be able to support that remote learner. Yeah.

Speaker 1:

Okay. Alright. So, What are your views regarding complete shift or migration to online modality, keeping everything in the mind that about the challenges and all. What do you still think about it? If we completely shift to online modality,

Speaker 2:

If we completely shift to online modality as an entity or Aku, I think we will be cheating ourselves. Is that too heavy? Yes. At the level at which we are, is that, even if you were to do a complete online or shift completely, it has to be gradual. It has to be after quite a while of time because our students have been faced with a number of challenges and those challenges will still persist for quite some time. And if we shift completely, then we will still just be doing it to justify our online. But in real sense, we will have compromised on teaching and learning for those students. So the appropriate way for Aku would be kind of a blended approach for now where we are mixing the online and the face to face, because shift in that is assuming that the students will be able to access the library without any challenges. They should be able to access some of the resources in terms of the labs and the, the labs that they need completely without coming to campus. So it's like you will have empower the Learner with some of those, which means we will have like a replica of Aku where they are, which is not something that, we'll take maybe 10 years. For me, we can only maybe do some short courses. The short courses are the ones we can now say, maybe we just have them fully online, but for the courses that would have full semester and we embraced the blend, the blend will be quite okay. And this will be the way like we should be able to go for.

Speaker 1:

Alright. Alright. So now we are moving to the next question and it's regarding students' assessments and grading. So what are your experiences of supporting the planning of students' assessments and grading and complete online learning environment during the pandemic?

Speaker 2:

Okay. In terms of planning for this particular assessments. Yes, it's one of the things that we did during the training is that we would work with faculty to ensure that to reflect their curriculum. They should be able to see what are some of the assessments that they will continue with. What are some of the assessments that they'll have to drop? What are some of the assessments that they need to modify? So that is in terms of the training. That is what we really asked them. And they had to design an assessment plan when it comes to their online session. In that assessment, we had now things in terms of how are they going to assess this particular student? Remember some of the assessments are supposed to be graded others are not graded because we are talking of assessment as learning, not just assessment for, or of learning. So if we are talking about assessment as learning, so when it comes to that, the guiding principle would be, what are some of the things that will be graded? They had to make that choice as they develop the assessment. The assessment plan, the assessment was to be that, say, faculty were also to look at their entity policies to see, could they be able to shift, because sometimes we give less attention to formative assessments that is the ongoing assessment in the class, and we give more weight to the subjective. So that, is there a way that they can be able to give weight to their formative assessments and maybe give less weight to the summative assessments, which is that they will only prepare, do our end of term exam. And in some of the scenarios, we will have that, like for example, it kills examinations. It's like, you really need to be in the lab. And some of those ones who are be done with the students are on campus, but shifts who are organized in some of the entities, to be able to have students being assessed in that particular area. But what I could say is that we had a lot of reviews and reflections when it came to the issue of assessment, so that we use it to be able to promote learning rather than using it, to be able to only gauge how the students have been able to really perform. But how are you fitting it in the outline so that it is able to help you achieve the desired learning outcomes.

Speaker 1:

So, just to ask another question that while having the online assessment last semester, did you people or the faculty members have communicated any challenges or anything regarding online assessments?

Speaker 2:

Yeah. When it comes to the online assessment is the same way when it comes to teaching because the same challenges still remain in terms of connectivity, because in some of the assessments, the students who are required to have like two gadgets, some of them could not afford one, to only like what be the screen you're using the other one to ensure that we are also able to monitor what you are doing, you are in your individual location. So for the ability of those kinds of ideas, it was not something that was easy for some of them. So it's like they really had to struggle to ensure that by the time they are doing the assessments, I think that those kinds of, or the examinations, and it's like how we need to, you need to have those kinds of connectivity issues. The bandwidth was also quite a big challenge when it comes to the students and us doing assessment, you'll find that during the assessment. Some of them are disconnected. So it becomes a big, big challenge, also they are also applicable to what happens when it comes to teaching. And then the way I say that we are not just assessing to gauge their performance. We are using assessments to be able to facilitate planning.

Speaker 1:

So, right. So we are left with a few more questions. So now it's time to talk about the recommendations from your side. So what are your recommendations to ensure sustainable remote teaching and learning in the future? I know you have discussed some of the recommendations in the beginning that teachers need to plan in a different faculty needs to perform better when they come up with questions that we are unable to grab students' attention or engagement. Is there any other recommendation regarding training session or anything that you would like to suggest?

Speaker 2:

Yes, my recommendation would be moving forward. There has to be continued support for faculty, especially in this, you know, that those things will trickle down to the learners. Then. the continued support in terms of what they are doing, technical skills, pedagogical kind of skills, those ones. And then we need to continue with their professional development programs, like for example, or what we have in the QTL and those rethinking teaching workshops, the teaching, learning enhancement workshops, those workshops are supposed to continue because they help you, especially in terms of like planning for your lesson, planning for your cause, revising your learning outcomes. Because, we realized even when we were doing the training, is that some of the learning outcomes were outdated, so you would even challenge the faculty that can you revise your learning outcomes. So those professional development sessions are very, very key. And then the other thing is that we will still continue to have a very, very working relationship, especially when it comes to the QTL. That is the quality teaching and learning network with the rest of the entities. And the entities have to be open, especially in terms of bringing out some of the challenges or some of the issues that need to be addressed, because the QTL is well equipped, especially in terms of some of the current trends, some of the current issues some of the current issues in as far as teaching is concerned, then the other thing is the aspect of moving to a next level where we are taking collaboration at another level where our classes are able to collaborate. That is something that a faculty should take forward. If I'm teaching, I have a class in East Africa that is on microbiology, and there is another lecture in Pakistan, teaching microbiology, even in the same, same semester, then how do we ensure that our classes are collaborating? They have even some similar assignments or tasks that they need to do. Even outside of class, some projects they can be able to do together because the way of education is project or a problem based learning. So that is something that we really have to embrace in terms of moving this kind of online teaching and learning because when students collaborate on projects, even if they were disconnected in terms of online teaching, or they were disconnected somewhere when those online sessions are on, but when they are working on a project, they still have to stay connected. So why don't we give them the projects so that they can be able to work on those projects? It can be a, between countries or between classes or between, campuses. That is something that is where online learning is taking us so that we are able to embrace collaboration.

Speaker 1:

Right. So two aspects that you have talked about. I have noted that down. So another thing is that, how do you see university support or university role in executing, remote teaching and learning program? So you did talk about the entities, which are working over at different departments who are supporting the students and the faculty members. Would you like to add on to this particular question, otherwise we can move ahead.

Speaker 2:

So maybe as QTL, what we are doing is that we are really trying to harmonize this kind of online support. Like if you look at the entities, what you've done is that each and every entity has what we call a VLE assistant, somebody on the ground who is able to help and work with faculty on their VLE. And those are the people we work with them. We do give them workshops. We have a VLE assistant workshops that we ask. We normally schedule like last year, we had about four sessions with them to just introduce to them some of the new things that they could also be able to share with faculty. We also empowered the VLE assistance to be able to lead, take the lead when it comes to the the orientation of students. So we only come in as BDL or QTL to be able to support their orientations, to be able to support them. But what we do is that we sit with them, we have those sessions and we are able to like empower them so that they have those ownership. So the way of teaching support is like on a collaborative kind of an aspect, a collaborative point of view, where we are now able to really collaborate so that we able to promote the best practice. I know entities would be working on their different agenda or things. But when that is harmonized, when it comes to support, we are able to support faculty across. So that is it. We will still continue to support them at working with those people or the divisions on the ground.

Speaker 1:

Okay. All right. So we are on the last question and it's about the SONAM. So how SONAM can be trendsetter or role model in introducing remote learning program to meet countries, nurses, demand and healthcare system.

Speaker 2:

Okay. Okay. Well, some of them, I think I've worked with the instructors in SONAM, and I know that we can tap into their abilities. One of the things that I noted is that they are quite competent in what they are doing, and they are also committed to their tasks. So to empower them is and SONAM being a change maker is how can now SONAM be able to link up with the rest of the entities, because you can't say, it's, it is independent. How are they linking up in medical college'? Are their sessions that they have with the medical college, then how are they linking with the communities? I know we have like medical centers or, or those centers in the country, but how are, is SONAM linking up with those ones in how is SONAM linking up SONAM Pakistan, linking up with the rest of the SONAM's in the world, like Uganda, Kenya, SONAM Tanzania. If they haven't developed that kind of bonding, as I said that, let them now do like team-teaching teach classes, teach some classes in East Africa, if you are teaching, like for example, health assessment in Pakistan have a session even if it is a one hour 30 minutes session for East African students, so that you are able to link that let's see how are those students bring up similar or working on a particular project with those students in Kenya. So if they are able to design those kinds of collaborative activities, then now sort of we'll be able to have taken it's right place in the area of nursing and the area of education. So that is something that, if they want to be changed, because that is how they're going to start initiating change so that we are able to see that they are not only confined in the boundaries of Pakistan, but they are taking whatever skills they have across. We would want to see some of them coming to teach here, even if it is like for one month, two months, face to face, with the Kenyan, we have the Kenyans going to Pakistan, teach for one month. Those kinds of experiences will be able to put a sort of, as a change maker, because I remember in some of my trainings, I was in charge of SONAM, Pakistan, and I train them on the use of VLE from East Africa. So, and it had a bigger impact than if they were even maybe trained by somebody from Pakistan, or they took it up very seriously and I'm so happy with the success that has been realized in SONAM. And we have a very, very good working kind of relationship, it doesn't matter this kind of a black man coming to teach us, but it is like they really respect what I really had to offer in terms of training.

Speaker 1:

Yeah. So, that's all from my side Sir , and thank you so much for taking out time for this interview. Is there anything that you want to say or ask?

Speaker 2:

I never imagined that this session would take one hour and it has taken one hour. I thought I would take 20 minutes at another. Is that all your questions, but I'm so happy. I look forward to having the recording of the same. I also look forward to share it in your research write-up. I also look forward to translating these into another project that we could also be able to do so that we, I am also able to incorporate, to be incorporated in the project maybe as one of the leads, and also be able to challenge our faculty. I look forward to also, being part of the online classes in SONAM. I can come in as an observer so that I'm able to observe some of the lessons and be able to share some of the lessons learned out of the sessions. Yes.

Speaker 1:

Inshallah and we'll soon, share the findings about this research with you. And, if there is anything that you find that you missed or anything like that, you can always write us in a form of a reflective log on the similar ID from where you received the zoom link. And thank you once again for taking out time. So take care

Speaker 1:

Thank you so much and say hi to the team. They all know me just say hi to all of them. Thank you. Thank you. Bye bye. Take care. Bye
