# Supplementary material for: Beyond the classroom walls: Stakeholder experiences with remote instruction in Post RN baccalaureate nursing program during the COVID-19 pandemic: A qualitative inquiry
Source: PLoS One. 2024 Apr 4;19(4):e0300007. doi: 10.1371/journal.pone.0300007 (PMC10994296; doi:10.1371/journal.pone.0300007)
Supplement: S6 File — (DOCX) [file pone.0300007.s006.docx]

**Interviewer: Okay. So, my first question is, what are your views about online teaching and learning?**

**Interviewee:** Are you asking me this as an educator or being the administrative head?

**Interviewer: As a Head person and dealing with all other students and teachers feedback and everything. Is that right?**

**Interviewee:** Yes. So to me, online teaching and learning or online education is a more through which one is unable to see the student, physically and are connected through any available online modality where a teacher and a student is making educational contact. So for the purpose of study and for the purpose of education, a teacher and student are connected online. It is one of the recent contemporary modes of teaching and learning where distances physical presence is not a barrier to higher education or any education. So it is a flexible approach that enables anyone to pursue education through any institution that they want to. And being in the same city, being in the same campus and being close by is not a barrier anymore.

**Interviewer: Right. So according to you, it is a flexible approach where a student and a teacher can interact and work alone. So, my next question is that what are your experiences of supporting the migration of existing curriculum to remote teaching and learning during the pandemic and how can faculty and students be supported in this transition?**

**Interviewee:** Okay. So, can you read the first part of your question again?

**Interviewer: Yes, it is. What are your experiences of supporting the migration of existing curriculum to remote teaching and learning during the pandemic?**

**Interviewee:** Okay. So, my experience in supporting was that when COVID struck and the higher education commission had strict regulations about not having physical classes ensuring student safety, we as a university abided by those rules. And first of all, when we said that we have the permission of going online, as faculty members, we were all prepared because we were already teaching on the blended learning mode and we had the skills and knowledge necessary to pursue online education in the full format.

So the faculty was ready, but the experience in terms of student access was the biggest challenge that I had to face as the head of the program, a very high percentage of our students live in the Northern areas and getting access for them and getting in contact with them was a challenge. So, we had the courses ready, our faculty was prepared, they were trained to put up the courses. We had somebody refresher courses done for the faculty members. We arranged for the resources for faculty to record their lectures, to add videos to their lectures and prepare online teaching modules. But the students did not have access. So, in terms of specially the four-year BSCN, and are you focusing on the four-year BSCN or o RN program or both?

**Interviewer: Post RN.**

**Interviewee**: Okay. So in Post-RN very few students did not have access. And what we did was that we called up every student on the telephone and we emailed and used a snowball networking to find out if the students had internet. And if they could connect online for the students who did not have internet, we offered them that, you know, the university could pay for some data bundles. If they thought that they could buy the data bundles. And if that was not an option they were assigned to an Agha Khan education services school in nearby areas where they were assigned days and timings, and they could walk to those schools where internet and a computer lab was available for students to use internet as well as the hardware, if they did not have a computer or a laptop at home. So this way we were able to connect those students post RN’s, may it was not too big an issue because most of the students had internet connection as they were belonged to Karachi and people who were in the North, but either working or employees of AKU or somewhere else where they could go and use the internet. So, that was, how we started. Then we started teaching online. The students came regularly to the classes we had, online teaching, both synchronous and asynchronous, where, we had zoom sessions where students could interact. And there were times when students had to do self-directed learning modules and answer on discussion forums and post assessments.

So, it was a new change for the students, and they enjoyed it. The teachers were learning, the students were learning. The assessments were also new. The teachers had to learn how to give complete assessments in an online modality. So we were trained on teaching in a blended format, but assessments so far were done in the traditional way where students would come to the classroom and attempt their exams and the scholarly papers and other presentations were also face to face. So, this time it was where students were on either zoom or they had to give an online exam. And, they could still submit their scholarly papers through email. So, these were some of the experiences. And, and when we started to do these things, there were hitches where students could not connect continuously. They had interruptions, they were not happy with time bound exams because each MCQ that they had to attempt was timed and they had to speed up, whereas in face to face, they are kind of, you know, used to having a time where you, when you distribute papers to the whole class, they get extra time to read. When you are submitting the papers back, they get extra time to read, but in a virtual environment, the computer shuts and goes off. And this was a kind of anxiety provoking for the student and they were kind of getting used to all this.

And we also had backup systems where we could check if the student did face genuine technical issues, we supported them. if they were technical reasons, we could offer them another exam, another paper, or we also allowed them to come to the campus and use internet services for their assessments, because they were a small group until then HEC had allowed people to come in very small groups. So, these are some of the important areas that we were able to work on.

**Interviewer: Okay. So, you have very well explained about how you supported. I just want to ask that, uh, how can, like, is there any better way to support this transition or are there any further plans, if God forbid, if we go back to the same thing again, or if any, such other pandemic happens. So any other planning?**

**Interviewee:** The planning would be that this time we have, we would tell the students that they have to make a conscious arrangement of where they are going to study and how they are going to study. So, they need to be prepared with their own device, with their own internet. And if they need any financial or moral support in determining that we are there to help them. But as university students, I think they also need to be cognizant that this is their learning and they need to be responsible.

If the university's allowing them to come in smaller groups, people who really need internet support and do not have it at home should come ahead of time, placed themselves in an environment where they are safe, but they can get good internet connection and offer their exams and tests. So, it has to be a pre prepared thing because the students have been through it. They know what kind of issues they can encounter. As a faculty, we know what kind of issues we can encounter. So, everybody needs to have a better backup plan this time.

**Interviewer: Right. So, let's move to the next question. And the next question is that what are the advantages of online teaching?**

**Interviewee:** So, the advantages are, like we said, it's flexible. You can be anywhere and get connected. You can work and get connected and do online learning and reading in your own time because it's on the web. You can always access it based on your convenience. On the other hand, I think it is little easy on cost also. Where you are spending on Wi-Fi, on the other side you are saving on transport money because you are doing all the working from home. 24 seven time schedule available here for you to respond. And even for the faculty, for suppose if they are busy in something during the day time, so they can respond in the evening if they are free. maybe after hours they can connect with the students, the weekends are also available, sometimes it is difficult for students to connect so they have time flexibility, resources flexibility, learning flexibility are some of the advantages of online learning.

**Interviewer: Okay. anything about balance with work? Family? anything regarding that?**

**Interviewee:** So, you know, like I said, you can balance your job and your study, you can balance your home and your study and if you have children and family responsibilities during the day, you can handle those and do your studies in the night. That could be your preference time.

**Interviewer: Okay.**

**Interviewee:** So, for the mothers especially when the kids are in school, they want to study, so they could utilize those time slots for studying purpose.

**Interviewer: And what are your thoughts about disadvantages of online teaching and how can these disadvantages or challenges be mitigated? So first you can talk about the disadvantages and then we can move forward.**

**Interviewee:** So, disadvantages are few, so a physical and face to face eye contact is always missed by a teacher and learner. When you look at each other's in the eye and you do nonverbal gestures like nodding and smiling, it is fruitful for a student to see that the teacher is engaged and it's fruitful for a teacher to know that the students are learning and, are kind of connected, non-verbally with the teacher, which is not available online. Even in zoom sessions, you can't keep an eye on the whole class. So, that's there. One is not even sure that they in any session the student has logged on and is there or has gotten busy with other things that are there.

So, keeping the student engaged is a challenge and a disadvantage, and that needs the teacher's preparation to prepare the class in a manner that student engagement is ensured and learning outcomes are met. The strategies are placed in a way that there is a, you know, there is online student engagement that are small learning outcomes spread in a bigger time, so that you keep assessing the student.

As if like if there has been a session of two hours, then as a teacher you would have to include some strategies to enable you to know as how much the student has grasped and as how much the student could study. The pre reading and the reading material has to be, user-friendly not very long, not very boring. So that when the student is reading it from the screen or when the teacher is not there. So, it is easy for them to read and understand as to what is expected of them. The guidelines have to be clear, because there is an opportunity in the class that you can raise your hand and ask a question on the campus with some other student or teacher. So, online many possible questions that the student may have, should be clearly put-in, as clear as possible guidelines for the student goal in the class participation, for assignments, for the assessments and everything. The grading has to be prompt. The time schedule has to be maintained and as where and at what time, with which pass-word it has to be logged -in. And till when and what duration the exams would be done. There needs to be a to-do list of everything as which you do in the face-to-face interaction. It has to be written down in the websites for students to use.

**Interviewer: Okay. You have answered my next question as well, but I will repeat that question. If there is anything that comes in your mind, you can talk about that. So, what are the competencies or skills faculty members required to teach online during the crisis? Earlier you said that your faculty members were prepared to go online, and then now you said that they should know how to engage students as what the interactive way should be. So, are there any other skills or competencies that a faculty member should require?**

**Interviewee:** So, yes. the capacity building around online teaching. So blended, we were well-versed but how to do the complete the things online without meeting the students at all. So, teaching online is one, assessing online is one, and then grading and sharing those grading and feedback with the student is one thing. So, our faculty has to be very versed and expert in doing all the three phases. The queries, concerns of the students as how and when are they going to be available, is another thing. So, to ensure online engagement is an area that all faculty members will need to be well-versed with. And, then to see as how to manage each and every strategy as how to have the pre reading done or as how to give the guidelines as it comes in the literature as how do you prepare a valid and reliable and a non-Google able assessment strategy in your online teaching? So, the teaching has to be done in a manner that the learning outcomes are met, and the assessment has to be done in a manner that you are able to assess as how the student has read and understood it. And as how to grade them by making sure that the objective has been met.

**Interviewer: Alright. And as you also mentioned the effective ways. Are there any ways that are in your mind, or like how can a faculty effectively engage students in the online learning environment?**

**Interviewee:** So, the faculty has to be aware of the various strategies like as in what kind of strategies can be in that sort of topic, and how the student can be engaged. For example, as we give a break in between a session and when after the break we recontinue the session and the students are seen online in the list, there should be a small check done to see that all the students have returned by having them to raise their hands. So if someone has just logged in and gone he / she will not know that you have asked them to raise their hand, so you have to be prompt and energetic yourself to be able to see as what the students are doing by asking random questions and then once or twice asking the students to open their cameras, to see if they're there or not, and they are enjoying or not, invite any questions or queries, give simple instructions, give and teach, not for very long hours, but ensure that the students concentration is maintained for 20 to 40 minutes, and then an exercise is done to keep them busy and not let them get bored.

**Interviewer: And as also using the features effectively as you had spoken of the raising of the hands. You should know all those features. Okay. So, now moving to the next question and it's regarding the remote areas. So, what are your views regarding use of online teaching and learning for the students residing in remote areas?**

**Interviewee:** So, for the remote areas remote strategies would be used. Where study packs are sent to the student or the study packs can be sent to a centralized place from where the student can pick and then submit it there too. So just as there are strategies for online and blended learning, same way there are strategies of remote teachings, also. So again, the principles are the same that as your teaching material has to be precise, clear, readable, downloadable, printable. So, all these options should be with the student. The assessment should be made in a form which would not be directly available on Google. And the application or comprehension should be on the level of the synthesis and analysis which ensures that if the student has not pre-read and understood then he/ she would not be able to answer it. The way as how to answer and how the guidelines shall be done, then the marking criteria, rubric. Everything has to be very clear for the student to understand and attempt, and then submit on a point where from where a teacher can receive and correct it.

**Interviewer: Okay. So, can you please share some of the experiences that happened with the students who were residing in the remote areas in pandemic times?**

**Interviewee:** So, there was an experience of this in the pandemic times but not with the post-RN.

**Interviewer: Okay.**

**Interviewee:** The post RN were either able to connect online directly or indirectly but the study packs were not sent to the post RN.

**Interviewer: Alright. So, were all residing here in the city?**

**Interviewee:** Most of them were in Karachi. Or they were able to get access on internet through schools or through neighbors or somewhere through WhatsApp or their own phones.

**Interviewer: Okay, okay. Okay. So, the next question is that what are your views regarding complete shift or migration to online modality, even if the pandemic gets over and after that?**

**Interviewee:** So, it is a good idea, especially in the post RN BSCN program where most of the students who want to join post RN are working somewhere or the other or are raising families. So it will be a very good idea to go online completely, but they really have to be strategies planned to make sure that because this is a healthcare degree, it will require a lot of clinical hands-on work. So, somehow how this online work will be adjusted, will have to be sorted because it's an important component of higher education in nursing. So strategies like simulation and strategies like as if I am working in Chitral or Gilgit somewhere, Or if I am in America or any other place, and I want to pursue online then I should have one such option where then an hospital can be identified as where there is availability of preceptors. So that my teacher could be online facilitating me for a clinical component, and I have a preceptor on ground as from which country or city, I am working from. So, working with those connections and working with that preceptor, and that I can work as a trio model of faculty, preceptor, and a student.

**Interviewer: Okay. And what about the resources? Do you think that, the Institute is prepared enough to further this idea?**

**Interviewee:** For post-term BS CN? Yes, we are prepared, and we just need to finalize the processes because we have already piloted it on a few students who were not able to come back and work here. So, there is a case study where a student was in Canada and she had a preceptor there and we had facilitated her online and had her practical done. So, for post RN, I think we are ready. The student needs to be ready and be available and willing and to take these flexible options.

**Interviewer: Okay. Thank you for the answer. Okay. The next question is regarding assessments and grading, you discussed about some of the experience, but I would like to repeat if there is anything left, or any other thoughts, please share. So, what are your experiences of supporting the planning of students assessment and grading in a complete online learning environment during pandemic?**

**Interviewee:** So, I think I've covered most of the points where I said that, you know, the guidelines have to be such the question construction has to be valid and reliable. The timings, the guidelines, as briefing to students about what to expect. I think these are some of the major areas that I've already talked about.

**Interviewer: Okay. And were there any issues that were reported during the pandemic time regarding assessment and grading from students or faculty site? Any sort of challenges?**

**Interviewee:** Yeah, there were challenges, we needed a system building. So, they were, first of all, the students were, some of them were really surprised at how they were not able to gain good marks. And when they looked at the questions, they realized that, you know, probably they didn't give attention to what readings were given. Some of them faced technical issues. And if we were able to trace technical issues, we gave them another option of doing a retest or the re exam to be fair. Some of them did not report technical issues, but when the results were shared, they came up saying that they had technical issues. And, but we did not entertain them because if they were told to report it then and there, and they did not do it, then it was not taken care of.

**Interviewer: Okay. Right. This is a general question regarding the challenges that you faced in implementing online teaching. So, keeping in mind assessment or grading or any other like, well teaching and learning process, were there any challenges that you people faced in implementation?**

**Interviewee:** So, connectivity issues, electricity issues, which are part of our context, were at the top. So because the students, when they started the program, they were not ready. They did not know, the teachers did not know that COVID is going to come and we will have such issues. So, on the first go preparing to teach online was a challenge. But I think as it went ahead, it became better and systems were kept in place. And the virtual learning environment was strengthened. The resources were provided. The student got used to connecting to the teacher without meeting them. And I think with each experience it's getting better.

**Interviewer: Okay. So, we are left with a few more questions. So, what are your recommendations to ensure sustainable remote teaching and learning in the future?**

**Interviewee:** Like?

**Interviewer: The guidelines and all, any other thing that you think should be recommended?**

**Interviewee:** I think our whole guideline at the university level is going to be helpful and we are making it too. There had been a group made for remote and online teaching. For which we have guidelines and for which a manual has also come which guides the faculty online teaching strategies or choosing use on each idea. So, I think when such resources are on a university level then avenues would be made where the students will be able to borrow hardware and internet from the university. So, I think the systems are made then all these things would be more sustainable.

**Interviewer: Okay. And was there any model that you people followed for online teaching and learning?**

**Interviewee:** No. It was either a normal curriculum model, which was, which is on the paper was translated into an online format.

**Interviewer:** **Okay. So, the next question is how do you see university support or role in executing, remote teaching and learning program?**

**Interviewee:** I think I have answered that too, a bit.

**Interviewer: Right. Okay. And the last question is how Sonam can be a trendsetter or a role model in introducing remote learning program to meet countries, nurses demand in healthcare system?**

**Interviewee:** I think the answer to post-RN would be that we go completely online, and we invite people at the time of their admissions to identify a placement and a preceptor to whom they will work from. And so, this program could be strengthened so that wherever you are in the world and if you have a clinical placement available with you and that you have an eligible preceptor which we can make as your criteria then the practical and theory can be completely taken care of as where you are located in, you need not come to the campus online or need to campus at all.

**Interviewer: So, like, do you really see Sonam as a trendsetter or like it takes, it will take some time, some years to become a role model?**

**Interviewee:** It said, I think the ground is set and we have been marketing it. And we do it for our students too that integrated clinical objectives can be made and if one is an employee and he / she wants to work from their workplace then we do have the flexibility. We have tested different models where the students keep working as staff nurses, and we give them additional objectives and additional hours to work on a few things and produce assignments and discussions where their competency could be increased and they could do a work based program online.

**Interviewer: Okay. I think that's all from my side. And thank you so much for taking out time. Are there any questions from your side?**

**Interviewee:** No, I think I'm good.

**Interviewer: Okay. Thank you so much. Again, take care. Nice talking to you. Bye.**

**Interviewee:** Likewise. Thanks. Bye. Bye.
